# Light-Driven Ring Slippage in [Re(η^7^-C_7_H_7_)(η^5^-C_7_H_9_)]^+^ and the Inertness of Its Technetium Homologue

**DOI:** 10.1021/acs.inorgchem.3c04052

**Published:** 2024-01-22

**Authors:** Federica Battistin, Robin Bolliger, Manuel Luca Besmer, Thomas Fox, Olivier Blacque, Henrik Braband, Roger Alberto

**Affiliations:** Department of Chemistry, University of Zurich, Winterthurerstrasse 190, Zurich 8057, Switzerland

## Abstract

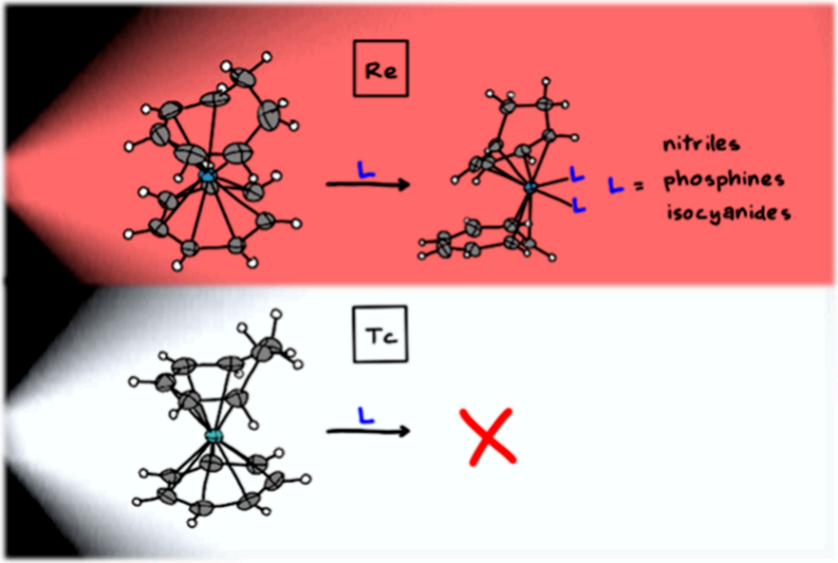

Here, we present the light-driven reactions of [Re(η^7^-C_7_H_7_)(η^5^-C_7_H_9_)]^+^ (**1**^+^) with nitriles, phosphines, and isocyanides, which are added to **1**^+^ via a ring slippage of the tropylium cation from η^7^ to η^3^, forming [Re(η^3^-C_7_H_7_)(η^5^-C_7_H_9_)(L)_2_]^+^ (L= acetonitrile **2**^**+**^; 2-phenylacetonitrile **3**^+^; 1,3,5-triaza-5-phosphoadamantane (PTA) **4**^**+**^; *tert*-butyl isocyanide **6**^+^; benzyl isocyanide **7**^+^) and [Re(η^3^-C_7_H_7_)(η^5^-C_7_H_9_)(L)]^+^ with L = (ethane-1,2-diyl)bis(diphenylphosphane) (dppe) **5**^+^. To compare the reactivities of rhenium and technetium, we also investigated the synthesis of [^99^Tc(η^6^-C_10_H_8_)_2_]^+^, its substitution of naphthalene with cyclohepta-1,3,5-triene to obtain [^99^Tc(η^7^-C_7_H_7_)(η^5^-C_7_H_9_)]^+^, and its reactivity (or lack thereof) with light.

## Abstract 2

We describe the remarkable photochemical behavior of [Re(η^7^-C_7_H_7_)(η^5^-C_7_H_9_)]^+^ under red light and excess of ligands such as nitriles, phosphines, isocyanides. This leads to the formation of [Re(η^3^-C_7_H_7_)(η^5^-C_7_H_9_)(L)_2_]^+^ via a ring slippage mechanism. Surprisingly, the technetium analogue ([^99^Tc(η^7^-C_7_H_7_)(η^5^-C_7_H_9_)]^+^) displays a different electronic structure compared to the rhenium complex and is completely inert toward nitrile addition under light irradiation or at 65 °C, challenging conventional reactivity expectations.

## Introduction

**Scheme 1:**
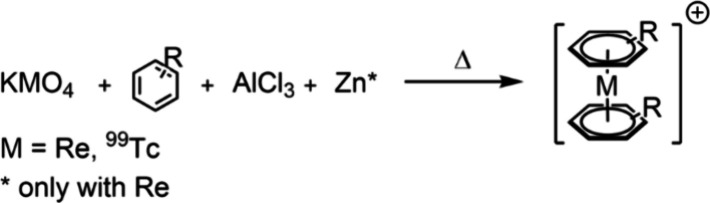
Synthesis of Rhenium and Technetium bis-Arene via the Fischer–Hafner Approach

The chemistry of {M(η^6^-arene)}^+^ (M = Re, ^99^Tc) fragments remains a relatively unexplored area due to the limited availability of rhenium and technetium precursors. The conventional method for synthesizing [M(η^6^-arene)_2_]^+^ (M = Re, ^99^Tc) complexes via the Fischer–Hafner approach employs Zn/AlCl_3_ as activation/reduction agents, along with an excess of arene and [MO_4_]^−^ (M = Re, ^99^Tc, Scheme 1). This method is only effective for arenes bearing no Lewis basic substituents.^1^ We have already detailed the preparation of [Re(η^6^-C_6_H_6_)_2_]^+^ and [^99^Tc(η^6^-C_6_H_6_)_2_]^+^ using this established approach by adapting a procedure previously reported by Kudinov et al. some years ago.^2^

**Scheme 2:**
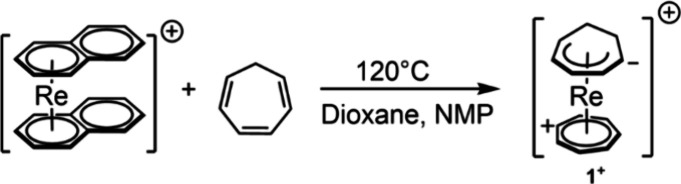
Synthesis of [Re(η^7^-C_7_H_7_)(η^5^-C_7_H_9_)]^+^ by Naphthalene Exchange in [Re(η^6^-C_10_H_8_)]^+^ with Cyclohepta-1,3,5-triene

Notably, these “sandwich” compounds exhibit exceptional stability in both water and air, in stark contrast to their counterparts with neighboring elements. As compared to benzene-type sandwiches, bis-naphthalene systems [Re(η^6^-C_10_H_8_)_2_]^+^ are more useful due to the relatively easy displacement of the coordinated and weaker bound naphthalene ligands.^3−5^ Accordingly, we have recently reported on naphthalene exchange in [Re(η^6^-C_10_H_8_)]^+^ with seven- and eight-membered rings bearing multiple double bonds such as cyclohepta-1,3,5-triene, tropone and cycloocta-1,3,5-triene.^6^ These complexes were known for the neighboring elements, but unknown for rhenium and technetium. We described the preparation of [Re(η^7^-C_7_H_7_)(η^5^-C_7_H_9_)]^+^ in particular (**1**^+^, Scheme 2): after coordination to the rhenium center, two cyclohepta-1,3,5-triene molecules rearranged with a formal migration of a hydride from one coordinated C_7_H_8_ to the opposite one. This reactivity pattern has also been described for group 4 elements, Mo and W, but not with rhenium or technetium.^7^

We describe herein light-driven reactions of [Re(η^7^-C_7_H_7_)(η^5^-C_7_H_9_)]^+^ with monodentate and bidentate ligands such as acetonitrile, isocyanides, 1,3,5-triaza-7-phosphoadamantane (PTA), and (ethane-1,2-diyl)bis(diphenylphosphane) (dppe).

To cross-compare reactivities of rhenium and technetium, we also investigated the preparation of [^99^Tc(η^6^-C_10_H_8_)_2_]^+^, its naphthalene exchange with cyclohepta-1,3,5-triene for obtaining [^99^Tc(η^7^-C_7_H_7_)(η^5^-C_7_H_9_)]^+^ and its (non)-reactivity with light.

## Result and Discussion

**Figure 1:**
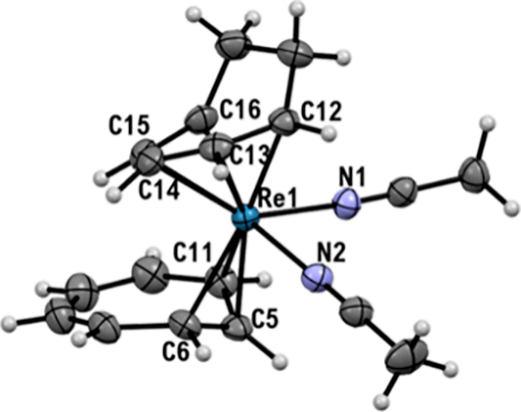
Ellipsoid displacement plots of [Re(η^3^-C_7_H_7_)(η^5^-C_7_H_9_)(NCCH_3_)_2_]PF_6_ (**2PF**_**6**_). Ellipsoids are drawn at 50% probability. The PF_6_^**–**^ anion is omitted for clarity. Coordination distances in Ångström (Å): Re1–C5 = 2.145(3), Re1–C6 = 2.236(3), Re1–C11 = 2.317(3), Re1–C12 = 2.220(3), Re1–C13 = 2.255(3), Re1–C14 = 2.268(3), Re1–C15 = 2.184(3), Re1–C16 = 2.220(3), Re1–N1 = 2.097(2), Re1–N2 = 2.128(2).

When leaving a solution of [Re(η^7^-C_7_H_7_)(η^5^-C_7_H_9_)]^+^ in acetonitrile-*d*_3_, we observed a gradual color change from green to yellow. The ^1^H NMR spectrum revealed a new set of signals together with those of complex **1**^**+**^. Intrigued by this behavior, we explored the stability of **1**^**+**^ in different deuterated solvents but no changes in the ^1^H NMR spectra were detected in acetone-*d*_6_ and methanol-*d*_4_, only in acetonitrile-*d*_3_. We decided to investigate the behavior of compound **1**^**+**^ under light irradiation and in the presence of acetonitrile using a homemade light-reactor based on a LED lamp inside a box (for details, see the Supporting Information). [Re(η^7^-C_7_H_7_)(η^5^-C_7_H_9_)]^+^ was dissolved in methanol-*d*_4_, in a NMR tube, and 20 μL of acetonitrile was added. The NMR tube was irradiated with red light (629 nm, 10 W), and the reaction was monitored by ^1^H NMR. Already after 1 h, new signals appeared in the ^1^H NMR spectrum, while the color of the solution changed gradually from green to yellow. The signals of the starting complex disappeared completely after 52 h and only one defined set of signals was left (*vide infra* for the NMR characterization). The nature of the complex was elucidated by single crystal X-ray analysis, which shows a rhenium complex with the cycloheptadienyl anion still bound in an η^5^-fashion to the rhenium center, the tropylium cation hapticity-shifted to η^3^ and two acetonitrile molecules (Figure 1). Interestingly, the Re–N bond lengths differ significantly: the Re–N1 distance is 2.097(2) Å, which is in agreement with the one reported for Re(I) acetonitrile complexes,^8^ while Re–N2 is significantly longer (2.128(2) Å).

**Scheme 3:**
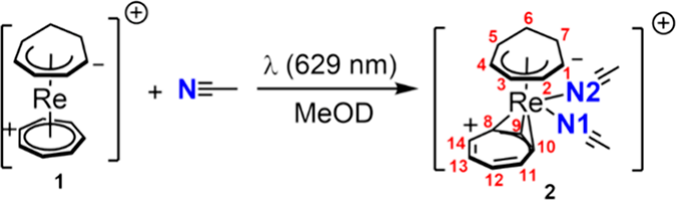
Addition of Two Molecules of Acetonitrile to Compound **1**^**+**^ Triggered by Red Light Forming of [Re(η^3^-C_7_H_7_)(η^5^-C_7_H_9_)(NCCH_3_)_2_]^+^ (**2**^**+**^, with Numbering Scheme)

Mechanistically, two molecules of acetonitrile were added to **1**^**+**^, inducing the ring slippage of the tropylium cation from η^7^ to η^3^, thereby forming [Re(η^3^-C_7_H_7_)(η^5^-C_7_H_9_)(NCCH_3_)_2_]^+^ (**2**^**+**^) (Scheme 3).

There are a few examples of a tropylium cation bond in an η^3^ manner: [M(η^3^-C_7_H_7_)(CO)_3_] (M = Co(I), Fe(II), Ru(II), Os(II)) and [Mo(η^3^-C_7_H_7_)(η^5^-C_5_H_5_)(CO)_2_].^9−12^ However, while they all display (η^3^-C_7_H_7_)^+^ coordination in the crystal structure, all exhibit a fluxional behavior of the tropylium cation in solution. To the best of our knowledge, the reactivity of [Re(η^7^-C_7_H_7_)(η^5^-C_7_H_9_)]^+^ described above is unprecedented within this type of sandwich structure. The only similar reactivity pattern reported is with [Zr(η^7^-C_7_H_7_)(η^5^-C_7_H_9_)]. In the presence of PMe_3_, it forms [Zr(η^7^-C_7_H_7_)(η^5^-C_7_H_9_)(PMe_3_)] but the tropylium cation does not change its hapticity and retains its coordination geometry and binding mode.^13^

The same reaction as described above was performed at different wavelengths: it required 5 days of irradiation with orange light (514 and 621 nm, 10 W) and 7 days with white light (444, 515, 562, and 608 nm, 10 W) to convert **1**^**+**^ into **2**^**+**^.

The formation of [Re(η^3^-C_7_H_7_)(η^5^-C_7_H_9_)(NCCH_3_)_2_]^+^ must be light-induced as heating **1**^**+**^ in acetonitrile at reflux and light protected for 24 h does not convert it into **2**^**+**^. The ^1^H NMR spectrum shows unreacted **1**^**+**^ and other small signals of unidentified species.

**Figure 2:**
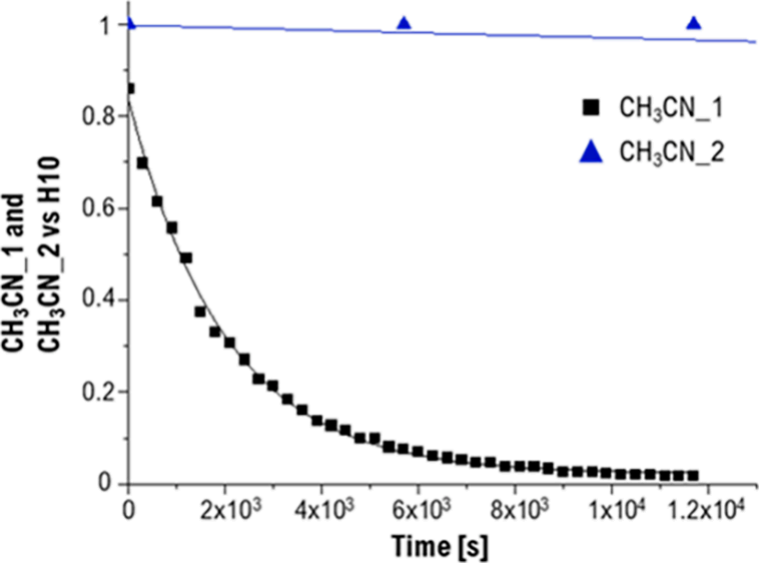
Comparison of nitrile exchange kinetics of [Re(η^3^-C_7_H_7_)(η^5^-C_7_H_9_)(NCCH_3_)_2_]^+^ (**2**^**+**^).

To assess if the acetonitrile ligands in **2**^**+**^ are labile, nitrile exchange kinetic experiments (CH_3_CN/CD_3_CN) were performed by following the decrease of the integrals of the ^1^H NMR signals of coordinated CH_3_CN ligands in **2**^**+**^ in CD_3_CN. The integrals were referenced against the H10 signal at 1.43 ppm (numbering scheme shown in Scheme 3). Interestingly, the two CH_3_CN ligands display different exchange rates (Figure 2): the signal at 2.46 ppm disappears after 4 h (exchange rate of 4.98 × 10^–4^ s^–1^ ± 9.81 × 10^–6^ s^–1^ pseudo-first-order in pure acetonitrile-*d*_3_, Figure S34, 2.62 × 10^–5^ s^–1^ M^–1^ ± 5.16 × 10^–7^ s^–1^ M^–1^ second-order rate constant). For the second signal at 2.72 ppm, it took 22 days for full exchange with deuterated acetonitrile (2.78 × 10^–6^ s^–1^ ± 5.16 × 10^–8^ s^–1^, Figure S34, 1.46 × 10^–7^ s^–1^ M^–1^ ± 2.72 × 10^–7^ s^–1^ M^–1^ ditto). Based on the bond distances between Re and acetonitrile, we could suggest that the upfield shifted signal belongs to the acetonitrile with the slightly longer bond and the faster exchange rate. Both exchange rates are significantly slower compared to the Re(I) complex [Re(η^6^-C_6_H_6_)(NCCH_3_)_3_]^+^, which is so fast that it is not detectable by NMR since the exchange is already complete within the time frame required for sample preparation and measurement.^8^

We also investigated the reversibility of the light-driven reaction by exposing [Re(η^3^-C_7_H_7_)(η^5^-C_7_H_9_)(NCCH_3_)_2_]^+^ to blue light (465 nm, 10 W) in methanol-*d*_4_ and monitoring ^1^H NMR spectra after distinct times. After 22 h of irradiation, **2**^**+**^ was indeed fully reconverted to **1**^**+**^. The process was found to be irreversible if performed in the presence of an excess acetonitrile (i.e., by keeping freshly prepared **2**^**+**^ in the same NMR tube, without removing deuterated methanol and acetonitrile and irradiated it with blue light). Complex **2**^**+**^ appears to be in an equilibrium with **1**^**+**^, which is light- and ligand-triggered: exposing [Re(η^7^-C_7_H_7_)(η^5^-C_7_H_9_)]^+^ to red light with an excess of acetonitrile converts it to [Re(η^3^-C_7_H_7_)(η^5^-C_7_H_9_)(NCCH_3_)_2_]^+^. However, when **2**^**+**^ is dissolved in methanol and exposed to blue light, it rapidly converts back to **1**^**+**^ in the absence of acetonitrile.

**Scheme 4:**
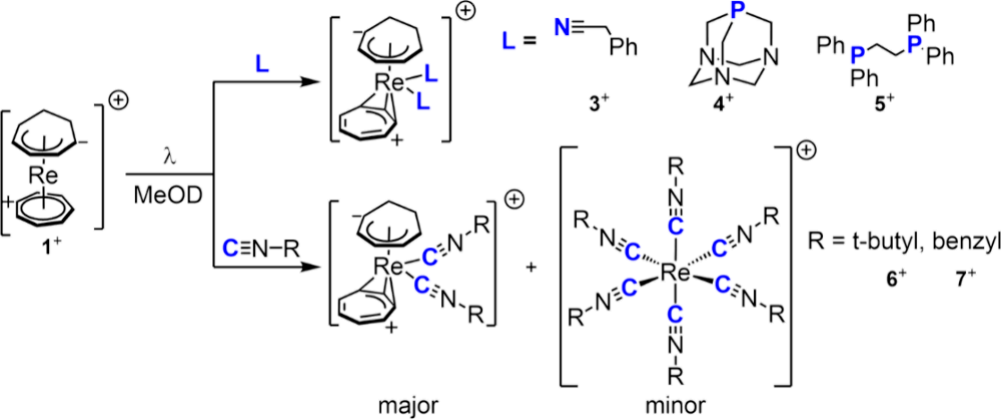
General Scheme of Light-Induced Addition of Monodentate and Bidentate Ligand to [Re(η^7^-C_7_H_7_)(η^5^-C_7_H_9_)]^+^

For assessing if these ring-slippage-ligand exchange and light-induced reactions are of a more general nature, we replaced acetonitrile by 2-phenylacetonitrile (PhCH_2_CN). Indeed, similar to acetonitrile, the complex [Re(η^3^-C_7_H_7_)(η^5^-C_7_H_9_)(PhCH_2_CN)_2_]^+^ formed within 6 days (Scheme 4). Reactions with 1,3,5-triaza-7-phopshoadamantane (PTA) and (ethane-1,2-diyl)bis(diphenylphosphane (dppe) proceeded similarly giving [Re(η^3^-C_7_H_7_)(η^5^-C_7_H_9_)(PTA)_2_]^+^ (**4**^+^) and [Re(η^3^-C_7_H_7_)(η^5^-C_7_H_9_)(dppe)]^+^ (**5**^+^), respectively (Scheme 4). The reactions proceeded at different rates. While **4**^+^ was formed within 2 days, the synthesis of **5**^+^ required 15 days of irradiation. The UHPLC-MS analyses of these reactions show masses *m*/*z* of the product (685.16 *m*/*z* and 769.23 *m*/*z* for PTA (**4**^**+**^) and dppe (**5**^**+**^), respectively).

Reactivities with isocyanides were strongly ligand concentration dependent. With a small excess (8–10 equiv) of isocyanides, the expected complex [Re(η^3^-C_7_H_7_)(η^5^-C_7_H_9_)(CN-R)_2_]^+^ (with R = *tert*-butyl, benzyl) formed in 48–72 h, while a large ligand excess (65 equiv) led to a mixture of [Re(η^3^-C_7_H_7_)(η^5^-C_7_H_9_)(CN-R)_2_]^+^ (major) and the homoleptic complexes [Re(CN-R)_6_]^+^ (R = *tert*-butyl **6**^**+**^, benzyl **7**^**+**^) in a small amount (Scheme 4). In fact, the UHPLC-MS analysis of these reactions showed a mass of the [Re(η^3^-C_7_H_7_)(η^5^-C_7_H_9_)(CN-R)_2_]^+^ type of product (537.17 *m*/*z* and 605.15 *m*/*z* for R = *tert*-butyl and benzyl respectively) but also 685.40 *m*/*z* and 889.32 *m*/*z* corresponding to the [Re(CN-R)_6_]^+^ with R = *tert*-butyl and benzyl, respectively.

The reversibility of the described light-driven reactions was investigated with **3**^**+**^–**7**^**+**^ as well. Only the nitrile complex **3**^**+**^ reconverted to **1**^**+**^ within 80 h under blue light. The other complexes **4**^**+**^**–7**^**+**^ did not display any reactivities with blue light.

[Re(η^7^-C_7_H_7_)(η^5^-C_7_H_9_)]^+^ did not react with CO probably due to its low concentration in solution and not being as good of a Lewis base as the other ligands. In addition, no reactivity was observed with sulfur-based ligands such as dmso or 1,2-bis(methylthio)ethane.

### NMR Experiments

Due to the asymmetric environment in **2**^**+**^, the ^1^H NMR spectrum of [Re(η^3^-C_7_H_7_)(η^5^-C_7_H_9_)(NCCH_3_)_2_]^+^ presents one set of seven signals for the tropylium cation, one for each proton of the molecule, one for the cycloheptadienyl anion, and one for each acetonitrile ligand. Assignments were done by ^1^H–^1^H COSY and ^1^H–^13^C HSQC (Figure S9 and S10) analyses (numbering scheme is shown in Scheme 3). The anion (C_7_H_9_)^−^ has five multiplets of the η^5^-bound C–H to the rhenium respectively at 6.27 (H2), 6.13 (H5), 5.69 (H4), 5.61 (H3), and 4.78 ppm (H1, covered by the water signal) that account for one proton each. Two signals are found for the protons *endo* of the methylene groups at 3.91 and 2.37 ppm while the *exo* protons are at 2.09 and 2.02 ppm (the last resonance covered by the signal of free acetonitrile). The tropylium cation ([C_7_H_7_]^+^) has two triplets at 5.51 and 5.40 ppm for two protons of the C–H bound to the rhenium (H8 and H9 respectively), while the third one is highly upfield shifted at 1.43 ppm (H10) since it is below the (C_7_H_9_)^−^ anion and feels its shielding effect. The resonances of unbound C–H protons are at 3.75 (H11), 3.70 (H14), 2.16 (H13), and 2.09 (H12) ppm (the last two overlapping with the satellite signal of the free acetonitrile and the CH_2_ signal of the (C_7_H_9_)^−^ anion, respectively). Additionally, two singlets of the methyl groups of each acetonitrile are found at 2.85 (CH_3_CN_2) and 2.60 (CH_3_CN_1) ppm.

The ^1^H NMR spectrum of **3**^**+**^ is very similar to the one of **2**^**+**^, with one set of signals for the tropylium cation (one for each proton of the molecule), one for the cyclopentadienyl anion, and one for each 2-phenylacetonitrile molecule. Assignments were done with the help of ^1^H–^1^H COSY and ^1^H–^13^C HSQC (Figure S12 and S13).

The ^1^H NMR spectra of the other complexes are quite different. For both phosphine and isocyanide complexes, most of the resonances of the tropylium cation are missing. In the case of **6**^**+**^, when lowering the temperature to 235 K, six broad singlets appear between 6.5 and 3.9 ppm in the ^1^H spectrum in methanol-*d*_4_ (Figure S20). They can be assigned to the tropylium cation, meaning that at 298 K, the tropylium cation is highly fluxional and its signals are not visible due to coalescence. This behavior has been reported for (C_7_H_7_)^+^ coordinated in an η^3^ manner in [M(η^3^-C_7_H_7_)(CO)_3_]^−^ (M = Ru(II), Os(II)) and [Mo(η^3^-C_7_H_7_)(η^5^-C_5_H_5_)(CO)_2_].^11,12^ At lower temperatures (158 K for [Os(η^3^-C_7_H_7_)(CO)_3_]^−^ and 166 K for [Mo(η^3^-C_7_H_7_)(η^5^-C_5_H_5_)(CO)_2_]), the singlet disappears and 4 more resonances are found between 6.5 and 1.0 ppm.

Due to the low solubility of the phosphine compounds **4**^**+**^ and **5**^**+**^, it was not possible to record low *T* spectra of these complexes. Nevertheless, since their ^1^H spectra at 298 K are similar to the one of **6**^**+**^ (i.e., missing tropylium cation resonances), we assume that in these cases, the tropylium cation is highly fluxional as well.

Finally, the ^31^P spectra of [Re(η^3^-C_7_H_7_)(η^5^-C_7_H_9_)(PTA)_2_]^+^ (**4**^**+**^) and [Re(η^3^-C_7_H_7_)(η^5^-C_7_H_9_)(dppe)_2_]^+^ (**5**^**+**^) both show two doublets, one signal for each phosphorus atom at −87.5 and −106.5 ppm for **4**^**+**^ and at 19.52.0 and 16.16 ppm for **5**^**+**^.

### X-ray Structures

**Figure 3:**
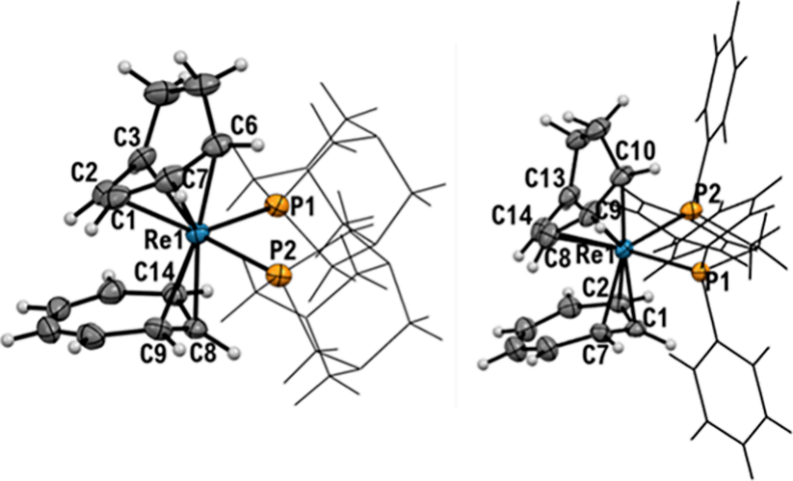
Ellipsoid displacement plots of [Re(η^3^-C_7_H_7_)(η^5^-C_7_H_9_)(PTA)_2_]PF_6_ (**4PF**_**6**_), [Re(η^3^-C_7_H_7_)(η^5^-C_7_H_9_)(dppe)]PF_6_ (**5PF**_**6**_). Ellipsoids are drawn at 50% probability. The PF_6_^**–**^ anions are omitted for clarity. Coordination distances in Ångström (Å): **4PF**_**6**_: Re1–C1 = 2.307(4), Re1–C2 = 2.215(4), Re1–C3 = 2.270(4), Re1–C6 = 2.270(4), Re1–C7 = 2.268(4), Re1–C8 = 2.188(4), Re1–C9 = 2.284(4), Re1–C14 = 2.340(4), Re1–P1 = 2.4062(10), Re1–P2 = 2.4546(10); **5PF**_**6**_: Re1–C1 = 2.204(2), Re1–C2 = 2.356(2), Re1–C7 = 2.288(4), Re1–C8 = 2.300(3), Re1–C9 = 2.248(2), Re1–C10 = 2.259(2), Re1–C13 = 2.251(2), Re1–C14 = 2.231(4), Re1–P1 = 2.4730(6), Re1–P2 = 2.4629(6); [Re(CN-Ph)_6_]^+^: Re1–C1 = 2.0378(16).

Figure 3 shows the crystal structures of [Re(η^3^-C_7_H_7_)(η^5^-C_7_H_9_)(PTA)_2_]^+^ (**4**^**+**^) and [Re(η^3^-C_7_H_7_)(η^5^-C_7_H_9_)(dppe)_2_]^+^ (**5**^**+**^). The nature of [Re(η^3^-C_7_H_7_)(η^5^-C_7_H_9_)(CN-^*t*^Bu)_2_]^+^ (**6**^**+**^) was also confirmed by an X-ray structural determination but the data was of insufficient quality to be discussed here (Figure S35).

**Figure 4:**
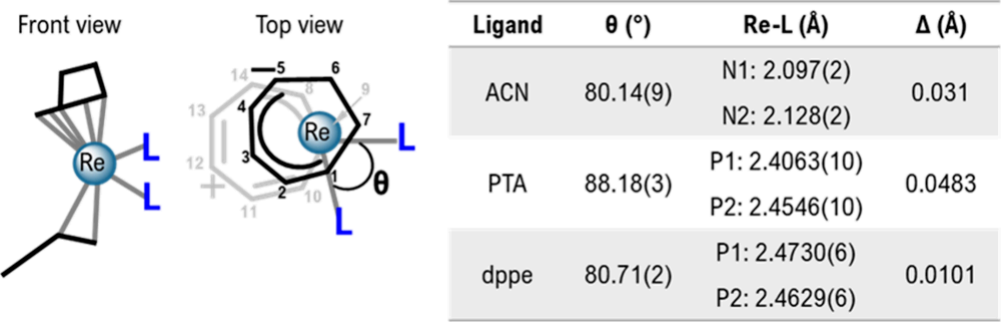
Schematic representation of the complexes [Re(η^3^-C_7_H_7_)(η^5^-C_7_H_9_)(L)_2_]^+^ and [Re(η^3^-C_7_H_7_)(η^5^-C_7_H_9_)(L)]^+^ from the front and top view (Left); table of Re–L bond distances (right).

Figure 4 shows a schematic representation of the complexes [Re(η^3^-C_7_H_7_)(η^5^-C_7_H_9_)(L)_2_]^+^ (L = acetonitrile, *tert-*butyl-CN, PTA, and dppe) from the front and the top view (the ligand [η^5^-C_7_H_9_]^−^ is at the top).

In all crystal structures, the η^3^-tropylium cation is bent. The C9 bound to rhenium is situated outside the plane of the remainder of the ligand, which is essentially planar in the structure of the starting complex **1**^**+**^. Moreover, the L–Re–L angle is close to 90° for the PTA compound (**4**) (88.18(3)) and slightly smaller for acetonitrile (**2**^**+**^) and dppe (**5**^**+**^) (80.14(9) and 80.71(2) respectively). In all the structures, one of the added ligands is located below the C7 methylene group of the cycloheptadienyl anion (N1 for **2**^**+**^, P1 for **4**^**+**^, and P2 for **5**^**+**^) while the other ligand lies below the C1 methine group of the cycloheptadienyl (N2 for **2**^**+**^, P2 for **4**^**+**^, and P1 for **5**^**+**^). Interestingly, the Re–L bond lengths within the same complex are slightly different. For all complexes, the Re–L bond located below C1 is longer than the one below C7 (see the table in Figure 3). The delta is smaller in the case of dppe ligand in **5**^+^. However, it should be considered that dppe is a bidentate ligand that brings some constraints to the structure.

**Figure 5:**
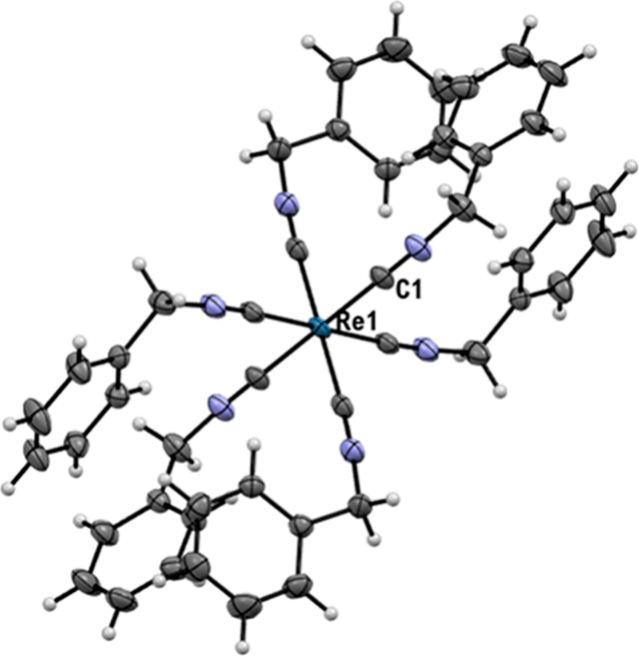
Ellipsoid displacement plots of [Re(CN-benzyl)_6_]^+^. Ellipsoids are drawn at 50% probability. The PF_6_^**–**^ anion is omitted for clarity. Coordination distances in Ångström (Å): Re1–C1 = 2.0378(16).

Finally, it was possible to obtain the X-ray quality crystals of [Re(CN-benzyl)_6_]^+^, which is one of the few structures of [Re(CN-R)_6_]^+^ reported up to now (Figure 5).^3^

### ^99^Tc complexes

**Scheme 5:**
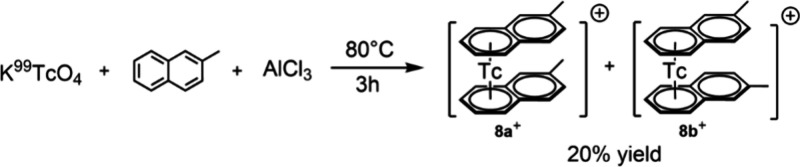
Synthesis of [^99^Tc(η^6^-C_11_H_10_)_2_]^+^ (**8**^**+**^)

As described in the introduction, the synthesis of [^99^Tc(η^6^-C_10_H_8_)_2_]^+^ has not been reported yet. Complexes of the type [^99^Tc(η^6^-C_6_R_6_)_2_]^+^ are conveniently accessible by the method of Benz et al. Reaction of ^99^TcO_4_^–^ with AlCl_3_ (15 equiv) as the Lewis acid and the desired arene as solvent (toluene, mesitylene, or tetraline) leads to the sandwich compounds in good yields (70–90%). With the exception of benzene, the reaction is more product-directed without elemental Zn as a reducing agent.^2^ The same procedure with molten naphthalene as successful in the case of rhenium did not yield [^99^Tc(η^6^-C_10_H_8_)_2_]^+^ for unknown reasons but reactions with the asymmetrical 2-methylnaphthalene (C_11_H_10_) showed more promise. Heating K^99^TcO_4_ with AlCl_3_ (15 equiv) in molten 2-methylnaphthalene at 80 °C for 3 h and subsequent aqueous extraction and anion exchange with LiOTf led to the formation of [^99^Tc(η^6^-C_11_H_10_)_2_]^+^ (**8**^**+**^) in 20% yield (Scheme 5). Compared to the synthesis of [Re(η^6^-arene)_2_]^+^, the ^99^Tc synthesis was faster (18 h for Re, 3 h for ^99^Tc). The faster reaction kinetics of technetium compared to its heavier congener rhenium has been observed in several reactions before.^2,14−16^

**Figure 6:**
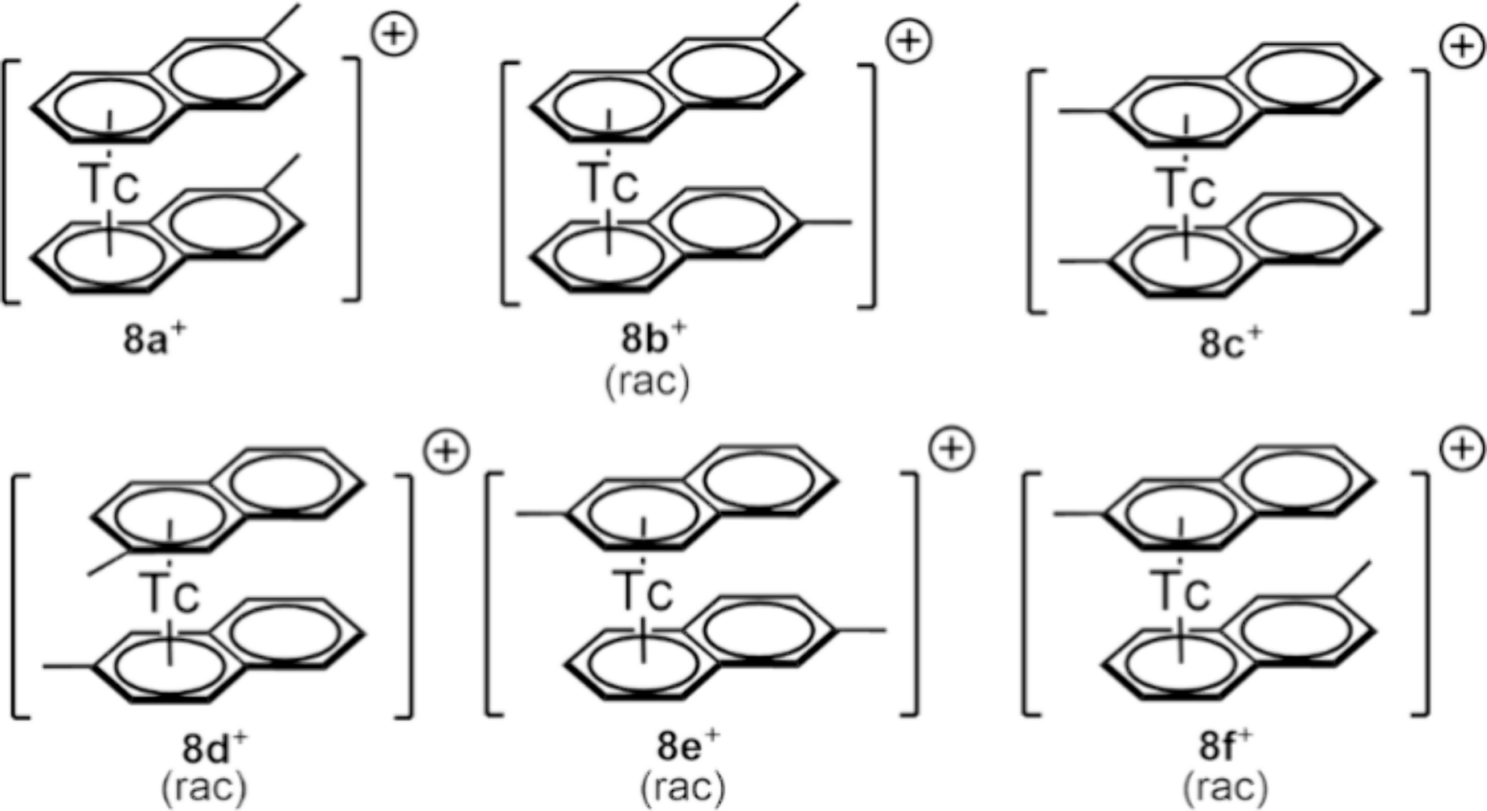
All possible Tc bis-η^6^-arene isomers with 2-methylnaphthalene as the ligand.

Since 2-methylnaphthalene divides the aromatic plane of the molecule in two different parts, up to 10 different isomers are possible, depending on how the arene coordinates the metal atom (i.e., planar chirality, Figure 6). There are six “symmetrical” isomers (**8a**^**+**^**–d**^+^), in which both naphthalene ligands are bound to the metal center through the same ring. They can coordinate either with the nonmethylated ring (**8a**^+^ and **8b**^+^ (racemic)) or with the methylated ring (**8c**^+^ and **8d**^+^ (racemic)). In addition, there are four different possibilities for asymmetrical binding arrangements (**8e**^+^ (racemic) and **8f**^+^ (racemic)).

The ^99^Tc NMR of **8**^**+**^ reveals two major signals at −1440 and −1442 ppm (Δ1/2 = 220 Hz), respectively (Figure S30). These values fall within the range typical for bis-arene complexes.^2^ The ^1^H NMR in deuterated acetone displays signals for two methyl groups at 2.44 and 2.41 ppm, along with 14 aromatic proton signals spanning from 7.3 to 5.8 ppm. Because of the closely matching chemical shifts, the exact count of distinct ^1^H signals could only be determined through ^1^H{^1^H} NMR (proton–proton decoupled ^1^H NMR) and ^1^H–COSY experiments. While two ^99^Tc signals were observed, there were only 14 different aromatic ^1^H signals and two singlets for the methyl groups. This rules the possibility of asymmetrical coordination of the naphthalene ligands (**8e**^+^ and **8f**^+^) out. In fact, in cases of asymmetrical coordination, each proton would have a distinct chemical environment. This implies that there should be 28 distinct aromatic protons and four methyl group signals. Therefore, we concluded that the naphthalene ligands coordinate symmetrically to the Tc-atom; either both with the methyl-substituted (**8c**^+^ and **8d**^+^) or with the unsubstituted ring (**8a**^+^ and **8b**^+^). 2D NMR spectra provided further evidence, confirming that the unsubstituted naphthalene ring coordinates to the ^99^Tc-atom (**8a**^+^ and **8b**^+^). See the Supporting Information for detailed description of the NMR spectra. To conclude, it was possible to determine the structure of the complex by using NMR experiments, without an X-ray structure analysis.

**Scheme 6:**
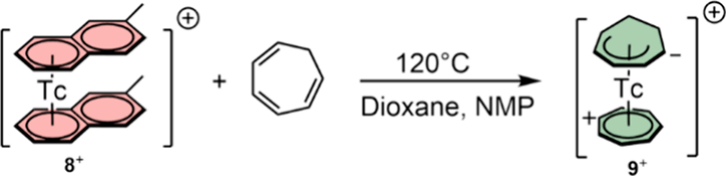
Synthesis of [^99^Tc(η^7^-C_7_H_7_)(η^5^-C_7_H_9_)]^+^ (**9**^**+**^)

To prepare the ^99^Tc homologue of **1**^**+**^, [^99^Tc(η^6^-C_11_H_10_)_2_]^+^ was reacted with cyclohepta-1,3,5-triene (C_7_H_8_) in refluxing dioxane and in the presence of 5 eq. of *N*-methyl-pyrrolidone (NMP) (Scheme 6), following the procedure reported for the synthesis of the rhenium analogue.^6^ HPLC analysis evidenced full conversion of **8**^**+**^ to [^99^Tc(η^7^-C_7_H_7_)(η^5^-C_7_H_9_)]^+^ (**9**^**+**^) after 2 h in 75% yield.

**Figure 7:**
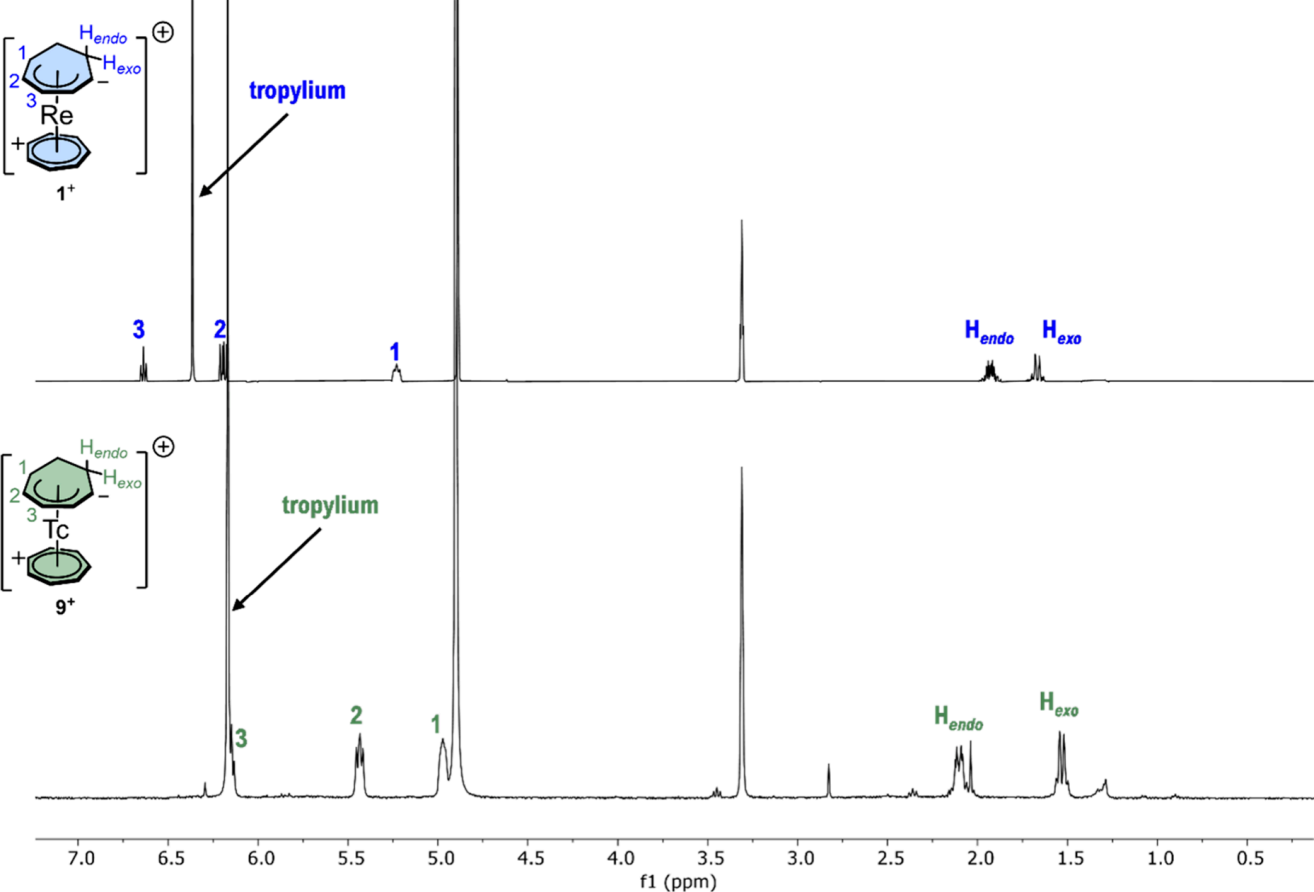
^1^H NMR spectra in methanol-*d*_4_ of [^99^Re(η^7^-C_7_H_7_)(η^5^-C_7_H_9_)]^+^ (**1**^**+**^, top) and [^99^Tc(η^7^-C_7_H_7_)(η^5^-C_7_H_9_)]^+^ (**9**^**+**^ bottom).

The ^1^H NMR spectrum is qualitatively similar to the one of the rhenium homologue (Figure 7), with a singlet at 6.17 ppm in the aromatic region, accounting for seven protons of the tropylium cation. The anion (C_7_H_9_)^−^ has three resonances of the η^5^-bound CH to the rhenium at 6.17 ppm (multiplet that accounts for one proton partially overlapped with the singlet of the tropylium cation), 5.44 ppm (multiplet accounting for two protons), and a multiplet at 4.97 ppm for the CH closer to the methylene groups. Finally, two signals are found for the protons *endo* and *exo* of the methylene groups at 2.11 and 1.54 ppm, respectively. All resonances were unambiguously assigned by means of 2D ^1^H–^1^H COSY and ^1^H–^13^C HSQC spectra (Figures S31, S32). The ^99^Tc NMR of **9**^+^ shows one signal at −1283 ppm (Δ1/2 = 1.6 kHz, Figure S33).

**Figure 8:**
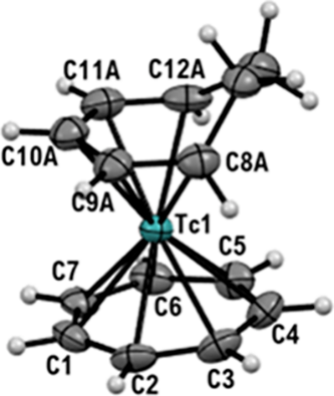
Ellipsoid displacement plots of [^99^Tc(η^6^-C_7_H_7_)(η^5^-C_7_H_9_)]CF_3_SO_3_ (**9**CF_3_SO_3_). Ellipsoids are drawn at 50% probability. The CF_3_SO_3_^–^ anion is omitted for clarity. Coordination distances in Ångström: Tc1–C1 = 2.224(4), Tc1–C2 = 2.252(4), Tc1–C3 = 2.244(4), Tc1–C4 = 2.242(4), Tc1–C5 = 2.244(4), Tc1–C6 = 2.249(4), Tc1–C7 = 2.247(4), Tc1–C8A = 2.319(14), Tc1–C9A = 2.253(13), Tc1–C10A = 2.245(14), Tc1–11A = 2.237(14), Tc1–C12A = 2.272(13).

The structure was confirmed by single crystals X-ray diffraction analysis (Figure 8).

**Figure 9:**
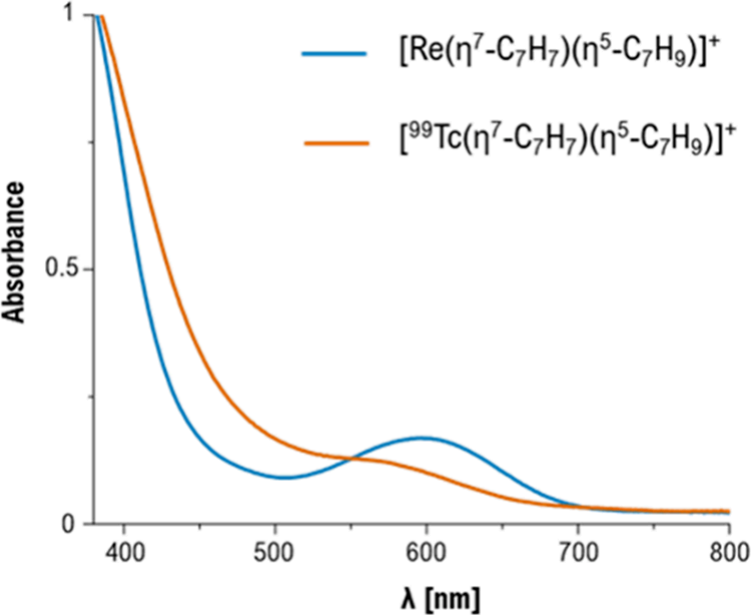
UV–vis spectra of [Re(η^6^-C_7_H_7_)(η^5^-C_7_H_9_)] (**1**^+^) (blue line) and [^99^Tc(η^6^-C_7_H_7_)(η^5^-C_7_H_9_)] (**9**^+^) (orange line) in methanol.

Electronic spectra of rhenium and technetium homologues are generally qualitatively comparable but shifted, and the resulting colors often rather similar. This is not the case for **1**^**+**^ and **9**^**+**^. Figure 9 shows the UV/vis spectra of **1**^**+**^ and **9**^**+**^ in methanol. The rhenium compound has a distinct absorption band with a maximum at 605 nm while the ^99^Tc homologue displays no specific feature in the visible region, just a very broad absorption band that covers the whole region.

Complex **1**^**+**^ was irradiated in the green region of the spectrum, resulting in the reactions described above. Due to the broad unresolved UV–vis spectrum in **9**^**+**^, the light-driven reactions with [^99^Tc(η^7^-C_7_H_7_)(η^5^-C_7_H_9_)]^+^ were performed with white light. Following the same procedure as for **1**^**+**^, **9**^**+**^ was dissolved in methanol-*d*_4_ in a glass vial and 10 μL of acetonitrile was added to the solution. Irradiation with white light did not lead to any changes in the ^1^H NMR spectra even after 4 d of irradiation. Thermal attempts were unsuccessful as well since [^99^Tc(η^7^-C_7_H_7_)(η^5^-C_7_H_9_)]^+^ was stable in acetonitrile for 17 h even at 65 °C.

Consequently, there is therefore a clear difference in the chemistry of Re(I) and Tc(I). This confirms similar findings as described for *fac*-[Re(CO)_3_]^+^ and *fac*-[Tc(CO)_3_]^+^ complexes with tris(1,2,3-triazolyl)phosphine oxides: while complexes with tripodal coordinated phosphine oxides are easily prepared with technetium, the reaction with rhenium leads to lower yield due to the formation of many side species.^17^

## Conclusions

The complex [Re(η^7^-C_7_H_7_)(η^5^-C_7_H_9_)]^+^ (**1**^**+**^) reacts under red light irradiation and an excess of an appropriate ligand (nitriles, phosphines and isocyanides) to form [Re(η^3^-C_7_H_7_)(η^5^-C_7_H_9_)(L)_2_]^+^. Two monodentate ligands or one bidentate ligand is added to the rhenium center with concerted ring slippage of the tropylium cation from η^7^ to η^3^. However, while they all display (η^3^-C_7_H_7_)^+^ coordination in the crystal structure, complexes with phosphines (**4**^**+**^, **5**^**+**^) and isocyanides (**6**^**+**^, **7**^**+**^) show a fluxional behavior of the tropylium cation in solution.

Only the nitrile ligands are labile and easily exchanged with solvent molecules (i.e., deuterated acetonitrile). Moreover, with nitriles the reaction is also reversible and [Re(η^3^-C_7_H_7_)(η^5^-C_7_H_9_)(CH_3_CN)_2_]^+^ converts to [Re(η^7^-C_7_H_7_)(η^5^-C_7_H_9_)]^+^ with blue light.

Albeit the technetium homologue of [Re(η^6^-C_10_H_8_)_2_]^+^ could not be prepared, the Fischer–Hafner reduction works well with 2-methylnaphthalene instead of naphthalene, obtaining [^99^Tc(η^6^-C_11_H_10_)_2_]^+^ (**8**^**+**^). In analogy to rhenium, the 2-methylnaphthalene in **8**^**+**^ did exchange with C_7_H_9_ and [^99^Tc(η^7^-C_7_H_7_)(η^5^-C_7_H_9_)]^+^ (**9**^**+**^) was obtained in good yields. This suggest that [^99^Tc(η^6^-C_11_H_10_)_2_]^+^ might serve as a precursor for Tc(I) chemistry, similarly as it happened with the Re analogue.

The conventional belief that reactions with rhenium and technetium homologues in low oxidation states are mirrors of each other is once more confuted. The electronic structures of **1**^**+**^ (Re) and **9**^**+**^ (^99^Tc) are significantly different, implying and entailing different reactivities. Whereas irradiation of **1**^**+**^ induces ring slippage and ligand coordination, **9**^**+**^ is completely unreactive under these conditions. We conclude that the analogy between Re and Tc might be in general but not “à priori” correct and require case-specific investigations.

## Experimental Part

### Materials and Techniques

This part is described in the Supporting Information.

### Selected Syntheses

#### General Procedure for Light-Driven Reaction

**1PF**_**6**_ (2 mg, 0.056 mmol) was dissolved in 550 μL of methanol-*d*_4_ in an NMR tube; 10 eq of the ligand was added, and the NMR tube was put into the light reactor. The reaction was followed by ^1^H NMR until full conversion of **1**^+^ to the product was observed.

#### [Re(η^3^-C_7_H_7_)(η^5^-C_7_H_9_)(NCCH_3_)_2_]PF_6_ (**2PF_6_**)

The compound was obtained after 52 h of irradiation with red light. Single crystals, suitable for X-ray diffraction analysis, were obtained by vapor diffusion from Et_2_O (antisolvent) into dichloromethane (solvent) overnight at 5 °C. ^1^H NMR (500 MHz, methanol-*d*_4_) δ (ppm): 6.27 (m, 1H, H2), 6.13 (m, 1H, H5), 5.69 (m, 1H, H4), 5.61 (m, 1H, H3), 5.51 (m, 1H, H8), 5.40 (m, 1H, H9), 4.78 (1H, H1 overlapped by H_2_O signal), 3.91 (m, 1H, C*H*_*endo*_), 3.75 (m, 1H, H11), 3.70 (m, 1H, H14), 2.85 (s, 3H, C*H*_3__2), 2.60 (s, 3H, C*H*_3__1), 2.37 (m, 1H, C*H*_e*ndo*_), 2.16, (1H, H13, overlapped by satellite signal of free ACN) 2.09 (m, 1H, C*H*_exo_), 2.02 (2H, H12 + C*H*_exo_ overlapped by signal of free ACN), 1.43 (m, 1H, H10). ^13^C NMR (121 MHz, methanol-*d*_4_) δ (ppm): 138.68 (C5), 135.53 (C2), 132.03 (CN), 124.81 (CN), 123.09 (C4), 121.08 (C3), 111.63 (C9), 99.85 (C10), 95.54 (C8), 78.84 (C12, C14), 70.35 (C1), 67.12 (C11, C13), 45.66 (CH_2_), 31.28 (CH_2_), 3.77 (CH_3__1), 3.28 (CH_3__2). HRMS (ESI^+^) *m*/*z* calcd. for C_18_H_22_N_2_Re [M]^+^: 453.13350, found: 453.13364.

#### [Re(η^3^-C_7_H_7_)(η^5^-C_7_H_9_)(PTA)_2_]PF_6_ (**4PF_6_**)

The compound is obtained after 2 d of irradiation with red light. Single crystals, suitable for X-ray diffraction analysis, were formed during the reaction.

Note: at 298 K, the resonances of the tropylium cation are missing due to its fluxional behavior. NMR (500 MHz, methanol-*d*_4_) δ (ppm): δ 5.81 (m, 1H, H3), 5.52 (m, 1H, H4), 4.78 (m, *J* = 13.4 Hz, 6H, NC*H*_2_N), 4.63 (d, *J* = 14.0 Hz, 6H, NC*H*_2_N), 4.49 (m, 6H, NC*H*_2_P), 4.25 (m, 6H, NC*H*_2_P), 4.03 (d, *J* = 4.6 Hz, 1H, H5), 3.62 (d, *J* = 8.0 Hz, 1H, H1), 2.12 (m, 4H, 2CH_2_), 1.37 (t, *J* = 7.6 Hz, 1H, H4). ^13^C NMR (125 MHz, methanol-*d*_4_, from ^1^H–^13^C-HSQC) δ (ppm): 118 (C3), 104 (C4), 86 (C2), 71 (C5), 60 (C1), 34 (CH_2_), 33 (CH_2_). ^31^P NMR (162 MHz, methanol-*d*_*4*_) δ – 87.47 (d, *J* = 26.6 Hz), – 106.55 (d, *J* = 26.7 Hz). HRMS (ESI^+^) *m*/*z* calcd. for C_26_H_40_N_6_P_2_Re [M]^+^: 685.23417, found: 685.23462.

#### [Re(η^3^-C_7_H_7_)(η^5^-C_7_H_9_)(CN-^*t*^Bu)_2_]PF_6_ (**6PF_6_**)

The compound is obtained after 48 h of irradiation with red light.

Note: at 298 K, the resonances of the tropylium cation are missing due to its fluxional behavior.

NMR (500 MHz, methanol-*d*_4_, 193 K) δ (ppm): 6.17 (m, 2H, CH tropylium cation), 5.89 (m, 1H, H3), 5.67 (m, Hz, 2H, CH tropylium cation), 5.47 (m, 1H, H2), 4.65 (br s, 1H, CH tropylium cation), 4.25 (m, 1H, H5), 3.97 (br s, 1H, CH tropylium cation), 3.87 (t, 1H, H1), 2.48 (m, 1H, C*H*_e*ndo*_), 2.22 (m, 1H, C*H*_e*ndo*_), 2.06 (m, 2H, C*H*_e*xo*_), 1.70 (m, 11H, C*H*_e*ndo*_*+* H4 + *tert*-butyl), 1.53 (s, 9H, *tert*-butyl). ^13^C NMR (125 MHz, methanol-*d*_4_) δ (ppm): 116.3 (C3), 110.0 (C4), 88.4 (C2), 80.1 (C5), 71.1 (C1), 61.2 (C quaternary *tert*-butyl), 60.3 (C quaternary *tert*-butyl), 36.8 (CH_2_), 32.0 (CH_2_), 30.8 (CH_3_*tert*-butyl). HRMS (ESI^+^) *m*/*z* calcd. for C_24_H_34_N_2_Re [M]^+^: 537.22740, found: 537.22726.

*Caution: ^99^Tc is a weak beta emitter. All experiments have to be carried out in licensed and appropriately shielded laboratories for low-level radioactive materials.*

#### [^99^Tc(η^6^-C_11_H_10_)_2_]PF_6_ (**8aPF_6_** and **8bPF_6_**)

In a 25 mL Schlenk flask, 2-methylnaphthalene (3.01 g, 21.1 mmol), K^99^TcO_4_ (10.6 mg, 0.052 mmol), and AlCl_3_ (0.107 g, 0.80 mmol) were heated at 80 °C for 3 h. To the dark red/black suspension, heptane (3 mL) was added, and the suspension was filtered through a glass filter frit. This process was repeated four times, before H_2_O (6 mL) was added to the reaction mixture. The aq. extract was filtered through the same filter frit and the water extraction was repeated (2 × 3 mL). Additional H_2_O (6 mL) was added to the reaction flask and stirred for 16 h, before the suspension was again filtered. The combined aqueous solutions were washed with CH_2_Cl_2_ (6 mL), before NH_4_PF_6_ (24.5 mg, 0.15 mmol) was added. The aq. solution was extracted with CH_2_Cl_2_ (8 and 4 mL) and the bright yellow organic phase was concentrated with N_2_ to give **8PF**_**6**_ (4.5 mg, 0.01 mmol, 20%) as a reddish solid. Notes: the product was obtained as a set of the diastereomers **8a**^+^ and **8b**^+^. Instead of NH_4_PF_6_ the equimolar amounts of LiOTf can be added to obtain **8OTf**. Analytical data of **8aPF**_**6**_: ^1^H NMR (500 MHz, acetone-*d*_6_) δ (ppm): 7.32 (dd, *J* = 8.9 Hz, 1.5 Hz, 2H, H3), 7.02 (d, *J* = 8.9 Hz, 2H, H4), 6.72 (s, 2H, H1), 6.41 (d, *J* = 5.6 Hz, 2H, H8), 6.26 (d, *J* = 5.6 Hz, 2H, H5), 5.89 (t, *J* = 5.6 Hz, 2H, H6), 5.79 (t, *J* = 5.7 Hz, 2H, H7), 2.44 (d, *J* = 0.9 Hz, 2H, CH_3_). ^13^C NMR (151 MHz, acetone-*d*_6_) δ (ppm): 141.4 (2C, C2), 133.7 (2C, C3), 130.0 (2C, C4), 125.9 (2C, C1), 22.1 (2C, CH_3_). ^99^Tc NMR (90 MHz, acetone-*d*_6_) δ (ppm): −1440 (Δ1/2 = 220 Hz) or −1442 (Δ1/2 = 220 Hz). HPLC: R*t* = 17.3 min (β^*–*^). Analytical data of **8bPF**_**6**_: ^1^H NMR (500 MHz, acetone-*d*_6_) δ (ppm): 7.32 (dd, *J* = 8.9 Hz, 1.5 Hz, 2H, H3′), 6.96 (d, *J* = 8.9 Hz, 2H, H4’), 6.79 (s, 2H, H1’), 6.34 (m, 2H, H8’), 6.33 (m, 2H, H5′), 5.86–5.81 (m, 4H, H6’ and H7’), 2.40 (d, *J* = 0.9 Hz, 6H, CH_3_’). ^13^C NMR (151 MHz, acetone-*d*_6_) δ (ppm): 141.2 (2C, C2’), 133.0 (2C, C3′), 129.5 (2C, C4’), 126.3 (2C, C1’), 22.0 (2C, CH3′). ^99^Tc NMR (90 MHz, acetone-*d*_6_) δ (ppm): −1440 (Δ1/2 = 220 Hz) or −1442 (Δ1/2 = 220 Hz). HPLC: R*t* = 17.3 min (β^–^).

#### Synthesis of [^99^Tc(η^7^-C_7_H_7_)(η^5^-C_7_H_9_)]PF_6_ (**9PF_6_**)

**8OTf** (6.6 mg, 0.015 mmol) was partially dissolved in dioxane (0.5 mL) and mixed with cyclohepta-1,3,5-triene (0.5 mL, 5.55 mmol, in excess) and NMP (10.0 μL, 0.106 mmol, in excess) in a MW vial, which was heated to 110 °C for 2 h under nitrogen. The solvent was removed by a stream of N_2_, and the residue was washed with Et_2_O (3 × 3 mL), affording **9OTf** (3.93 mg, 75%) as an orange solid. Single crystals, suitable for X-ray diffraction analysis, were obtained from **9OTf** by vapor diffusion from Et_2_O (antisolvent) into methanol (solvent) overnight at 5 °C. ^1^H NMR (400 MHz, methanol-*d*_4_) δ (ppm): 6.17 (m, 8H, CH tropylium + H3), 5.44 (m, 2H, H2), 4.98 (m, 2H, H1), 2.10 (m, 2H, H_*endo*_), 1.54 (m, 2H, H_*exo*_). ^13^C NMR (methanol-*d*_4_, from HSQC) δ (ppm): 98 (C2), 96 (C3), 93 (C tropylium), 87 (C1), 37 (CH_2_). ^99^Tc NMR (methanol-*d*_4_, 90 MHz) δ (ppm): −1283 (Δ1/2 = 1.6 kHz). HPLC: R*t* = 13.3 min (β^*–*^).

## Supporting Information Available

Detailed NMR characterization of **8**^**+**^; material and methods, syntheses and characterization of **3**^+^, **5**^+^, and **7**^+^; picture and UV emission spectra of the homemade photoreactor; NMR spectra of **2**^+^–**9**^+^, kinetic data of **2**^+^, crystallographic details of **2**^+^, **4**^**+**^, **5**^+^, **9**^+^, and [Re(CN-benzyl)_6_]^+^ (PDF)

## Abbreviations:

dppe: (ethane-1,2-diyl)bis(diphenylphosphane
ESI: electrospray ionization
Et_2_O: diethyl ether
HR: high-resolution
MS: mass spectrometry
NMP: *N*-methyl-2-pyrrolidone
NMR: nuclear magnetic resonance
OTf: triflate
PTA: 1,3,5-triaza-7-phosphoadamantane
*^t^*Bu: *tert*-butyl
UHPLC: ultrahigh-performance liquid chromatography
