# Supplementary material for: Light-Driven Ring Slippage in [Re(η7-C7H7)(η5-C7H9)]+ and the Inertness of Its Technetium Homologue
Source: Inorg Chem. 2024 Jan 22;63(5):2701–8. doi: 10.1021/acs.inorgchem.3c04052 (PMC10848200; doi:10.1021/acs.inorgchem.3c04052)
Supplement: Supplementary file 1 — ic3c04052_si_001.pdf [file ic3c04052_si_001.pdf]

Light driven ring slippage in [Re(η^7-C_7H_7)(η^5-C_7H_9)]$^+$ and the inertness of its Technetium homologue

Federica Battistin*, Robin Bolliger, Manuel Luca Besmer, Thomas Fox, Olivier Blacque, Henrik Braband and Roger Alberto

Department of Chemistry, University of Zurich, Winterthurerstrasse 190, 8057 Zurich, Switzerland. E-mail: federica.battistin@univ-brest.fr

Table of contents:

NMR characterization of $[^{99}Tc(\eta^6\text{-}C_{11}H_{10})_2]^+$ (8^+) 3
Materials and Techniques 6
Syntheses 7
Homemade photoreactor 8
NMR spectra 10
Kinetics measurements 24
X-ray crystallography 25
HR-ESI-MS Spectra 30
References: 36

NMR characterization of [^{99}Tc(η^6-$C_{11}H_{10}$)$_2$]$^+$ (8$^+$)

As both coordination modes consist of a set of diastereomers, all proton signals (2 x 7 aromatic protons and 2 x methyl groups) are accounted for. The ^{1}H-^{1}H COSY spectrum shows four distinct coupling systems form the aromatic protons (two coupling systems for one molecule or set of enantiomers respectively). The two low field shifted coupling systems consist of six protons (respectively three protons for one coupling system; in Figure S1., green box), meaning that they belong to the methylsubstituted ring system. In fact, the two high field shifted coupling systems consist of eight protons (respectively four protons for one coupling system; in Figure S1 red box), which belongs to the unsubstituted ring. Since many reports have shown that coordination of an arene to a ^{99}Tc or Re core leads to an high field shift of its ^{1}H signals,[1–4] the unsubstituted naphthalene ring coordinates the ^{99}Tc-atom (**8a**$^+$ and **8b**$^+$). Similar stereochemical results were obtained for the preparations of [M(η^6-1,4-dimethylnaphthalene)$_2$] (M = Mo, Cr). The reactions yield the symmetrical isomer exclusively, with coordination of the metal to the unsubstituted ring.[5,6]

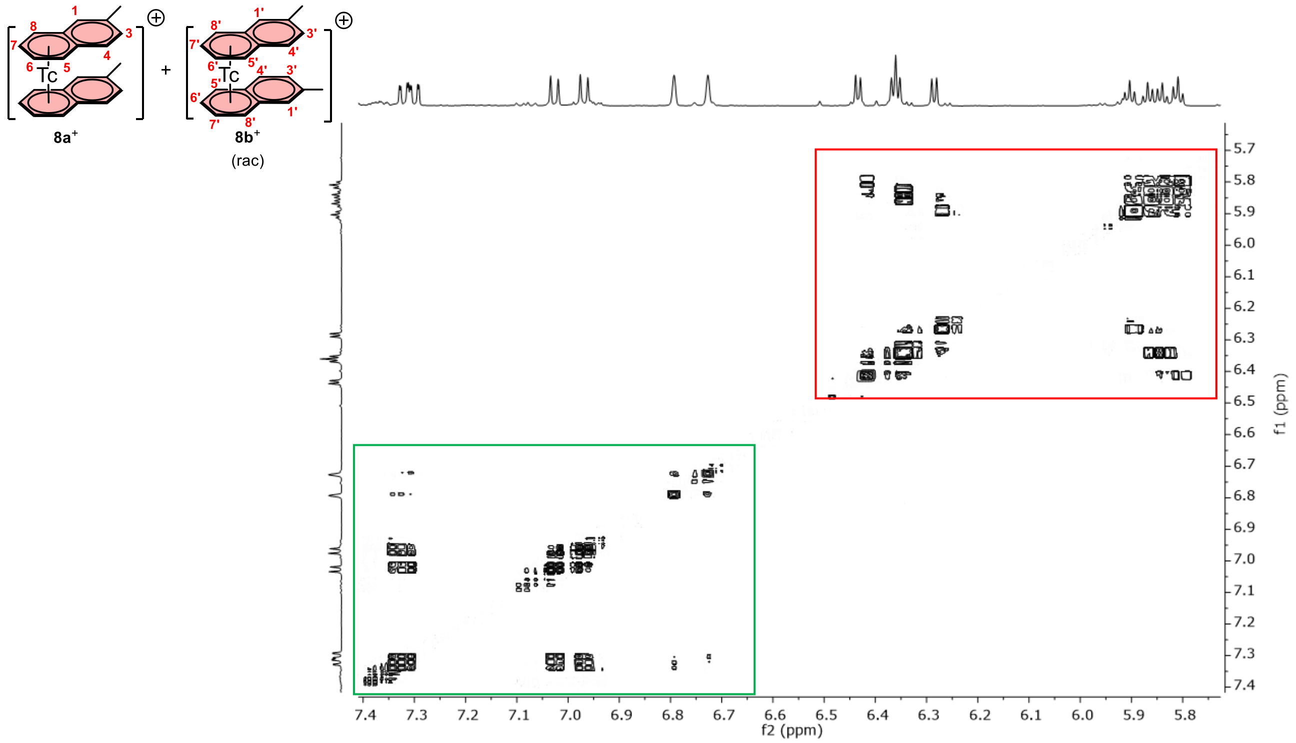

Figure S1. ^{1}H-^{1}H COSY spectrum in in acetone-d_6. of [^{99}Tc(η^6-$C_{11}H_{10}$)$_2$]$^+$ (**8**$^+$). The two boxes represent the coupling systems of the aromatic protons. The green box includes two coupling systems with three protons each. The red box contains two coupling systems with four protons each.

Due to a small coupling between the protons at the naphthalene positions 4 and 5 (Figure S2), the ^{1}H-^{1}H COSY spectrum allowed to connect the coupling systems of the methyl-substituted and unsubstituted ring system, giving the complete ^{1}H signal set for one diastereomer. However, ^{1}H-^{1}H COSY spectrum could not indicate which set of ^{1}H signals belonged to which diastereomer. In addition, NOESY experiments were also inconclusive.

The correct assignment was achieved by comparing the chemical shifts of the protons belonging to the coordinating ring system (H5, H8 vs H5', H8' and H6, H7 vs H6', H7', respectively). While protons H5 and H8 have very distinct chemical shifts, those of H5' and H8' are very similar. This is even clearer when comparing H6 and H7 with H6' and H7', which overlap. This means that, on the one side, the chemical environment of protons H5 and H8 (which are H6 and H7) must be distinctly

different, while, on the other side, the chemical environment for H5' and H8' (H6' and H7') must be very similar. The proximity to the two methyl groups is the only possible difference in chemical environment for these protons. Examination of the protons at naphthalene positions 5 and 8 of **8a**$^+$ shows that position 8 is in proximity to both methyl-groups, while 5 is further away from both methyl-groups. In the case of **8b**$^+$, the proton at position 8 is in close to the methyl-group located on the same naphthalene ligand and further away from the CH_3-group of the opposing naphthalene ligand. On the other hand, the proton at position 5 is close to the methyl-group on the opposing naphthalene ligand, but further away from the CH_3-group located on the same naphthalene ligand. Thus, the chemical shifts of protons at positions 5 and 8 of **8b**$^+$ should be more similar than the chemical shifts of the corresponding signals of **8a**$^+$. The same is true for positions 6 and 7, thus, the correct set of ^{1}H signals can be assigned to the correct diastereomer.

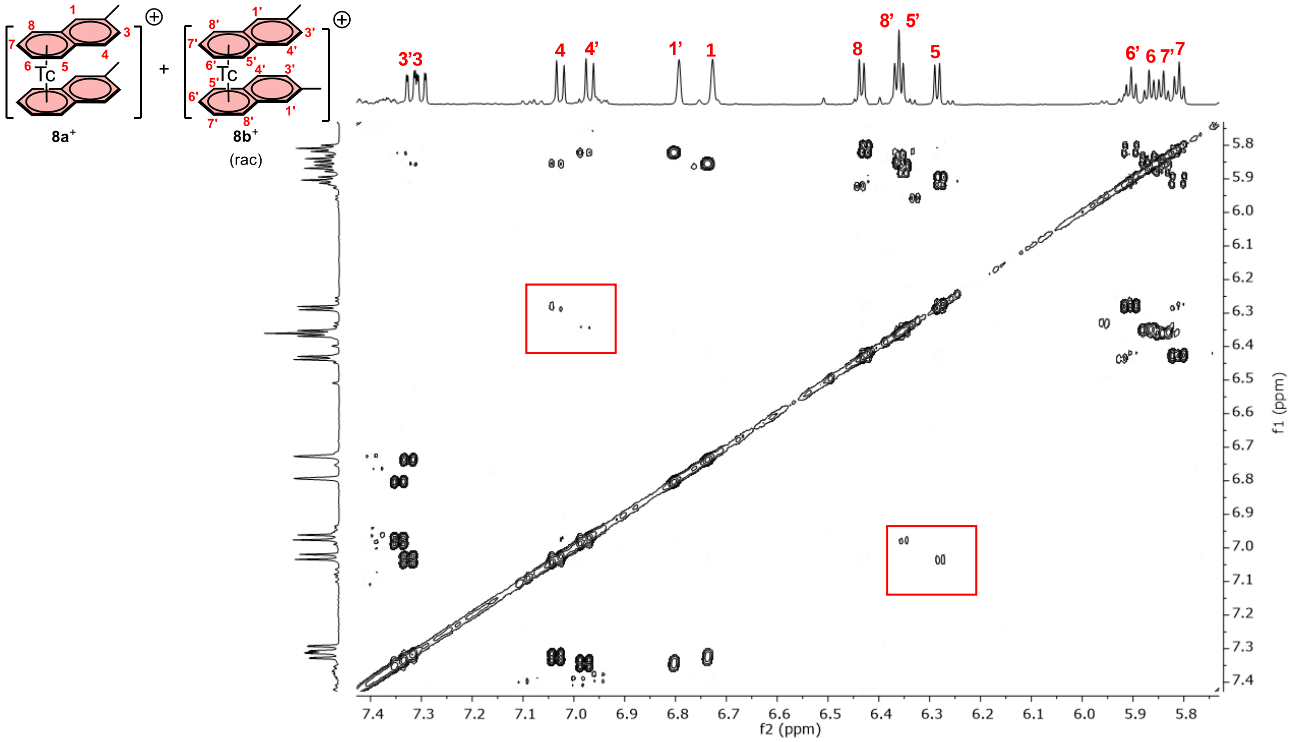

Figure S2. ^{1}H-^{1}H-COSY spectrum in acetone-d_6 of $[^{99}Tc(\eta^6-C_{11}H_{10})_2]^+$ (**8**$^+$) with the assignment of all ^{1}H signals to the correct diastereomers **8a**$^+$ and **8b**$^+$. The red boxes highlight the coupling between the protons at the naphthalene-positions.

For the ^{13}C NMR, only the signals of the non-coordinated ring could be assigned to the corresponding structure. The ^{13}C signals of the coordinated ring could not be resolved and only appear as a very broad signal (δ = 80 – 85 ppm). This is originated from coupling with the ^{99}Tc nucleus (S = 9/2) and was also found for other $[^{99}Tc(\eta^6-C_6R_6)_2]^+$ systems:[2] the scalar coupling propagates to the ^{13}C-cores through the π-bonds due to large T1 relaxation times of the ^{99}Tc nucleus. In the case of long pulse ^{13}C NMR experiments on the bis-η^6-toluene complex **8**$^+$, a scalar coupling of $J \approx 34$ Hz was found for the aromatic *ipso*-carbon in the toluene ligand (Figure S3).

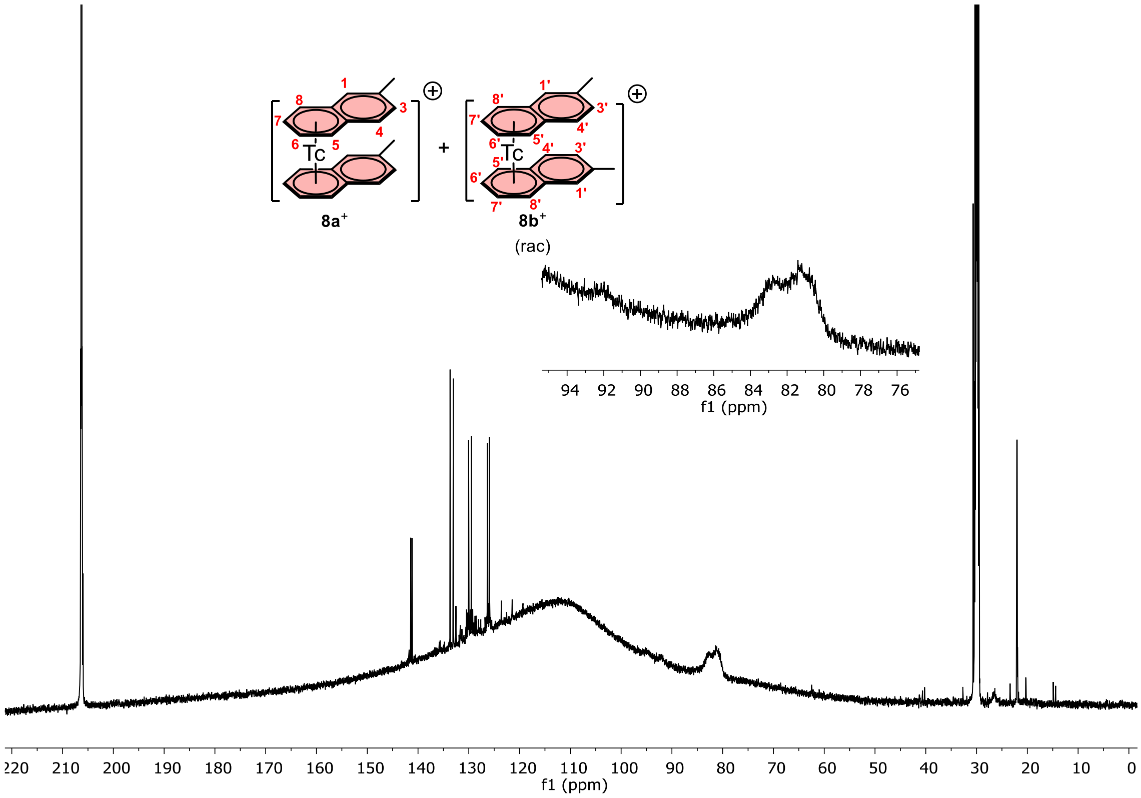

Figure S3. ^{13}C NMR in acetone-d_6 of [^{99}Tc(η^6-$C_{11}H_{10}$)$_2$]$^+$ (**8**$^+$). The broadness of the ^{13}C signals (at around δ = 110 ppm and 80 ppm, respectively) originates from scalar coupling with the ^{99}Tc-core through the π-bonds.

Materials and Techniques

Unless otherwise stated, all chemicals were of reagent grade or higher, obtained from commercial sources and used without further purification. [Re(η^6-$C_{10}H_8$)$_2$]$^+$ and [Re(η^7-C_7H_7)(η^5-C_7H_9)]$^+$ were synthesize according to literature procedures.[7,8] Solvents for reactions were of p.a. grade or distilled prior to their use; H_2O was bi-distilled. Deuterated NMR-solvents were purchased from Armar Chemicals or Cambridge Isotope Laboratories, Inc. (UK). (NH_4)[$^{99}TcO_4$] was purchased from Oak Ridge and treated with H_2O_2 prior to reactions for re-oxidation of black, surface TcO_2.[9] Caution: ^{99}Tc is a weak β-emitter. Reactions were carried out using standard Schlenk techniques in oven-dried (150 °C) glass equipment and monitored for completion by analyzing a small sample (after suitable workup) by, UPLC-ESI-MS. Evaporation of the solvents in vacuo was done with the rotary evaporator.

<u>^{1}H- and ^{13}C-NMR spectra:</u> BrukerAV2-400 (400 MHz) or Bruker DRX-500 (500 MHz); in deuterated solvents at 300 K when not specified; chemical shifts (δ) in ppm relative to residual solvent resonances (acetone-d_4 ^{1}H: δ 2.05, ^{13}C: δ 29.84; methanol-d_4 ^{1}H: δ 3.31, ^{13}C: δ 49.00); coupling constants (J) in Hz. Chemical shifts of ^{13}C spectra are given with one digit after the comma if they are from a ^{13}C spectrum, with none digit when are from HSQC spectra. The values given for the ^{99}Tc chemical shifts are referenced to pertechnetate.

<u>HR-ESI-MS:</u> QExactive (Thermo Fisher Scientific, Bremen, Germany) equipped with a heated ESI source connected to a Dionex Ultimate 3000 UPLC system. Samples dissolved in MeOH, at ca. 50 µg mL−1; injection of 1 µL on-flow with an XRS auto-sampler (CTC, Zwingen, Switzerland)(mobile phase: MeOH + 0.1% HCOOH or CH_3CN/H_2O (2:8) + 0.1% HCOOH; flow rate 120 µL mL−1); ion source parameters: spray voltage 3.0 kV, capillary temperature 280 °C, sheath gas 30 L min−1, s-lens RF level 55.0; aux gas temperature 250 °C; full scan MS in alternating (+)/(−)-ESI mode; mass ranges 80–1200, 133–2000, or 200–3000 amu; resolution (full width half-maximum) 70000; automatic gain control(AGC) target 3.00 106; maximum allowed ion transfer time(IT) 30 ms; mass calibration < 2 ppm accuracy for m/z 130.06619 – 1621.96509 in (+)-ESI with Pierce® ESI calibration solutions (Thermo Fisher Scientific, Rockford, USA); lock masses: ubiquitous erucamide (338.34174 m/z, (+)-ESI).

<u>UPLC-ESI-MS</u>: Waters Acquity UPLC System coupled to a Bruker Daltonics HCTTMESI-MS, using an Acquity UPLC BEH C18 1.7 µm (2.1 x 50 mm) column. UPLC solvents were formic acid (0.1% in millipore water) (solvent A) and acetonitrile UPLC grade (solvent B). Applied UPLC gradient: 0–0.5 min: 95% A, 5% B; 0.5–4.0 min: linear gradient from 95% A, 5% B to 0% A, 100% B; 4.0–5.0 min: 0% A, 100% B. The flow rate was 0.6 mL min^{-1}. Detection was performed at 250 and 480 nm (DAD).

<u>Home made light reactor:</u> The light reactor consists of a box with a Mi LED Smart Bulb (White and Color). The distance between the lamp and the NMR tube or vial is of 8 cm. The color and the power of the light was chosen with the Xiaomi app. The box was kept closed during the reaction. See Figure S4 for a picture of the system. The emission spectra of each light were measured using ThorLabs Compact Spectrometer (Figure S5).

<u>Radio HPLC:</u> Analytical HPLC of ^{99}Tc complexes on a Merck Hitachi LaChrom L7100 pump coupled to a Merck Hitachi LaChrom L7200 tunable UV detector and a Berthold LB513 radiodetector equipped with YG cells. HPLC solvents were 0.1 % trifluoroacetic acid (solvent A) and acetonitrile (solvent B). Applied HPLC gradient: 0-3min: 95% A (5% B); 3-3.1 min: 95% A (5% B) to 75% A (25% B); 3.1-9 min: 75% A (25% B); 9-9.1 min: 75% A (25% B) to 66% A (34% B); 9.1-20 min:

66% A (34% B) to 0% A (100% B); 20-25 min: 0% A (100% B); 25-25.1 min: 0% A (100% B) to 95% A (5%B); 25.1-30 min: 95% A (5% B). The flow rate was 0.5 ml min-1. UV-vis detection was performed at 250 nm.

Syntheses

General procedure for light reaction: 1PF$_6$ (2 mg, 0.056 mmol) was dissolved in 550 µL of methanol-*d$_4$* in an NMR tube; 10 eq of the ligand were added and the NMR tube was put into the light reactor. The reaction was followed by ^{1}H NMR until full conversion of **1**$^+$ to the product was observed.

[Re(η^3-C$_7$H$_7$)(η^5-C$_7$H$_9$)(NCCH$_2$Ph)$_2$]PF$_6$ (3PF$_6$). The compound is obtained after 140 h of irradiation with red light. ^{1}H NMR (500 MHz, methanol-*d$_4$*) δ (ppm): 7.34 (m, Ph overlapped with Ph signals of free benzyl nitrile), 6.24 (m, 1H, H2), 6.11 (m, 1H, H5), 5.68 (m, 1H, H4), 5.59 (m, 1H, H3), 5.47 (m, 1H, H8), 5.36 (m, 1H, H9), 4.78 (overlapped by H$_2$O signal, 1H, H1), 4.59 (s, 3H, H12+CH$_2$ benzyl), 4.35 (s, 2H, CH$_2$ benzyl), 3.91 (1H, CH$_{endo}$ overlapped by CH$_2$ benzyl free benzyl nitrile), 3.71 (m, 1H, H11), 3.59 (m, 1H, H14), 2.26 (m, 1H, C*H*$_{endo}$), 2.07 (m, 1H, H13), 1.93, (m, 2H, CH$_{exo}$), 1.42 (m, 1H, H10). ^{13}C NMR (from ^{1}H-^{13}C HSQC, methanol-*d$_4$*) δ (ppm): 138 (C5), 135 (C2), 123 (C3), 122 (C4), 111 (C9), 100 (C10), 95 (C8), 79 (C14), 70 (C12) 68 (C11), 52 (C13), 45 (CH$_2$), 29, (CH$_2$), 26 (CH$_2$ benzyl), 25 (CH$_2$ benzyl). HRMS (ESI$^+$) m/z calcd. for C$_{30}$H$_{30}$N$_2$Re [M]$^+$: 605.19610, found: 605.19632.

[Re(η^3-C$_7$H$_7$)(η^5-C$_7$H$_9$)(dppe)$_2$]PF$_6$ (5PF$_6$). The compound is obtained after 15 days of irradiation with red light. Single crystals, suitable for X-ray diffraction analysis were formed during the reaction. Note: Due to the low solubility of the crystals in most common solvents (methanol, acetone, DMSO, acetonitrile), it could be only partially characterized by NMR. Moreover, as for **4**$^+$, at 298 K the resonances of tropylium cation are missing due to its fluxional behavior.
NMR (400 MHz, acetone-*d$_6$*) δ (ppm): ^{1}H NMR (400 MHz, acetone-*d*$_6$) δ 7.30 (m, Ph), 6.16 (t, *J* = 6.6 Hz, 1H, H3), 5.06 (t, *J* = 10.0 Hz, 1H, H4), 4.43 (s, 1H, H5), 2.98 (m, 4H, H1 + tropylium), 1.75 (m, 2H, C*H*$_2$), 1.33 (m, 2H, C*H*$_2$), 0.70 (m, 1H, H4).^{31}P NMR (162 MHz, acetone-*d$_6$*) δ (ppm): 19.52 (br s), 16.06 (br s). HRMS (ESI$^+$) m/z calcd. for C$_{40}$H$_{40}$P$_2$Re [M]$^+$: 769.21573, found: 769.21565.

[Re(η^3-C$_7$H$_7$)(η^5-C$_7$H$_9$)(CN-benzyl)$_2$]PF$_6$ (7PF$_6$). The compound is obtained after 72 h of irradiation with red light.
Note: at 298 K the resonances of tropylium cation are missing due to its fluxional behavior. NMR (500 MHz, methanol-*d$_4$*) δ (ppm): 7.43 (m, 10H, 2Ph rings) 5.87 (m, 1H, H3), 5.43 (m, 3H, H2 + CH$_2$ CN-benzyl), 5.14 (s, 2H, CH$_2$ CN-benzyl) 4.24 (m, 1H, H5), 3.85 (t, 1H, H1), 2.33 (m, 1H, C*H*$_{endo}$), 1.93 (2H, C*H*$_{endo}$ + C*H*$_{exo}$), 1.67 (m, 2H, C*H*$_{exo}$ + H4). ^{13}C NMR (125 MHz, methanol-*d$_4$*) δ (ppm): 129.3 (Ph), 127.9 (Ph), 116.0 (C3), 110.2 (C4), 88.2 (C2), 80.9 (C5), 71.1 (C1), 46.1 (CH$_2$ CN-benzyl) 35.9 (CH$_2$), 32.0 (CH$_2$). HRMS (ESI$^+$) m/z calcd. For C$_{24}$H$_{34}$N$_2$Re [M]$^+$: 537.22740, found: 537.22711. HRMS (ESI$^+$) m/z calcd. for C$_{30}$H$_{30}$N$_2$Re [M]$^+$: 605.19610, found: 605.19632.

Homemade photoreactor

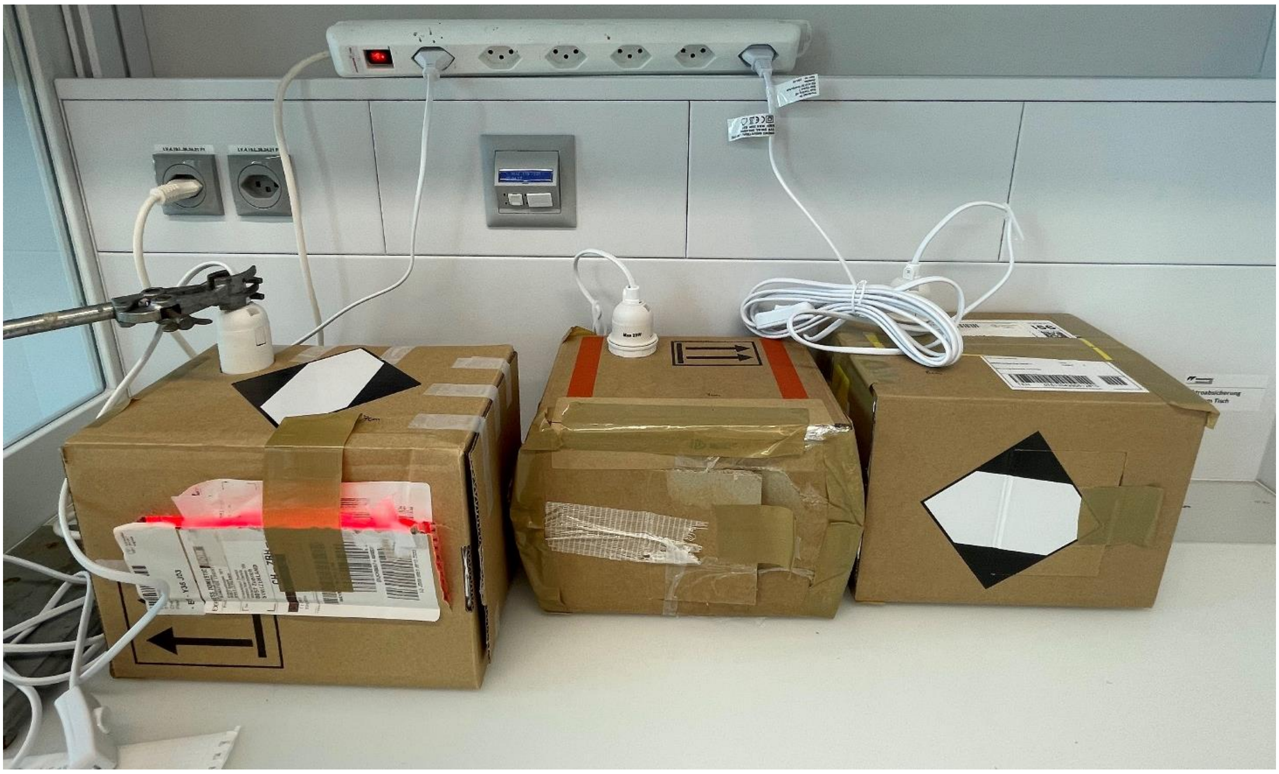

Figure S4. Homemade photoreactors.

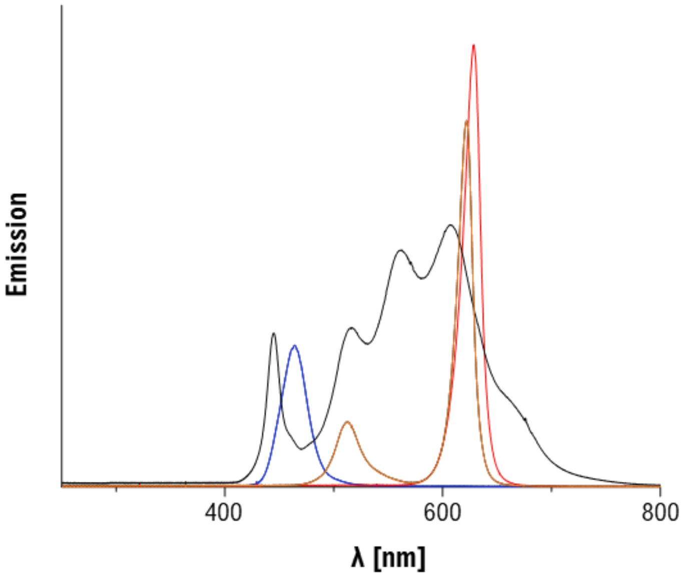

Color	Emission (nm)
Blue	465
Orange	514, 621
Red	629
White	444, 515, 562, 608

Figure S5. Emission spectra of each lamp: blue lamp (blue line), orange lamp (orange line), red lamp (red line), white lamp (black line). Wavelengths are listed on the table.

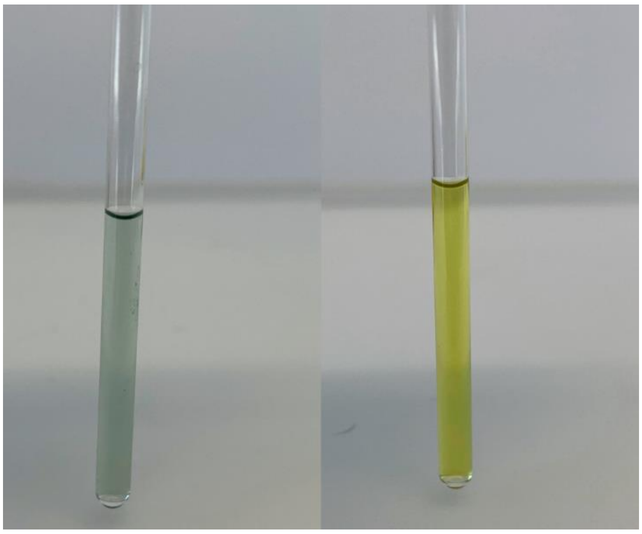

Figure S6. $[Re(\eta^7-C_7H_7)(\eta^5-C_7H_9)]^+$ (**2**$^+$) dissolved in methanol-d_4 with 20µL of acetonitrile at t = 0 (left) and t = 52 h of irradiation with red light.

NMR spectra

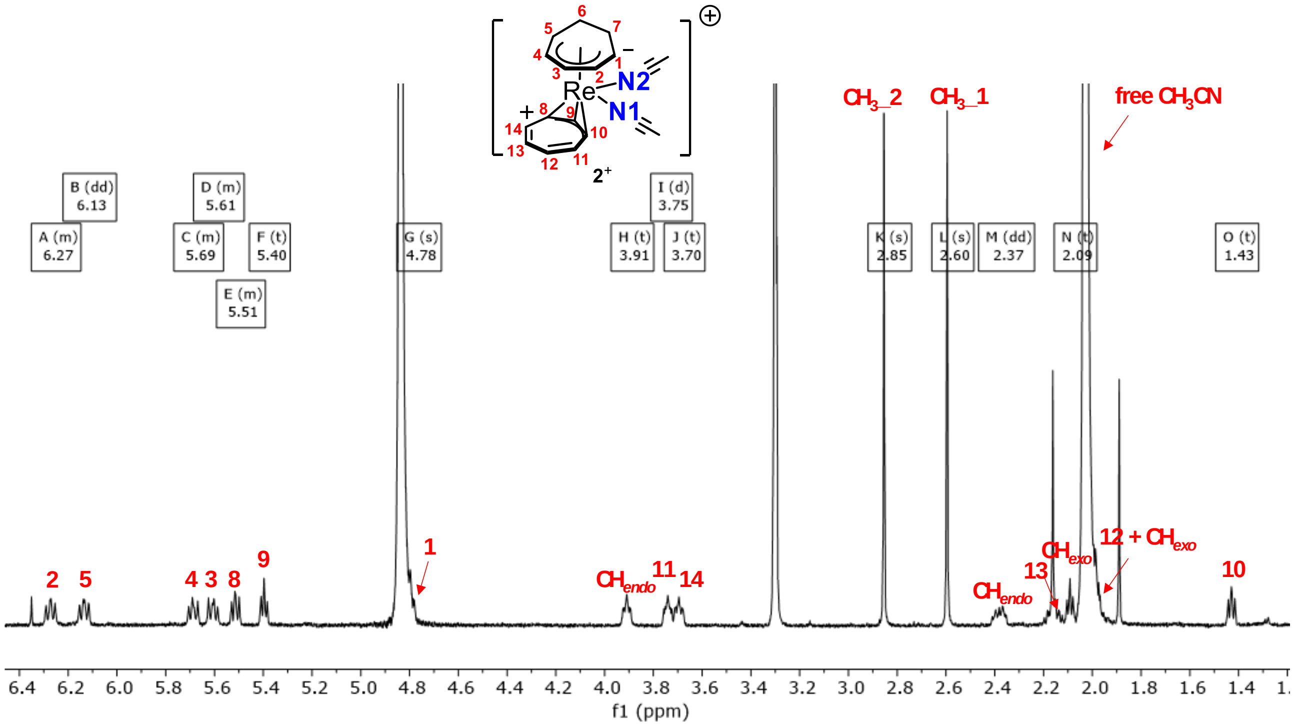

Figure S7. ^{1}H NMR spectrum in methanol-d_4 of $[Re(\eta^3\text{-}C_7H_7)(\eta^5\text{-}C_7H_9)(NCCH_3)_2]^+$ (**2**$^+$).

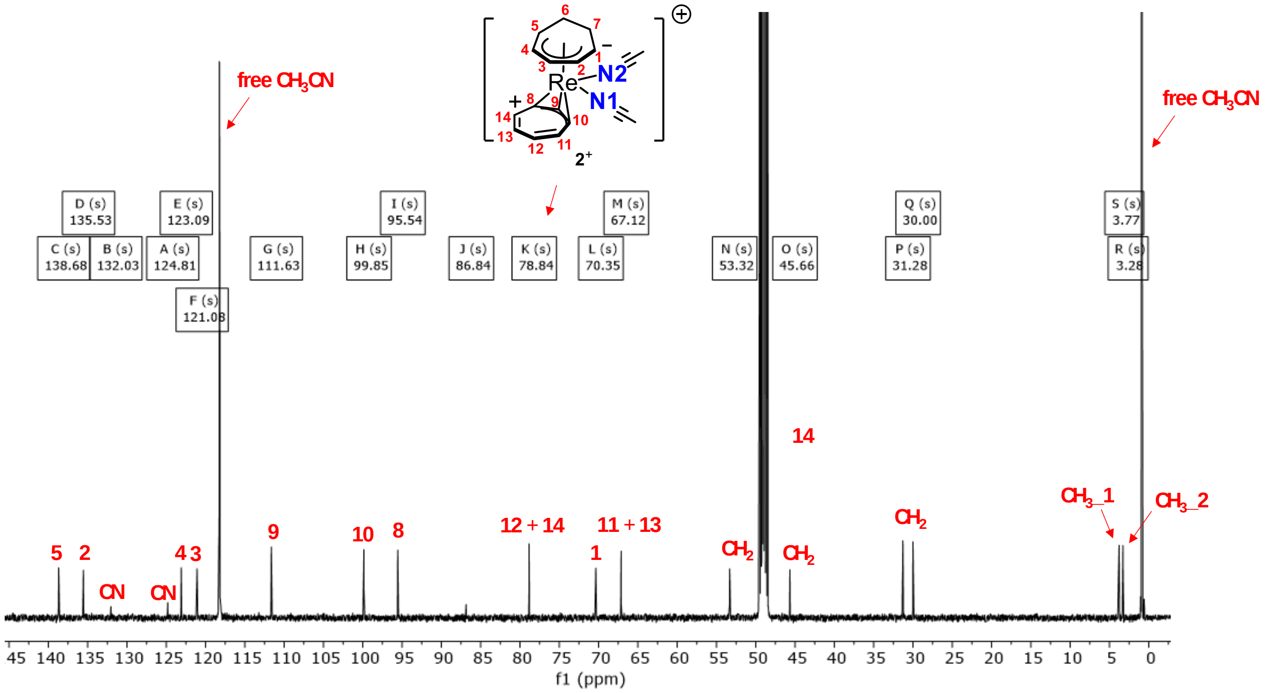

Figure S8. ^{13}C NMR spectrum in methanol-d_4 of $[Re(\eta^3\text{-}C_7H_7)(\eta^5\text{-}C_7H_9)(NCCH_3)_2]^+$ (**2**$^+$).

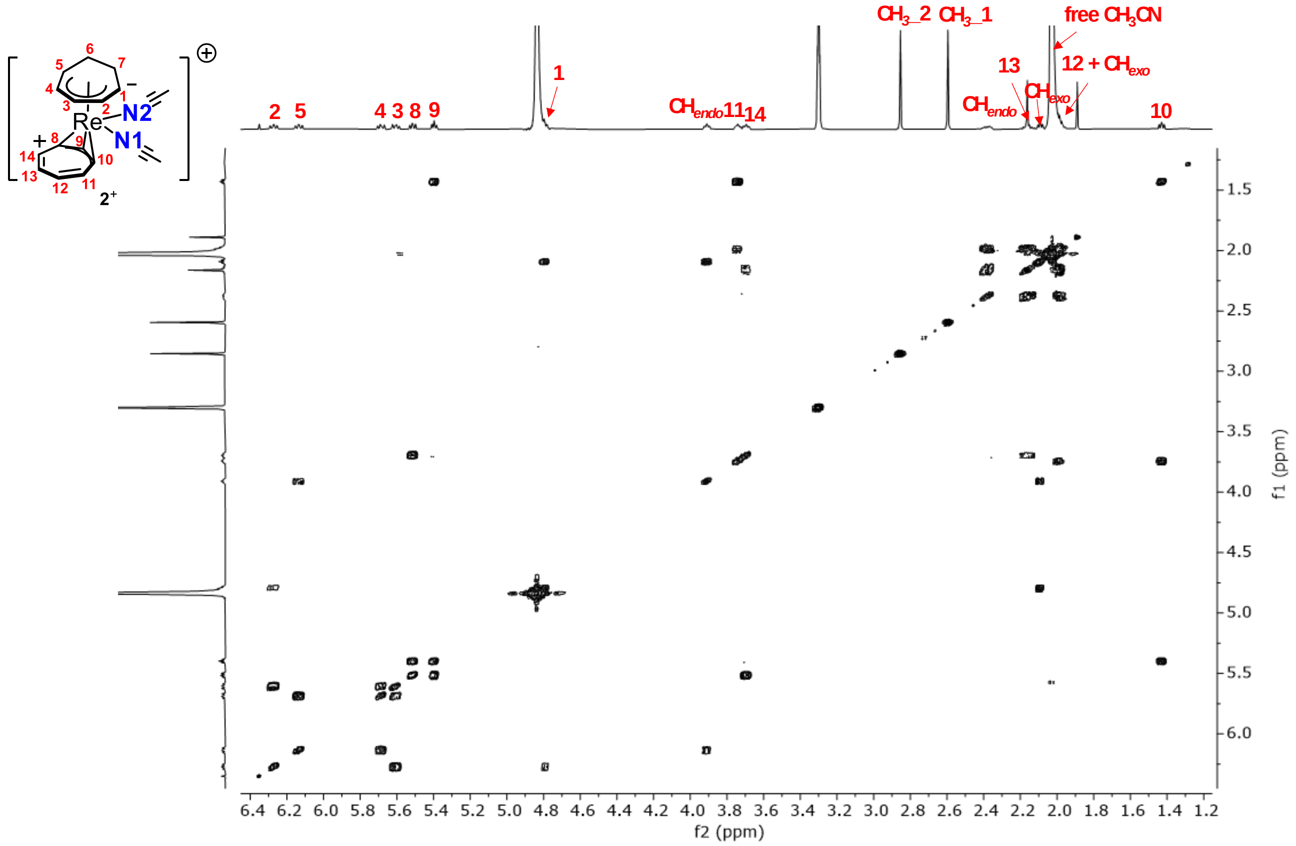

Figure S9. ^{1}H-^{1}H COSY spectrum in methanol-*d*$_4$ of [Re(η^3-C_7H_7)(η^5-C_7H_9)($NCCH_3$)$_2$]$^+$ (**2**$^+$).

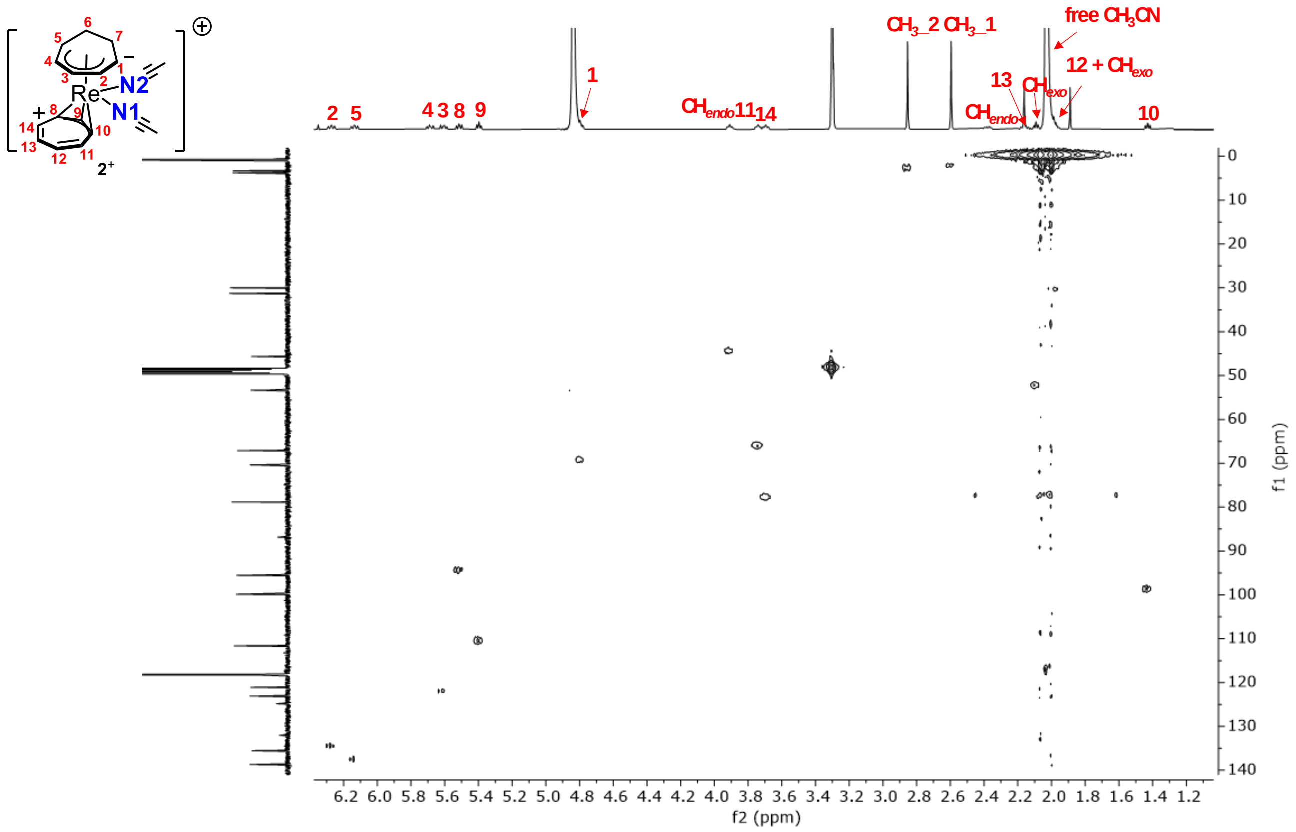

Figure S10. ^{1}H-^{13}C HSQC spectrum in methanol-*d*$_4$ of [Re(η^3-C_7H_7)(η^5-C_7H_9)($NCCH_3$)$_2$]$^+$ (**2**$^+$).

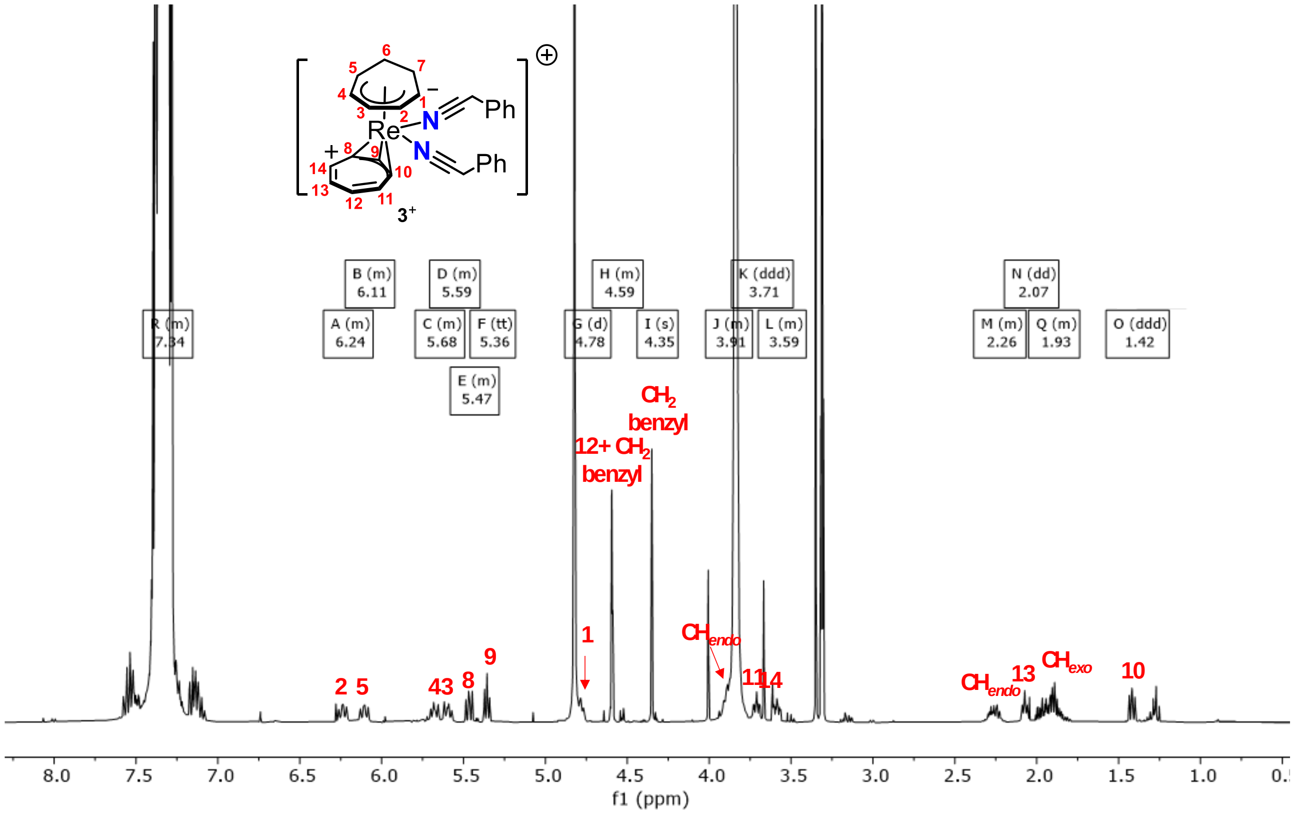

Figure S11. ^{1}H NMR spectrum in methanol-d_4 of [Re(η^3-C_7H_7)(η^5-C_7H_9)($NCCH_2Ph)_2$]$^+$ (**3**$^+$).

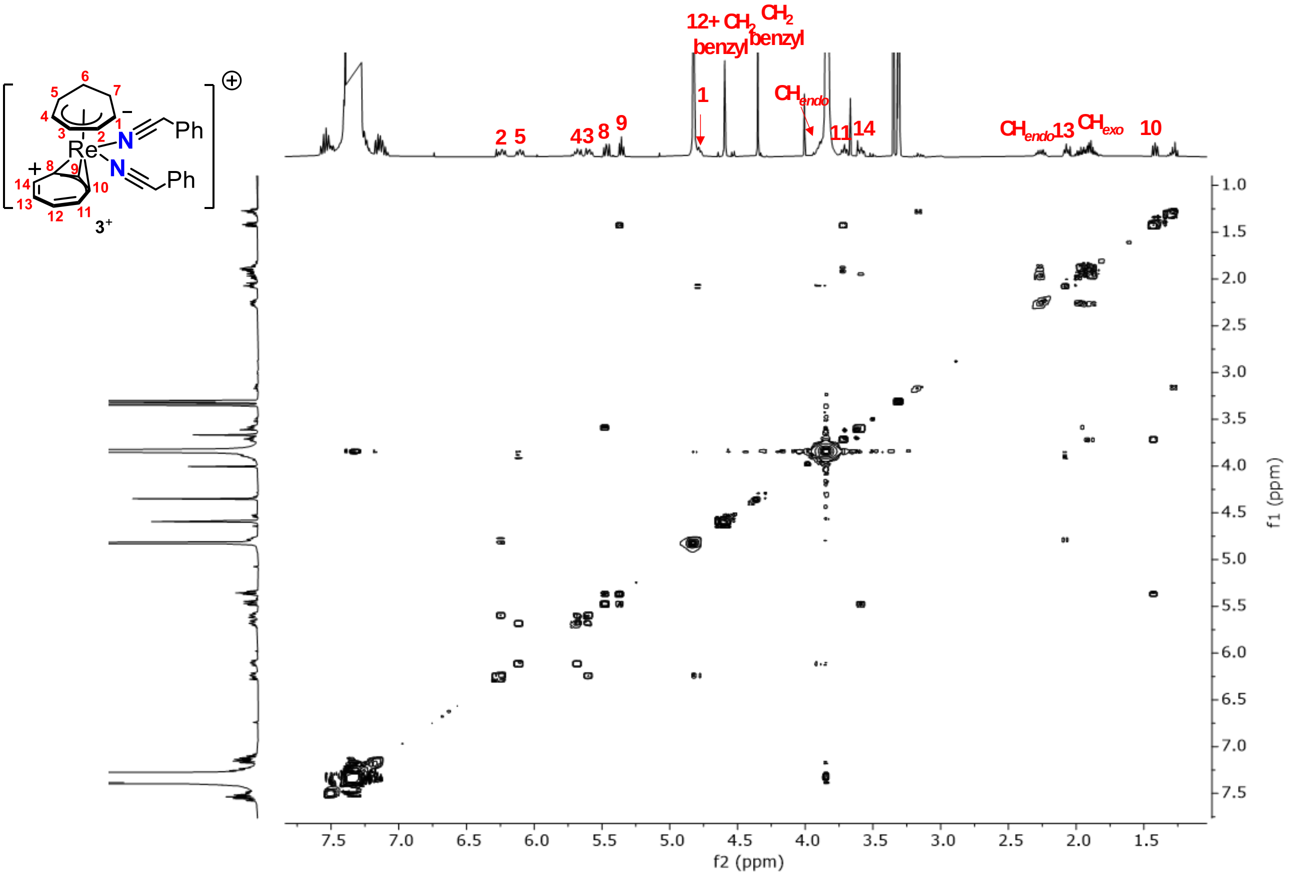

Figure S12. ^{1}H-^{1}H COSY spectrum in methanol-d_4 of [Re(η^3-C_7H_7)(η^5-C_7H_9)($NCCH_2Ph)_2$]$^+$ (**3**$^+$).

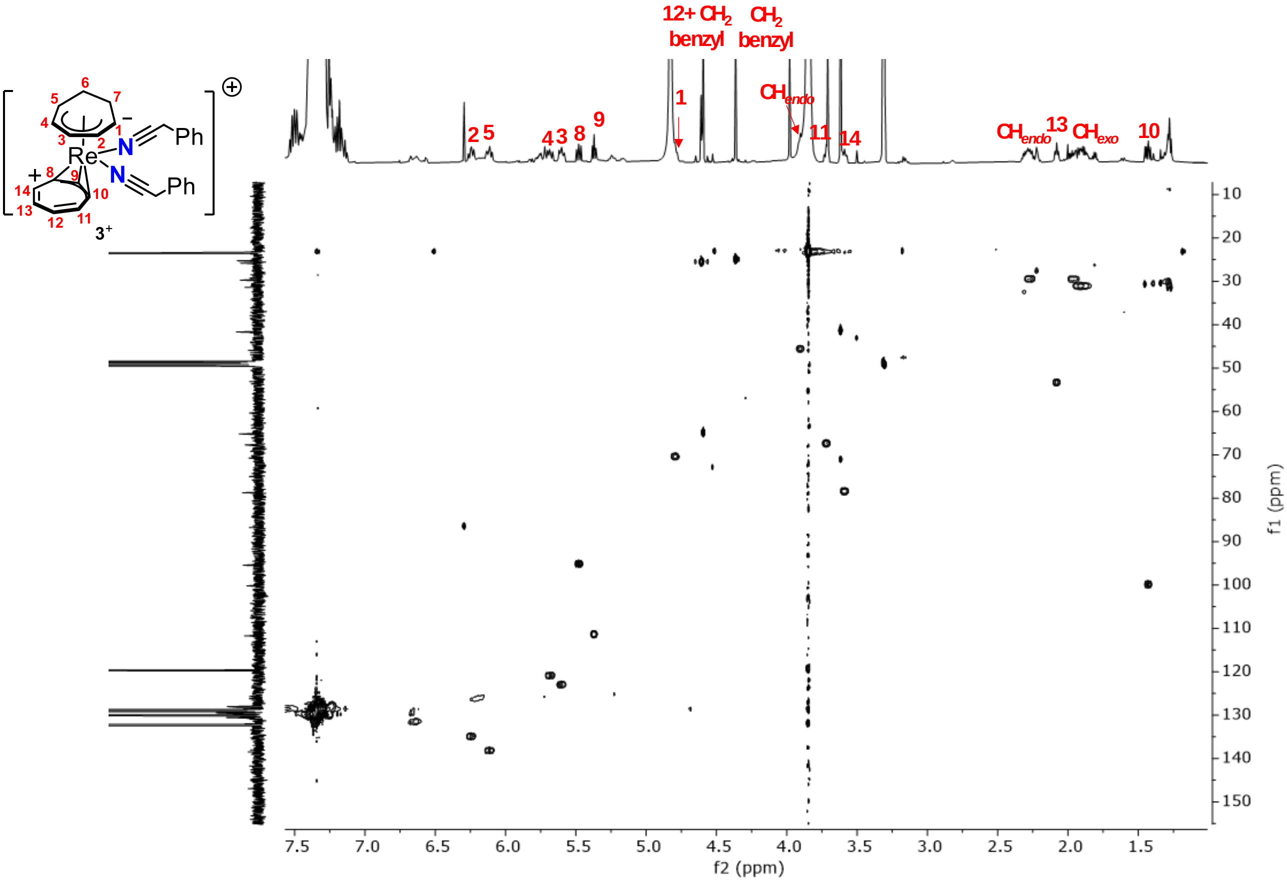

Figure S13. ^{1}H-^{13}C HSQC spectrum in methanol-d_4 of $[Re(\eta^3\text{-}C_7H_7)(\eta^5\text{-}C_7H_9)(NCCH_2Ph)_2]^+$ (**3**$^+$).

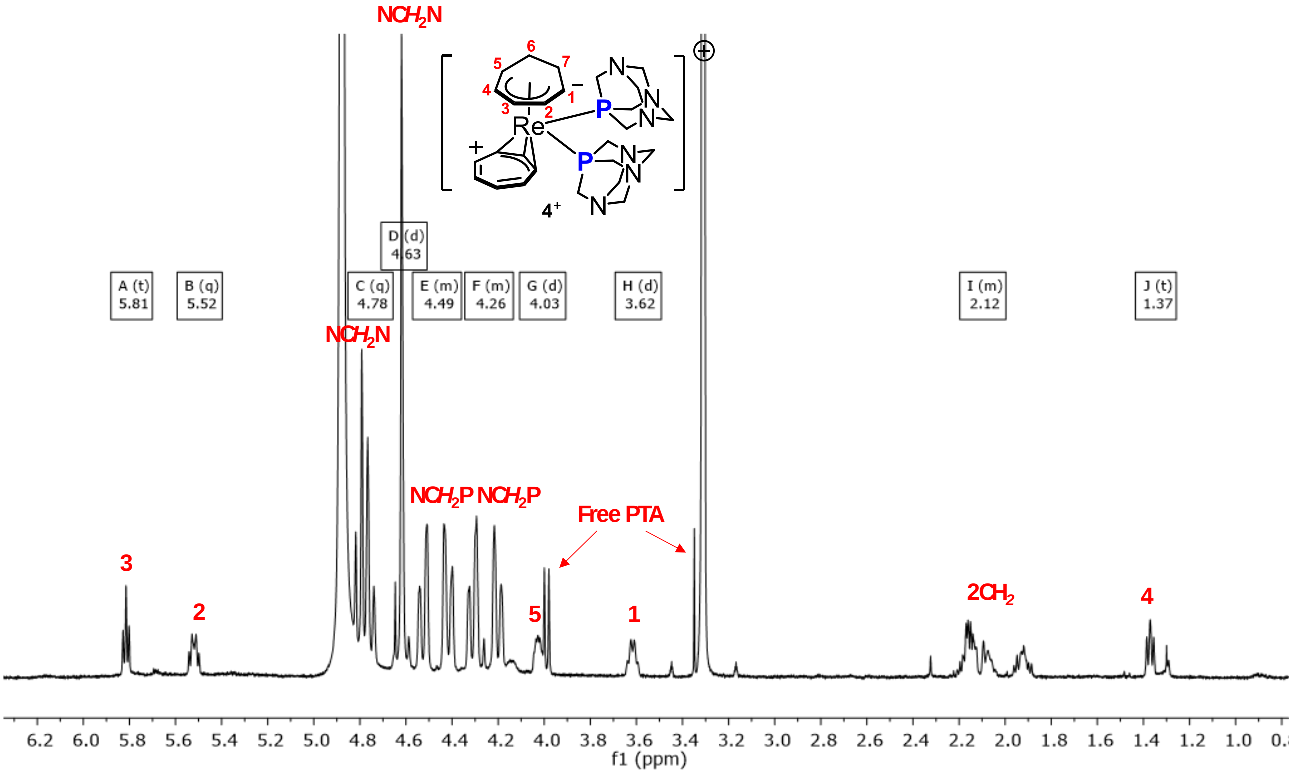

Figure S14. ^{1}H NMR spectrum in methanol-d_4 of $[Re(\eta^3\text{-}C_7H_7)(\eta^7\text{-}C_7H_9)(PTA)_2]^+$ (**4**$^+$).

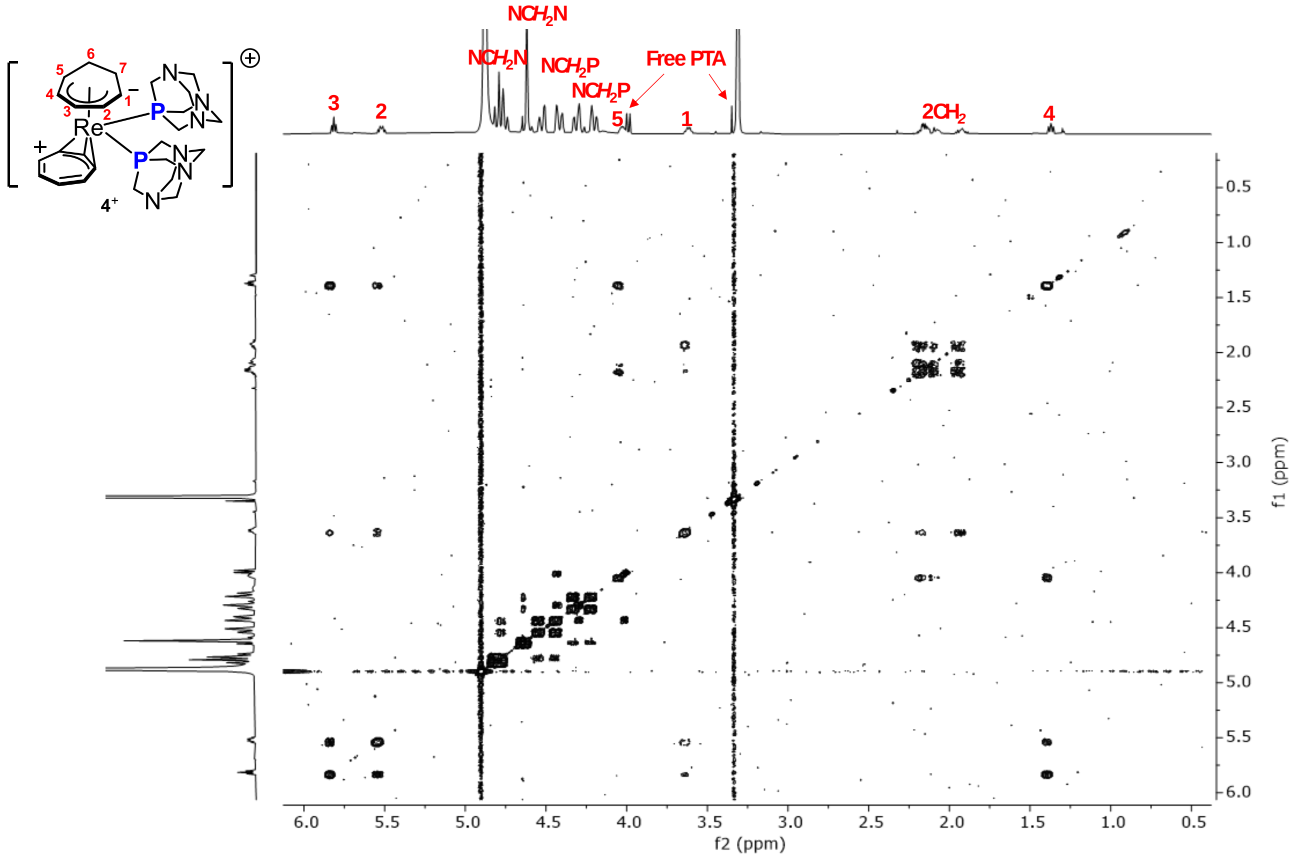

Figure S15. ^{1}H-^{1}H COSY spectrum in methanol-d_4 of $[Re(\eta^3\text{-}C_7H_7)(\eta^5\text{-}C_7H_9)(PTA)_2]^+$ (**4**$^+$).

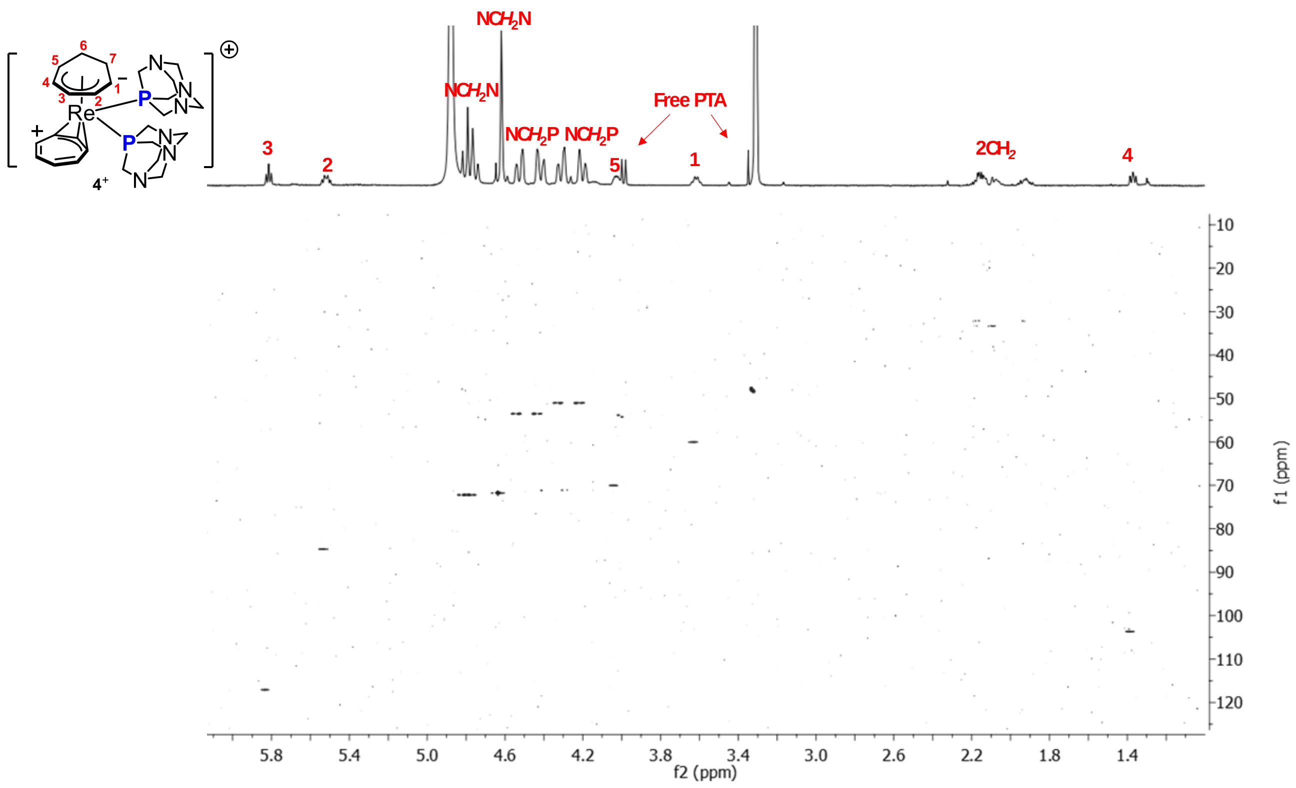

Figure S16. ^{1}H-^{13}C HSQC spectrum in methanol-d_4 of $[Re(\eta^3\text{-}C_7H_7)(\eta^5\text{-}C_7H_9)(PTA)_2]^+$ (**4**$^+$).

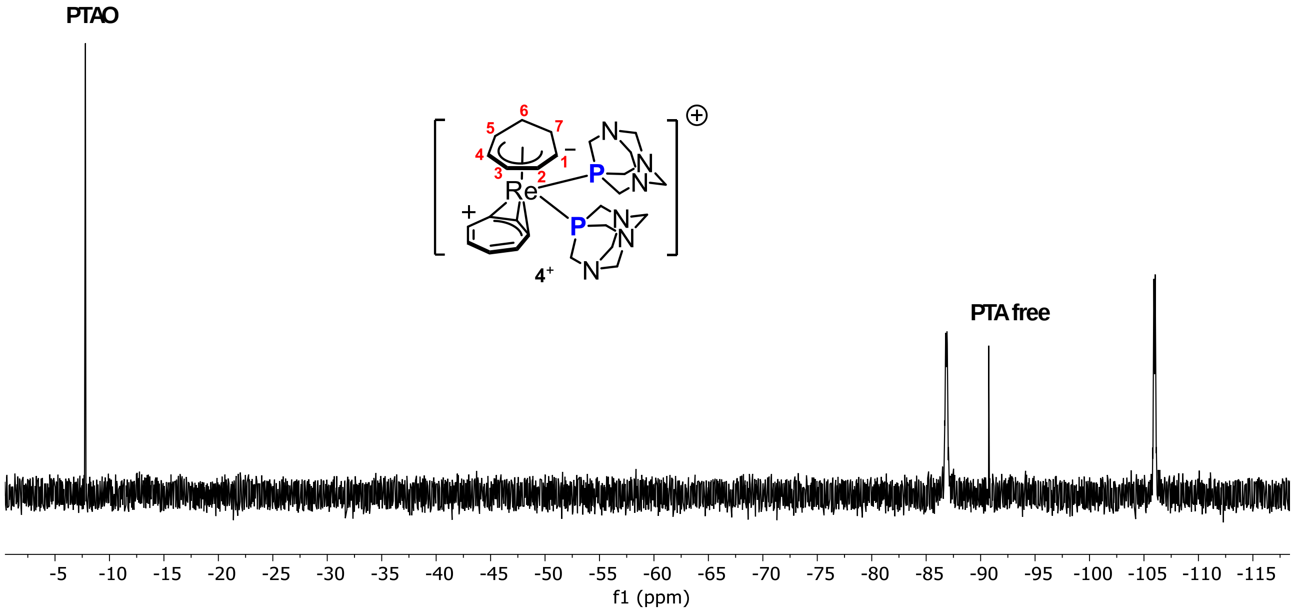

Figure S17. $^{31}P\{^1H\}$ spectrum in methanol-d_4 of $[Re(\eta^3-C_7H_7)(\eta^5-C_7H_9)(PTA)_2]^+$ (**4**$^+$).

Note: Due to the low solubility of $[Re(\eta^3-C_7H_7)(\eta^5-C_7H_9)(dppe)_2]^+$ (**5**$^+$) in most common solvents (methanol, acetone, DMSO, acetonitrile, water, chloroform), it could be only partially characterized by NMR. Assignments are done in comparison with **4**$^+$.

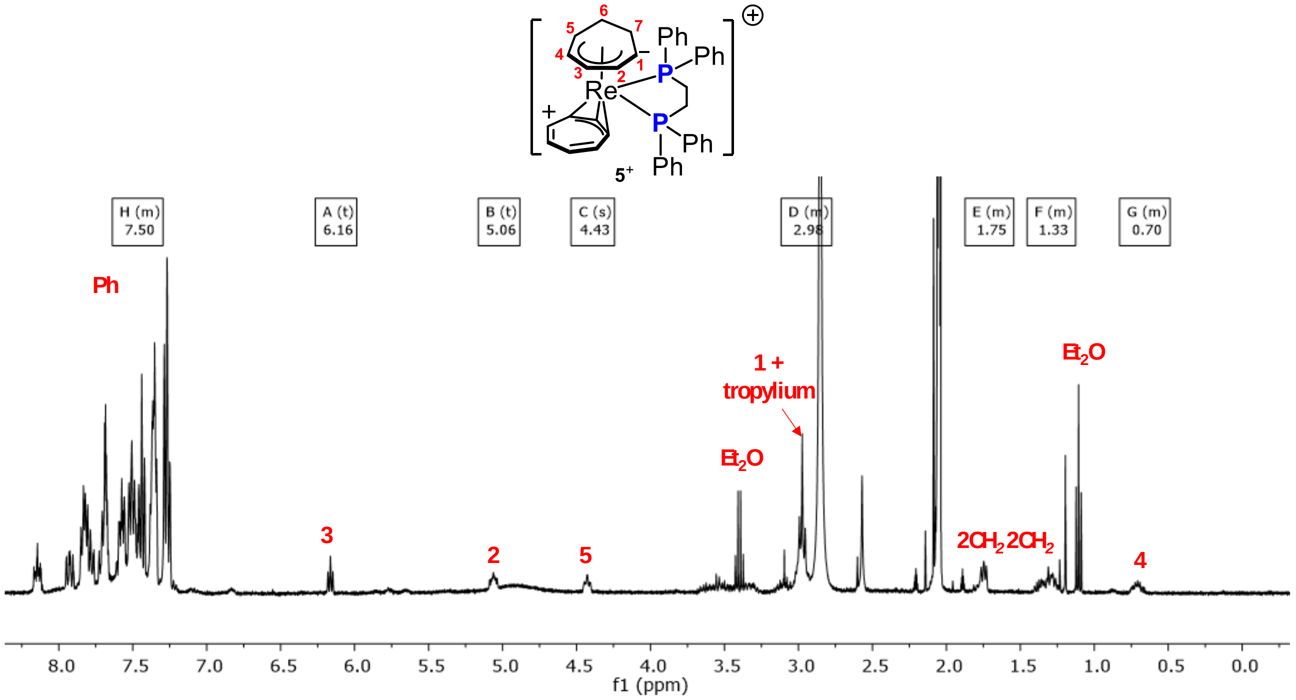

Figure S18. ^1H spectrum in acetone-d_6 of $[Re(\eta^3-C_7H_7)(\eta^5-C_7H_9)(dppe)_2]^+$ (**5**$^+$).

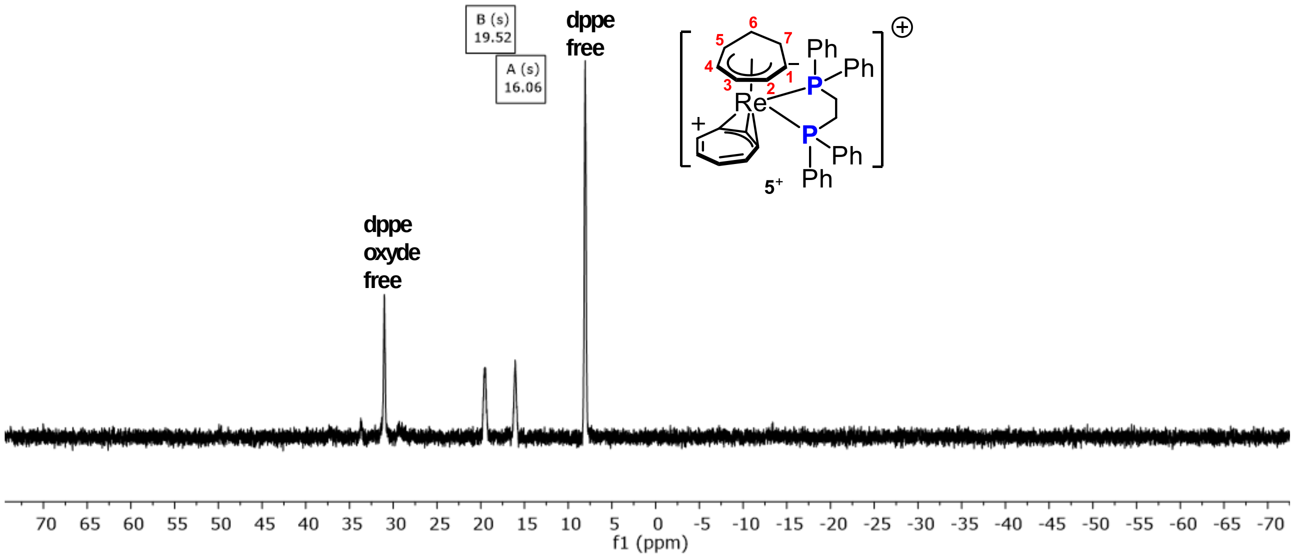

Figure S19. $^{31}P\{^1H\}$ spectrum in acetone-d_6 of $[Re(\eta^3\text{-}C_7H_7)(\eta^5\text{-}C_7H_9)(dppe)_2]^+$ (**5**$^+$).

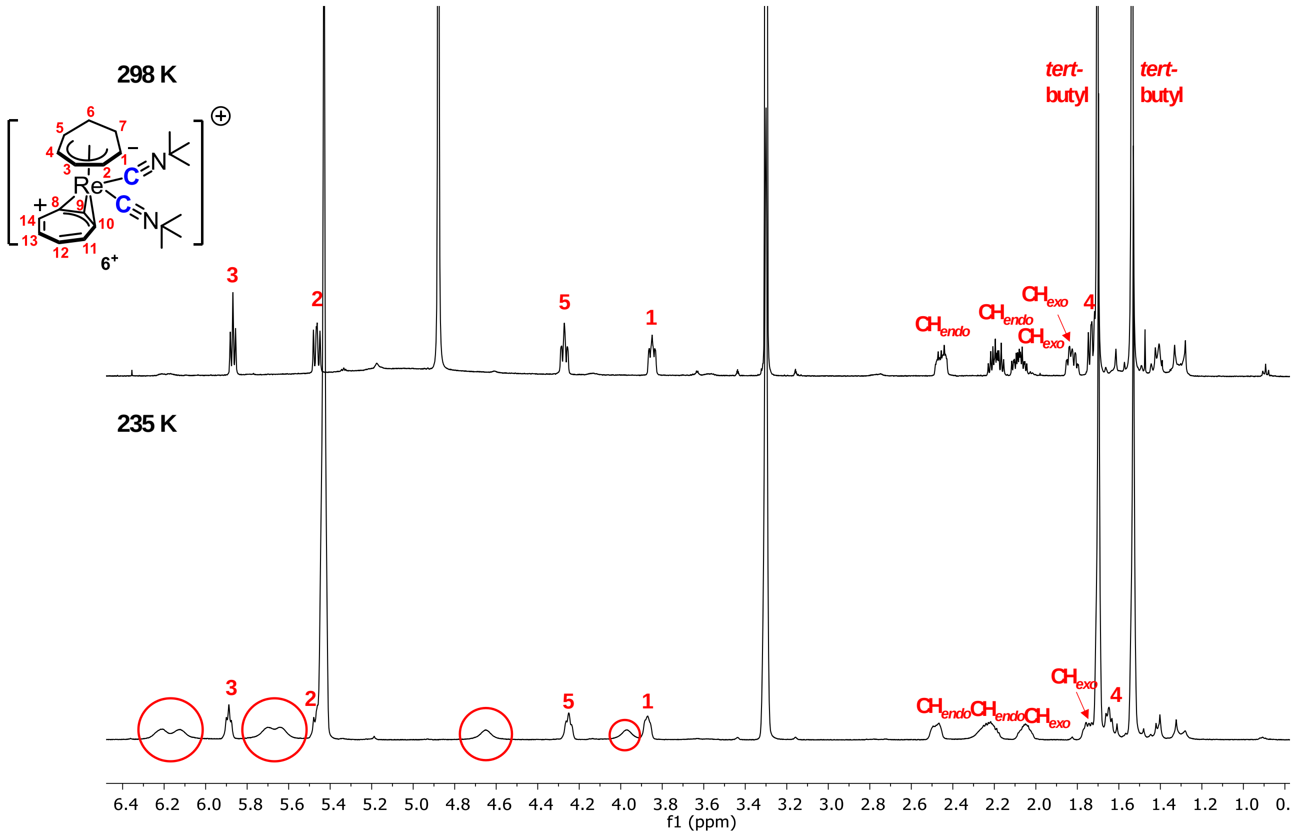

Figure S20. ^1H spectrum in methanol-d_4 of $[Re(\eta^3\text{-}C_7H_7)(\eta^5\text{-}C_7H_9)(CN\text{-}^tBu)_2]^+$ (**6**$^+$) at 298 K (top) and 235 K (bottom)

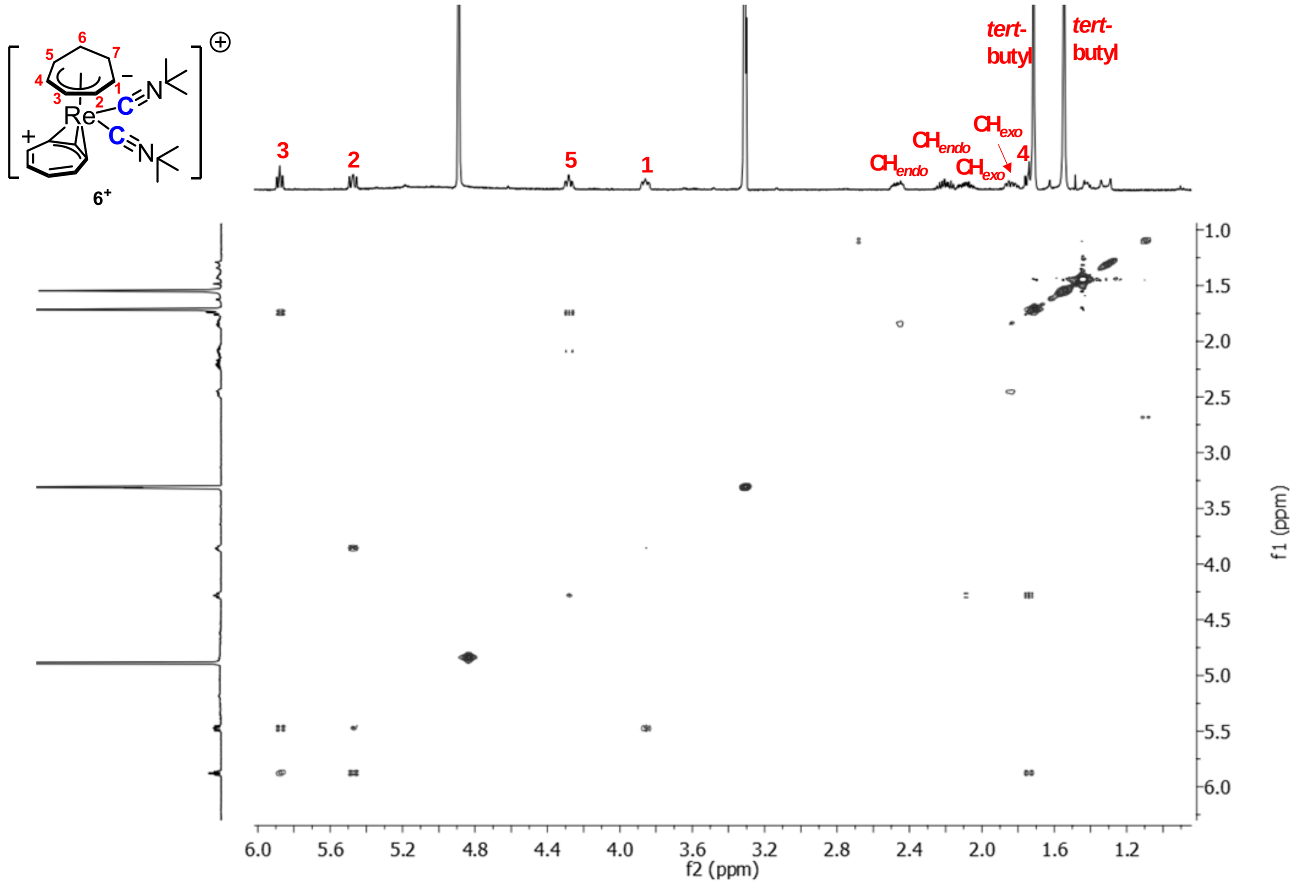

Figure S21. ^{1}H-^{1}H COSY spectrum in methanol-d_4 of $[Re(\eta^3\text{-}C_7H_7)(\eta^5\text{-}C_7H_9)(CN\text{-}^tBu)_2]^+$ (**6**$^+$).

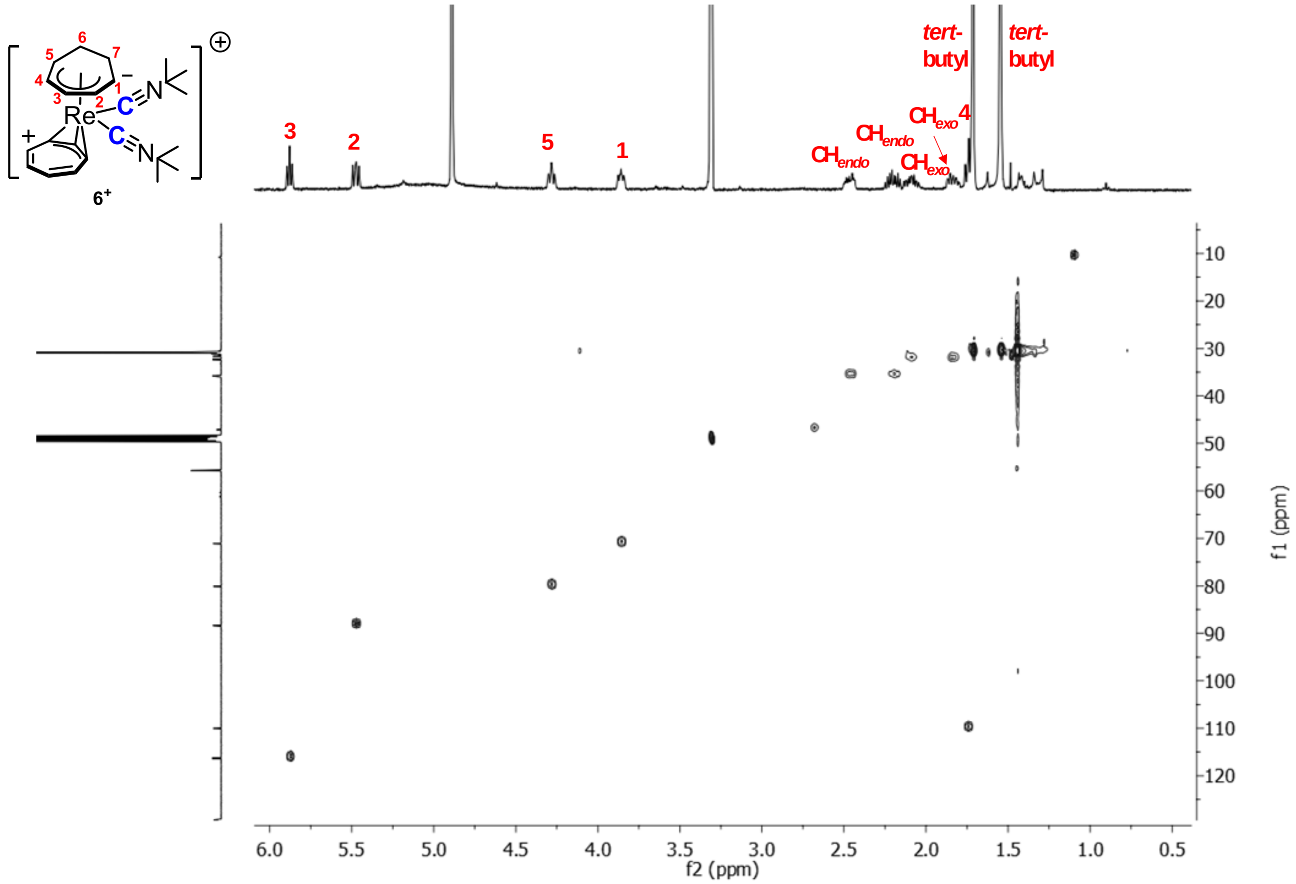

Figure S22. ^{1}H-^{13}C HSQC spectrum in methanol-d_4 of $[Re(\eta^3\text{-}C_7H_7)(\eta^5\text{-}C_7H_9)CN\text{-}^tBu)_2]^+$ (**6**$^+$).

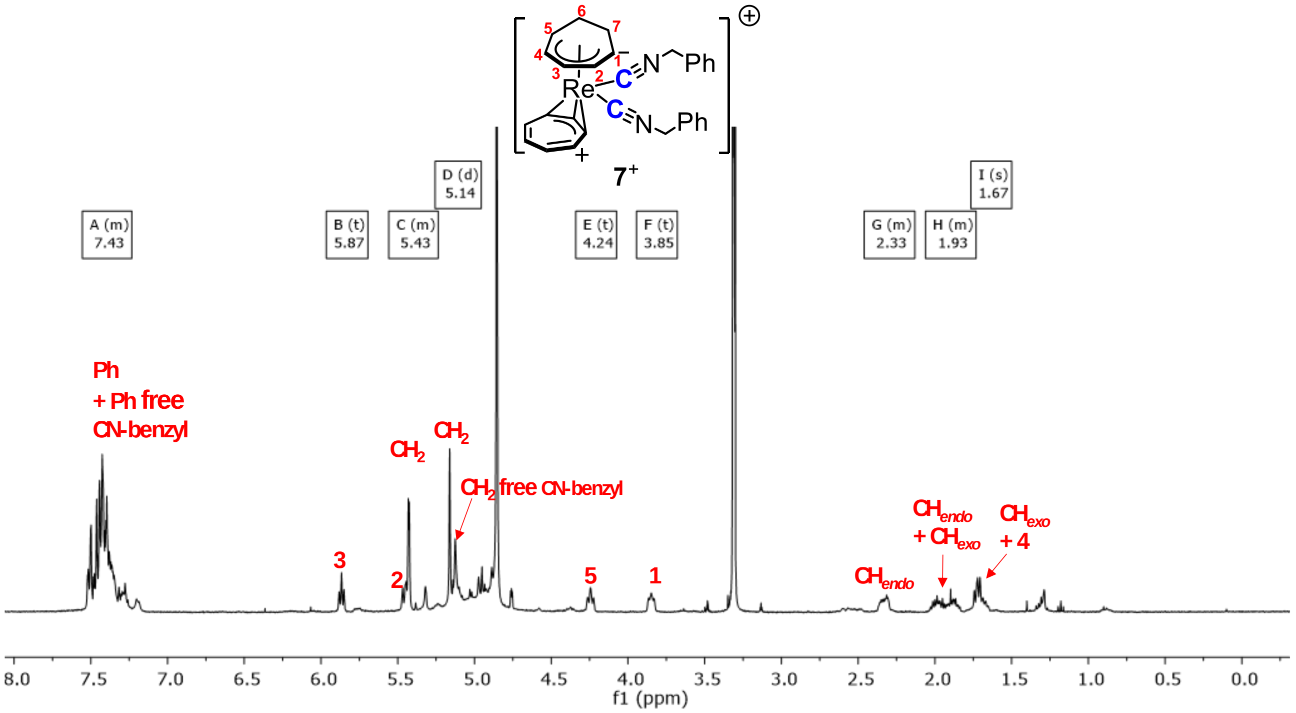

Figure S23. ^{1}H spectrum in methanol-d_4 of $[Re(\eta^3\text{-}C_7H_7)(\eta^5\text{-}C_7H_9)(CN\text{-benzyl})_2]^+$ (**7**$^+$).

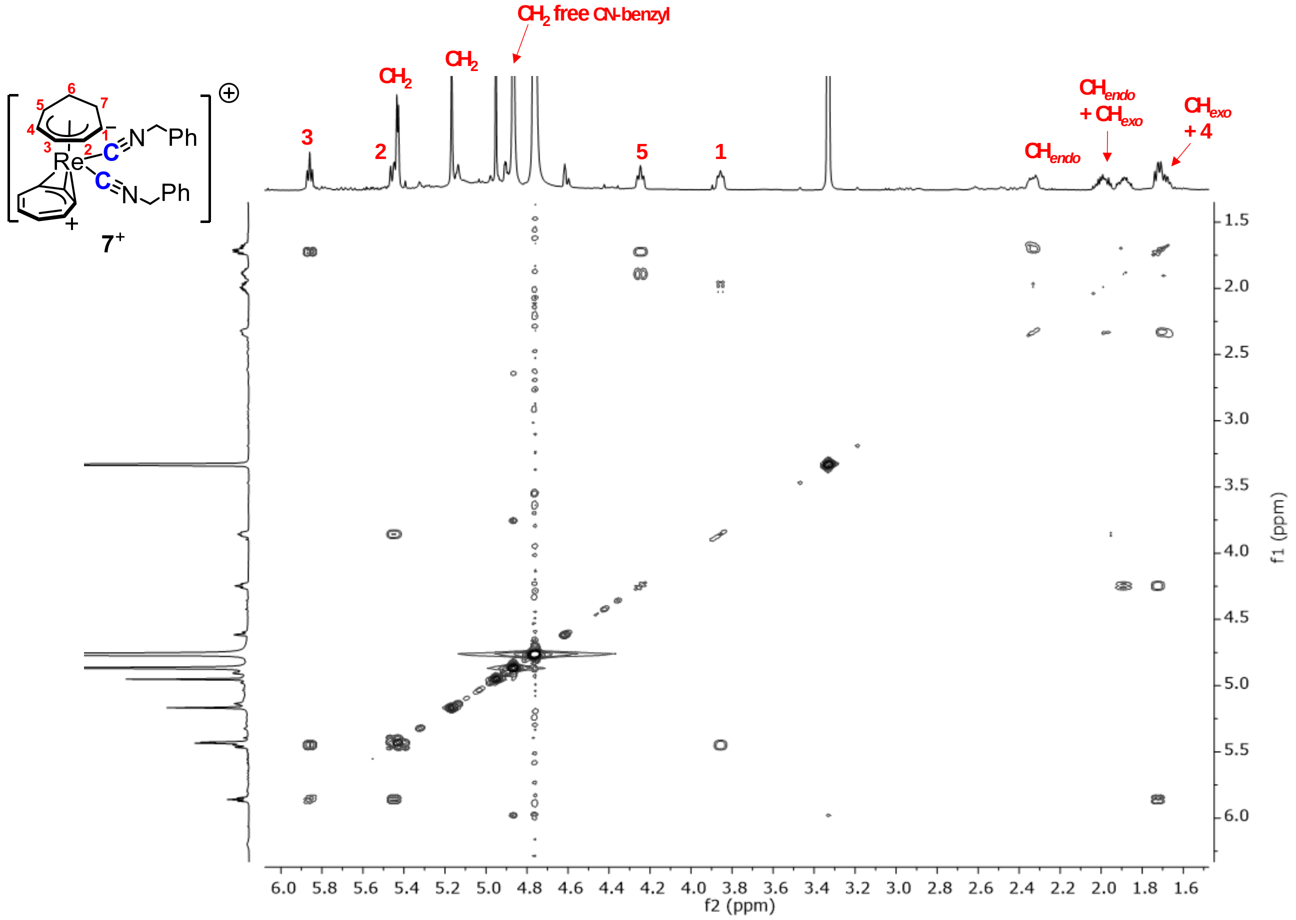

Figure S24. ^{1}H-^{1}H COSY spectrum in methanol-d_4 of $[Re(\eta^3\text{-}C_7H_7)(\eta^5\text{-}C_7H_9)(CN\text{-benzyl})_2]^+$ (**7**$^+$).

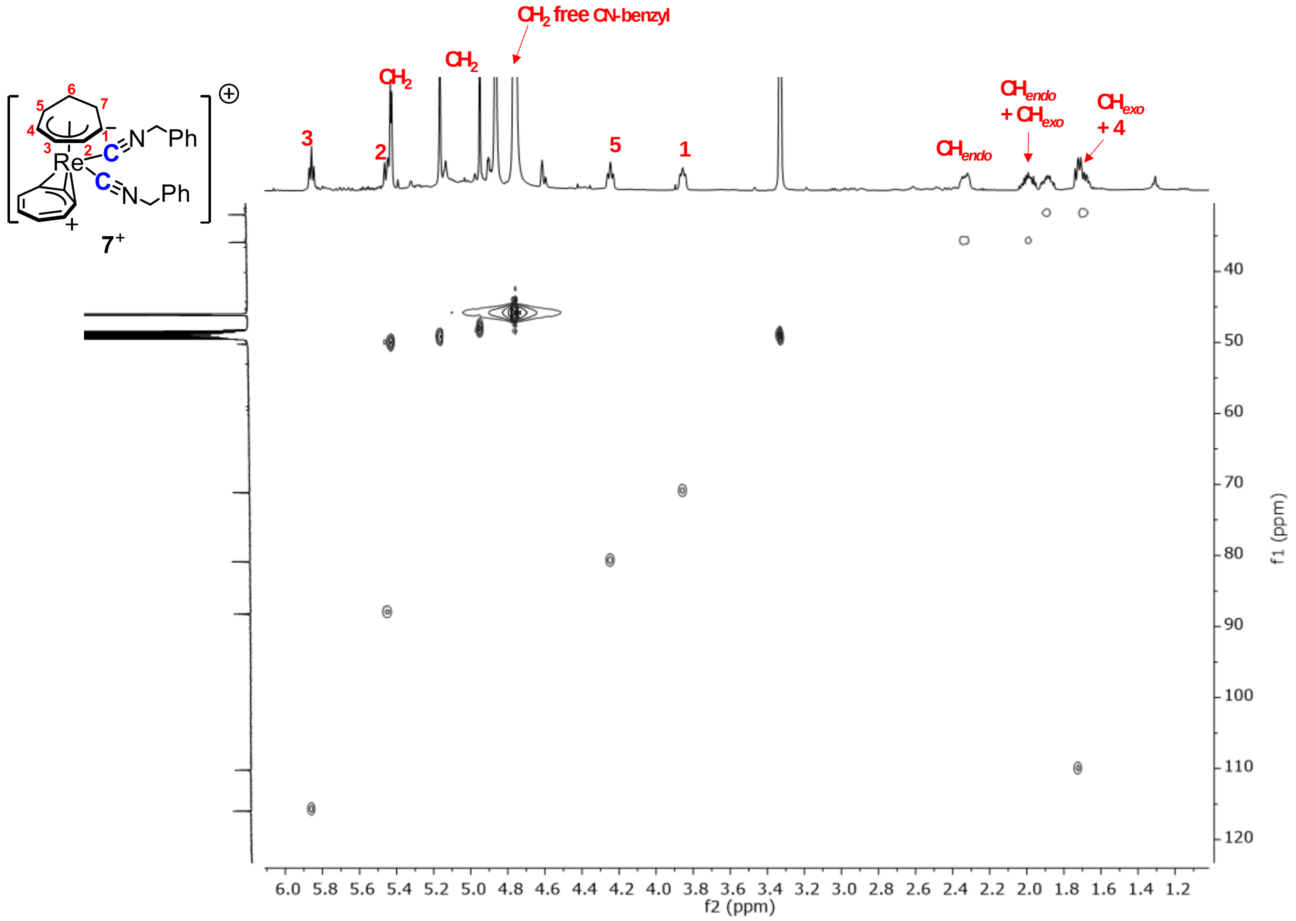

Figure S25. ^{1}H-^{13}C HSQC spectrum in methanol-d_4 of $[Re(\eta^3\text{-}C_7H_7)(\eta^5\text{-}C_7H_9)(CN\text{-benzyl})_2]^+$ ($\mathbf{7}^+$).

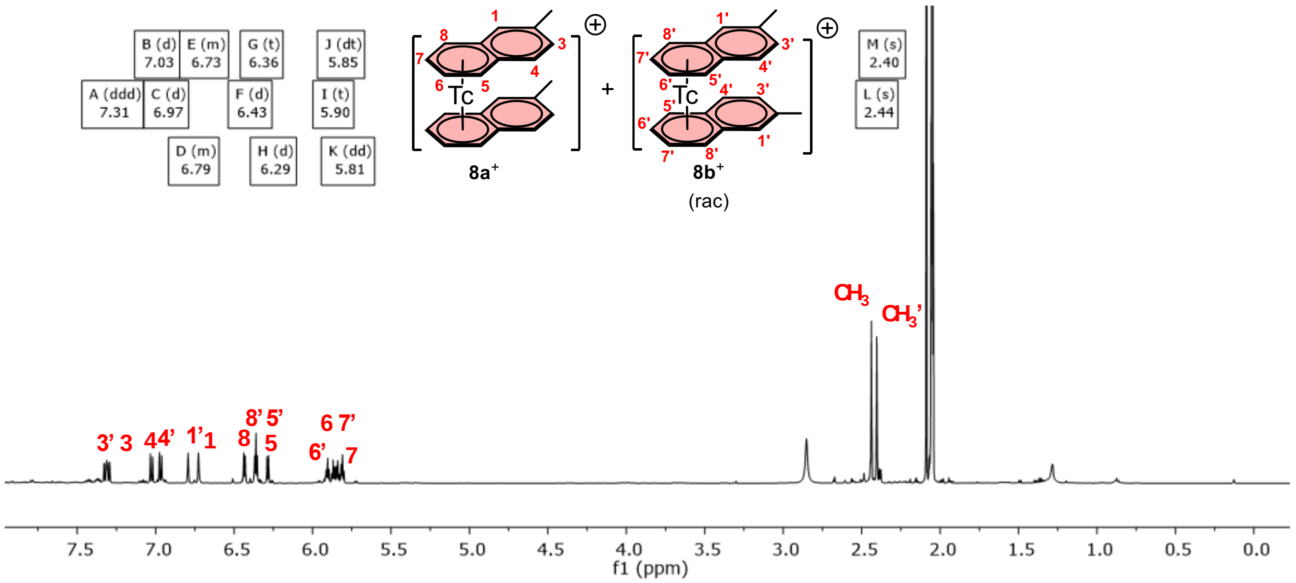

Figure S26. ^{1}H spectrum in acetone-d_6 of $[^{99}Tc(\eta^6\text{-}C_{11}H_{10})_2]^+$ ($\mathbf{8a}^+$ and $\mathbf{8b}^+$).

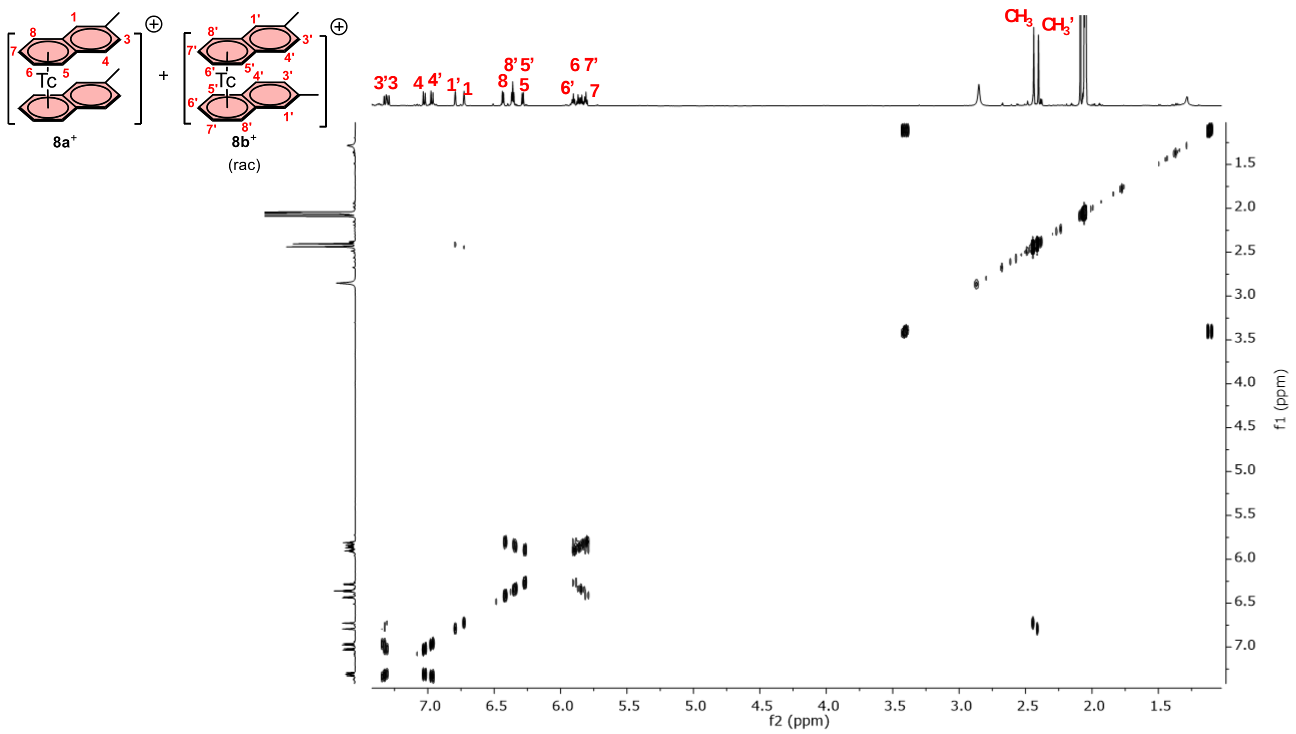

Figure S27. ^{1}H-^{1}H COSY spectrum in acetone-d_6 of $[^{99}Tc(\eta^6\text{-}C_{11}H_{10})_2]^+$ (**8a**$^+$ and **8b**$^+$).

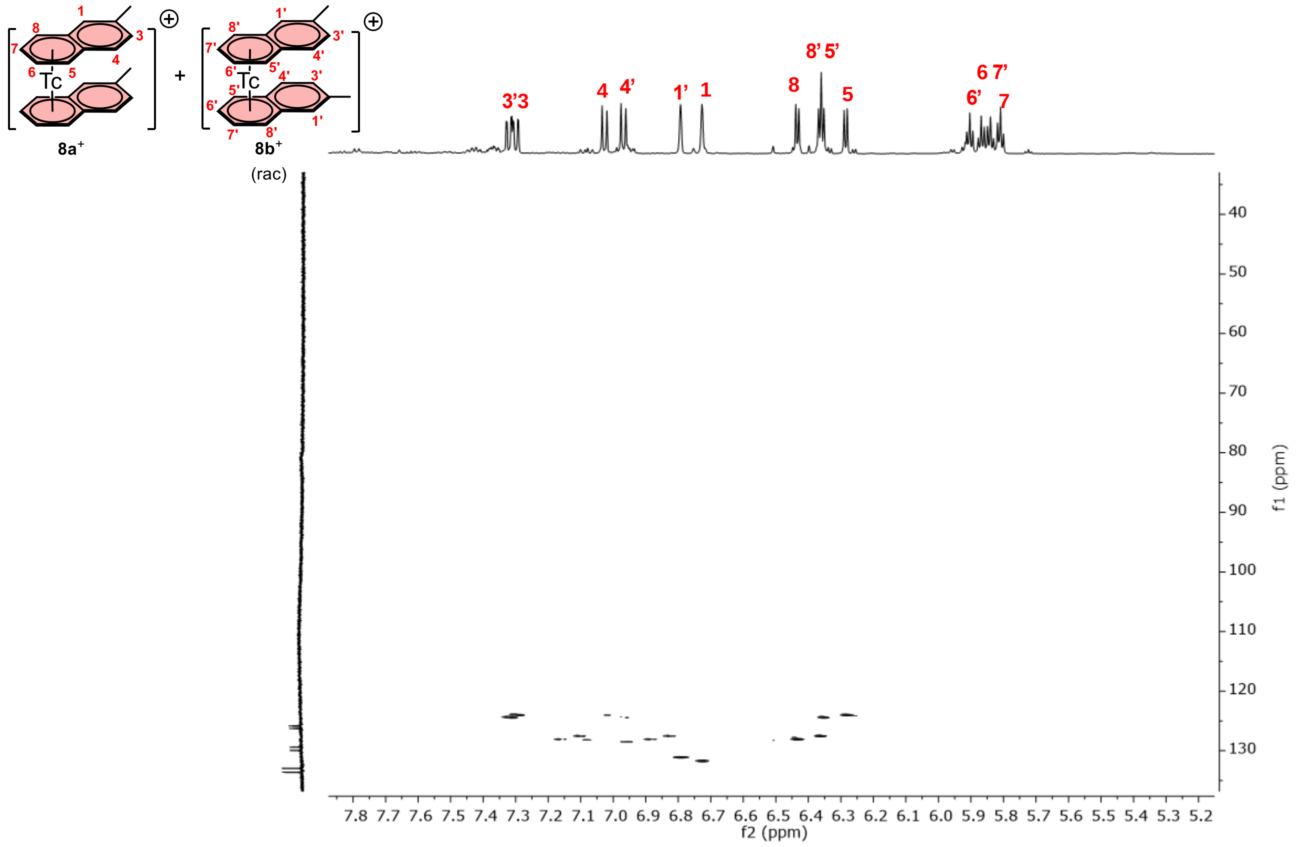

Figure S28. ^{1}H-^{13}C HSQC spectrum in acetone-d_6 of $[^{99}Tc(\eta^6\text{-}C_{11}H_{10})_2]^+$ (**8a**$^+$ and **8b**$^+$), aromatic region.

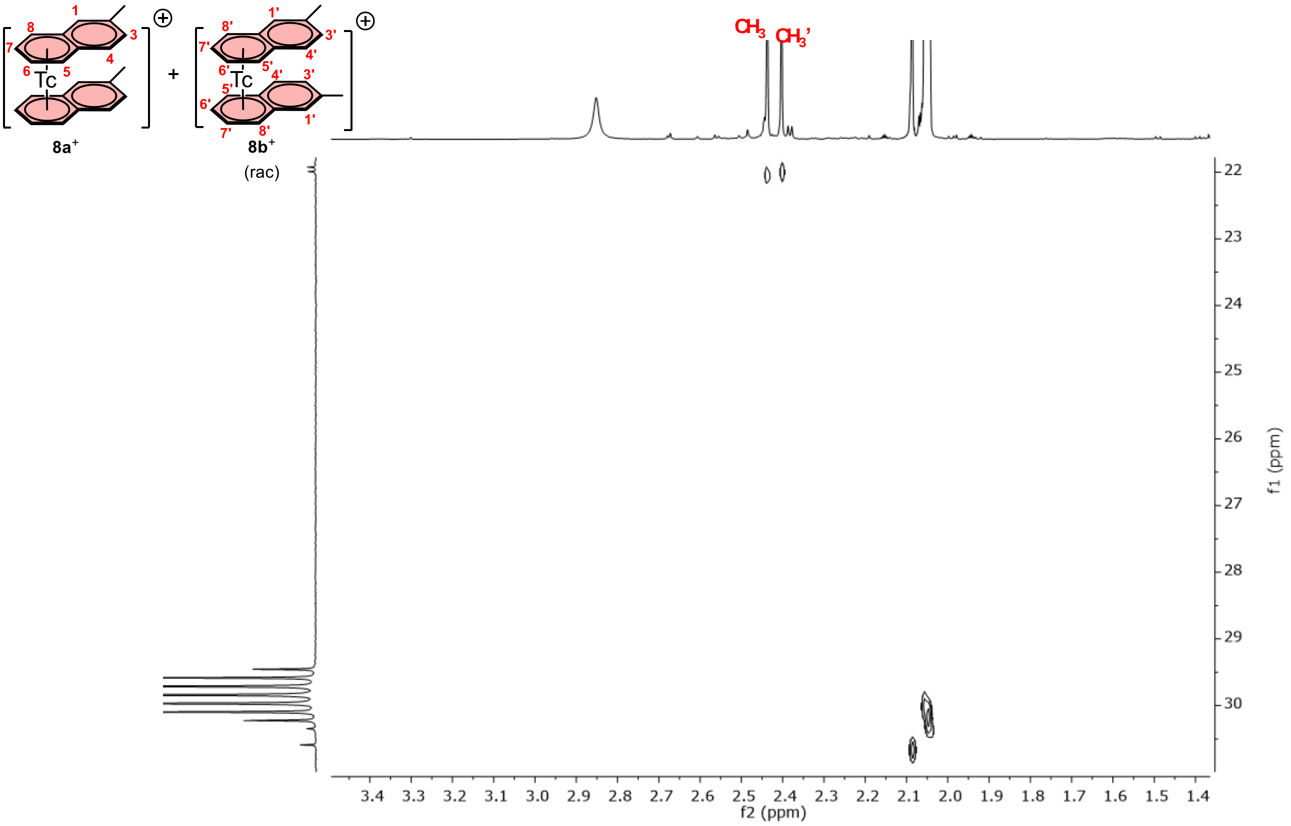

Figure S29. ^{1}H-^{13}C HSQC spectrum in acetone-d_6 of $[^{99}Tc(\eta^6\text{-}C_{11}H_{10})_2]^+$ (**8a**$^+$ and **8b**$^+$), aliphatic region.

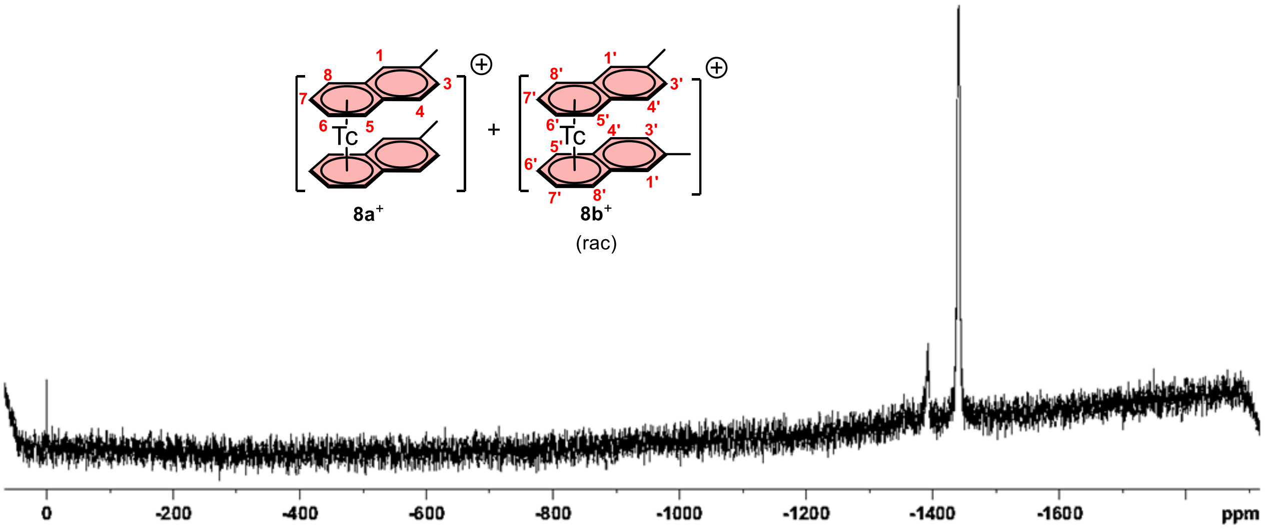

Figure S30. ^{99}Tc spectrum in acetone-d_6 of $[^{99}Tc(\eta^6\text{-}C_{11}H_{10})_2]^+$ (**8a**$^+$ and **8b**$^+$).

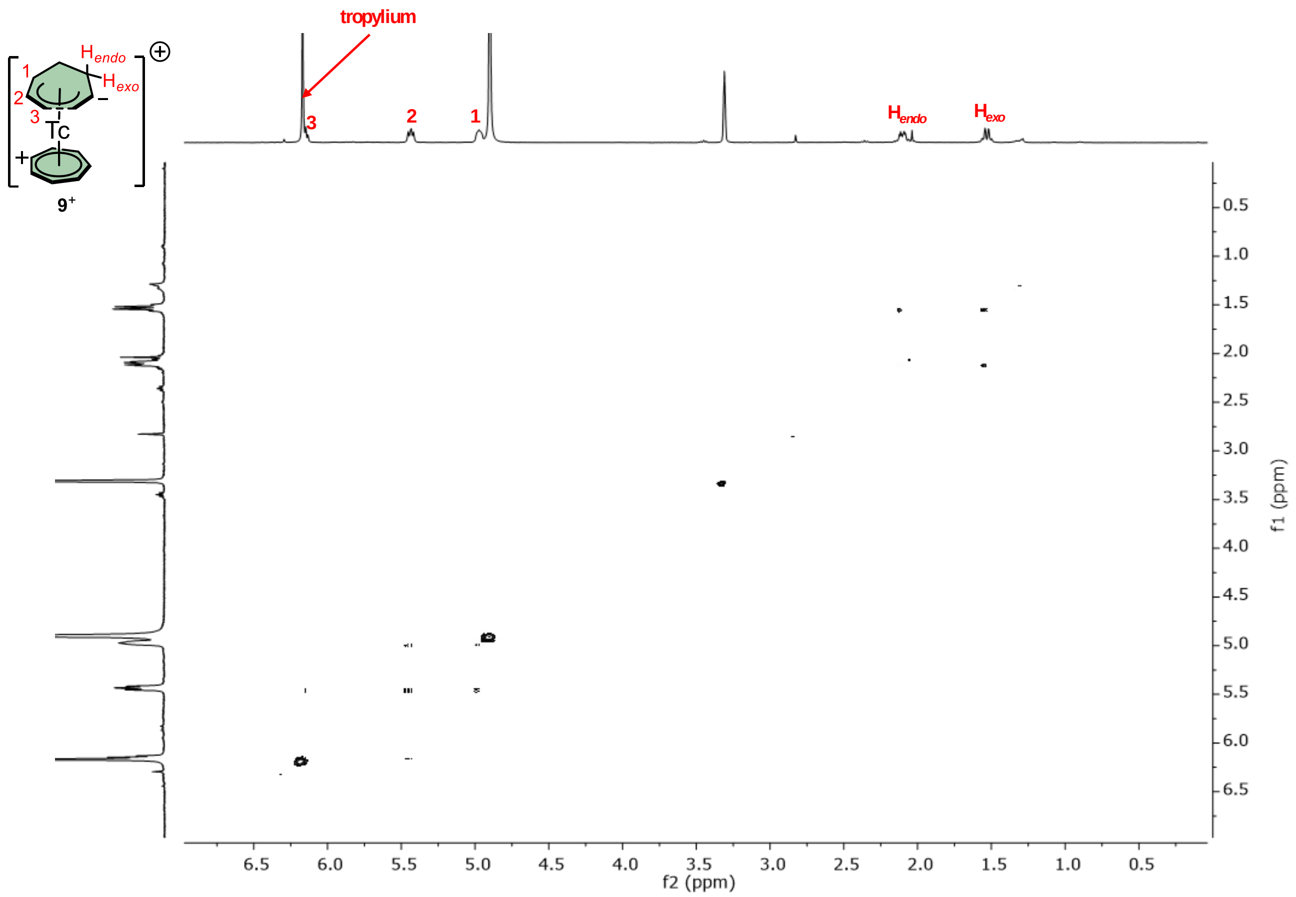

Figure S31. ^{1}H-^{1}H COSY spectrum in methanol-d_4 of $[^{99}Tc(\eta^7\text{-}C_7H_7)(\eta^5\text{-}C_7H_9)]^+$ (**9**$^+$).

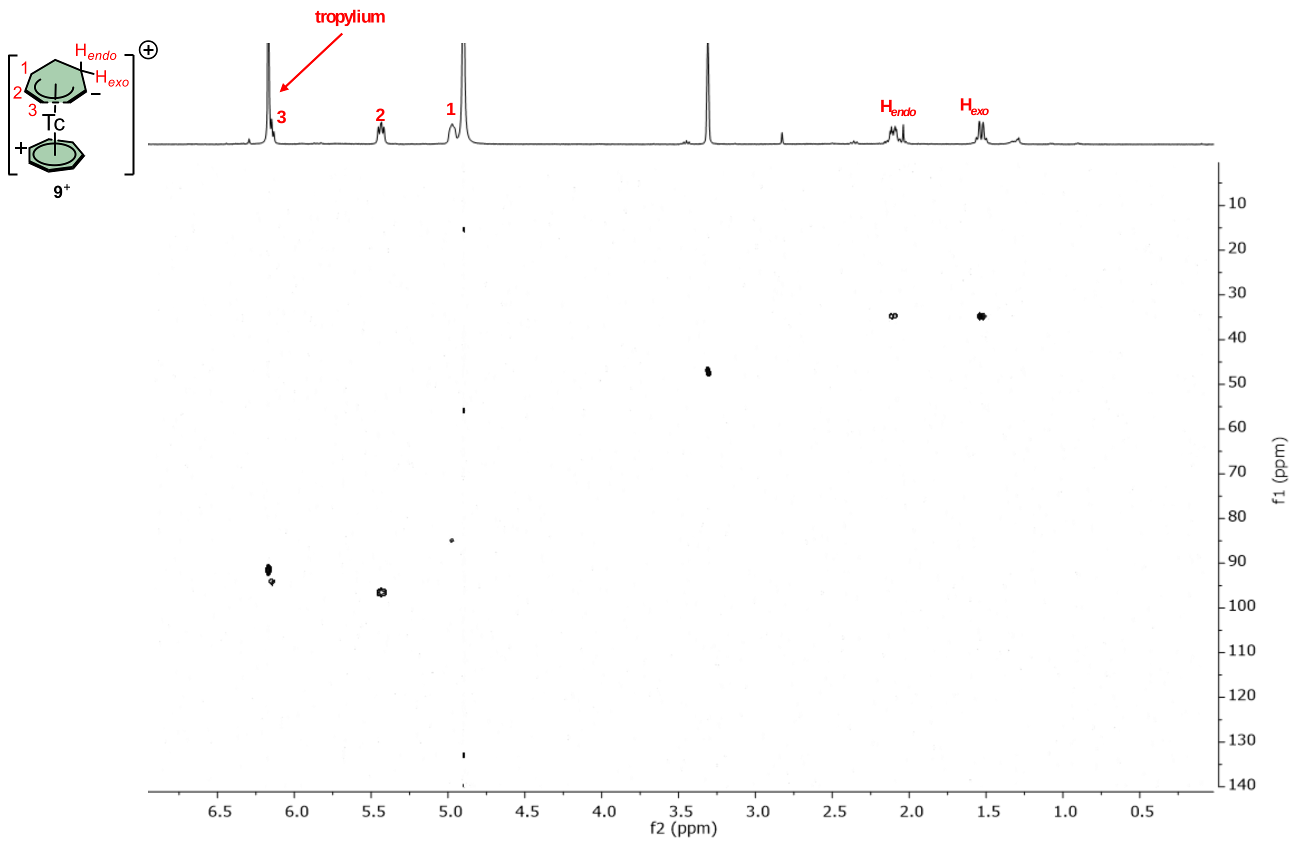

Figure S32. ^{1}H-^{13}C HSQC spectrum in methanol-d_4 of $[^{99}Tc(\eta^7\text{-}C_7H_7)(\eta^5\text{-}C_7H_9)]^+$ (**9**$^+$).

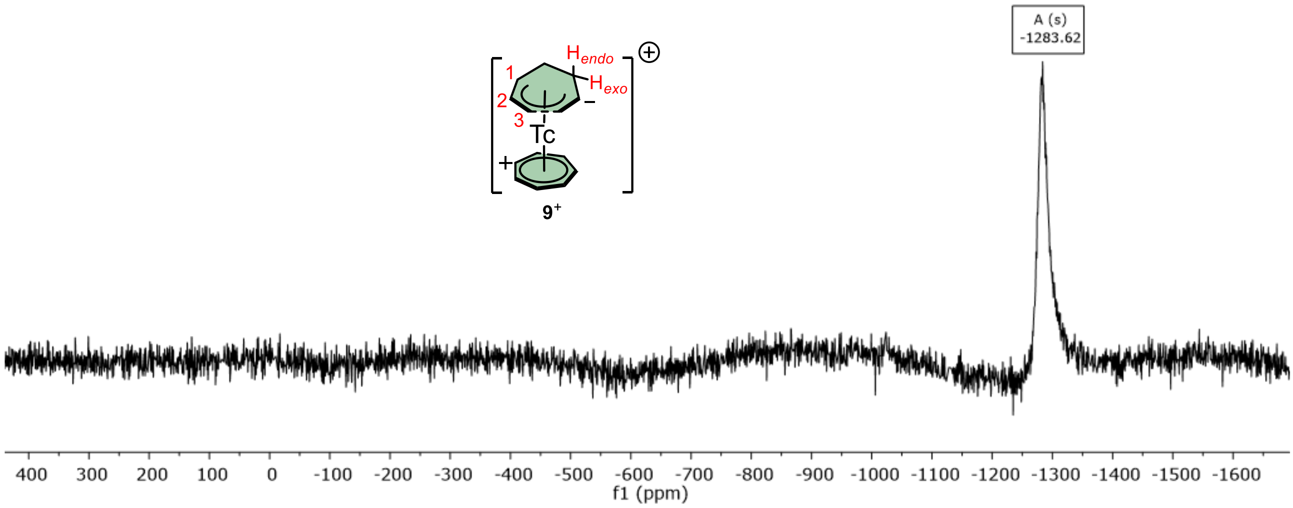

Figure S33. ^{99}Tc spectrum in methanol-d_4 of [^{99}Tc(η^7-C_7H_7)(η^5-C_7H_9)]$^+$ (**9**$^+$).

Kinetics measurements

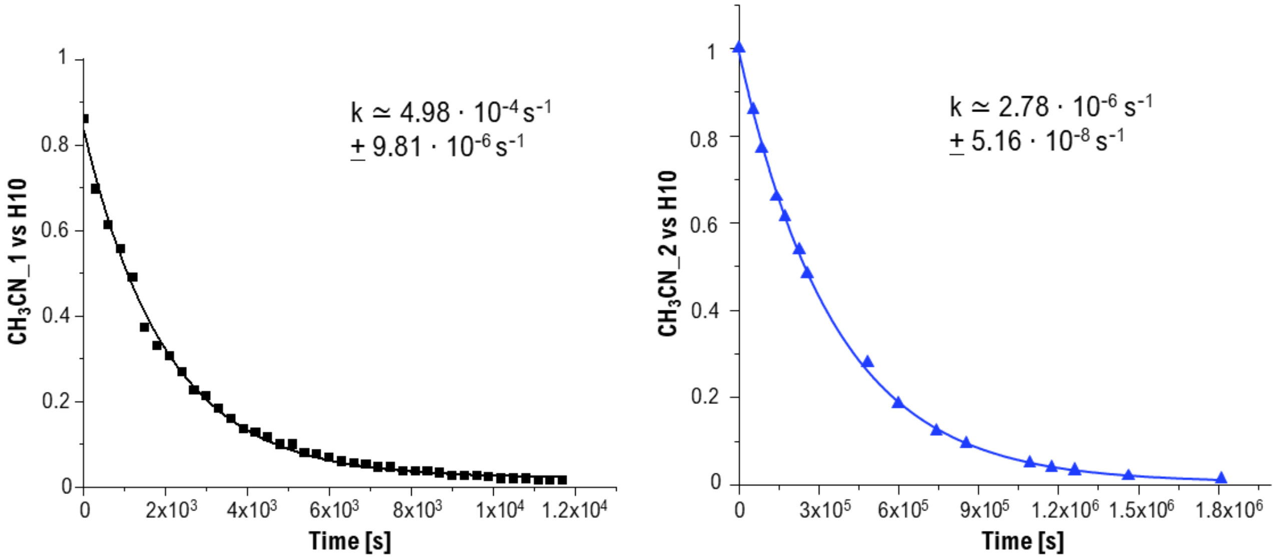

Figure S34. Nitrile exchange kinetics of $[Re(\eta^3\text{-}C_7H_7)(\eta^5\text{-}C_7H_9)(NCCH_3)_2]^+$ (**2**$^+$): CH_3CN_1 (left); CH_3CN_2 (right).

X-ray crystallography

Single-crystal X-ray diffraction data were collected at 160(1) K on a Rigaku OD Synergy-Hypix diffractometer (**2**$^+$, **4**$^+$), or on a Rigaku OD Supernova/Atlas diffractometer (**5**$^+$, [Re(CN-benzyl)$_6$]$^+$) or on a Rigaku OD Synergy/Pilatus diffractometer (**9**$^+$) using a single wavelength X-ray source (Cu Kα radiation: λ= 1.54184Å) using the copper X-ray radiation (λ = 1.54184 Å) from a dual wavelength X-ray source and an Oxford Instruments Cryojet XL cooler. The selected suitable single crystal was mounted using polybutene oil on a flexible loop fixed on a goniometer head and immediately transferred to the diffractometer. Pre-experiment, data collection, data reduction and analytical absorption correction[10] were performed with the program suite CrysAlisPro.[11] Using Olex2,[12] the structure was solved with the SHELXT[13] small molecule structure solution program and refined with the SHELXL2018/3 program package[14] by full-matrix least-squares minimization on F2. PLATON[15] was used to check the result of the X-ray analysis. CCDC 2308424-2308428 contain the supplementary crystallographic data for this paper. These data are provided free of charge by The Cambridge Crystallographic Data Centre via www.ccdc.cam.ac.uk/structures.

Special features

2: There is a mixture of counter anions in the crystal. PF_6^- and ReO_4^- share the same sites with site-occupancy factors of 0.9278(12) and 0.0722(12), respectively.

4: There is a mixture of counter anions in the crystal. PF_6^- and ReO_4^- share the same sites with site-occupancy factors of 0.9159(9) and 0. 0.0841(9), respectively.

[Re(CN-benzyl)$_6$]PF$_6$: The ions are located on a three-fold axis and on a center of inversion. The asymmetric unit contains about one sixth of the molecule, the rest is reproduced by the corresponding symmetry operations.

9: The ligand η^5-C_7H_9 is fully disordered over two sets of positions with a site-occupancy factor of 0.580(9) for the major part.

Table S1. Crystal data and data collection of complexes **2**0.928PF_6 0.072ReO_4 and **4**0.916PF_6, 0.084ReO_4.

	**[Re(η^3-C_7H_7)(η^5-C_7H_9)($NCCH_3$)$_2$]0.928$PF_6$0.072ReO_4 (2[0.928$PF_6$0.072ReO_4])**	**[Re(η^3-C_7H_7)(η^5-C_7H_9)(PTA)$_2$] 0.916PF_6,0.084ReO_4 (4[0.916PF_6,0.084ReO_4])**
Empirical formula	$C_{18}H_{22}F_{5.57}N_2O_{0.29}P_{0.93}Re_{1.07}$	$C_{26}H_{40}F_{5.5}N_6O_{0.34}P_{2.92}Re_{1.08}$
Formula weight	605.22	838.71
Temperature/K	160(1)	160.0(1)
Crystal system	monoclinic	triclinic
Space group	$P2_1/n$	P-1
a/Å	7.81784(5)	10.11517(15)
b/Å	11.76216(9)	12.02142(18)
c/Å	21.75940(14)	12.8682(2)
α/°	90	78.5941(13)
β/°	94.8456(6)	79.8415(13)
γ/°	90	73.8857(13)
Volume/Å^3	1993.73(2)	1461.27(4)
Z	4	2
ρ_{calc}g/cm^3	2.016	1.906
μ/mm^{-1}	14.001	10.896
F(000)	1163.0	830.0
Crystal size/mm^3	0.14 × 0.08 × 0.03	0.07 × 0.06 × 0.02
Radiation	Cu Kα (λ = 1.54184)	Cu Kα (λ = 1.54184)
2Θ range for data collection/°	8.156 to 154.732	7.066 to 154.736
Index ranges	$-9 \le h \le 9$, $-14 \le k \le 14$, $-27 \le l \le 25$	$-12 \le h \le 11$, $-15 \le k \le 13$, $-16 \le l \le 16$
Reflections collected	24123	30601
Independent reflections	4225 [R_{int} = 0.0217, R_{sigma} = 0.0131]	6143 [R_{int} = 0.0321, R_{sigma} = 0.0251]
Data/restraints/parameters	4225/255/302	6143/57/419
Goodness-of-fit on F^2	1.092	1.034
Final R indexes [I>=2σ (I)]	R_1 = 0.0212, wR_2 = 0.0539	R_1 = 0.0202, wR_2 = 0.0501
Final R indexes [all data]	R_1 = 0.0215, wR_2 = 0.0541	R_1 = 0.0214, wR_2 = 0.0507
Largest diff. peak/hole / e Å^{-3}	0.74/-0.83	1.34/-0.91
CCDC Nr.	2308424	2308425

Table S2. Crystal data and data collection of complexes **5**PF_6 and [Re(CN-benzyl)$_6$]PF_6.

	**[Re(η^3-C_7H_7)(η^5-C_7H_9)(dppe)$_2$]PF_6 (5PF_6)**	**[Re(CN-benzyl)$_6$]PF_6**
Empirical formula	$C_{40}H_{40}F_6P_3Re$	$C_{48}H_{42}F_6N_6PRe$
Formula weight	913.83	1034.04
Temperature/K	160(1)	160(1)
Crystal system	triclinic	trigonal
Space group	P-1	R-3
a/Å	10.7695(3)	13.03660(10)
b/Å	11.7398(3)	13.03660(10)
c/Å	16.3456(3)	22.1034(2)
α/°	71.015(2)	90
β/°	72.405(2)	90
γ/°	68.420(3)	120
Volume/Å^3	1777.58(9)	3253.26(6)
Z	2	3
ρ_{calc}g/cm^3	1.707	1.583
μ/mm^{-1}	8.489	6.404
F(000)	908.0	1548.0
Crystal size/mm^3	0.12 × 0.05 × 0.04	0.23 × 0.17 × 0.11
Radiation	Cu Kα (λ = 1.54184)	Cu Kα (λ = 1.54184)
2Θ range for data collection/°	5.846 to 153.02	8.794 to 152.694
Index ranges	-13 ≤ h ≤ 13, -14 ≤ k ≤ 14, -20 ≤ l ≤ 19	-16 ≤ h ≤ 16, -16 ≤ k ≤ 13, -27 ≤ l ≤ 27
Reflections collected	34898	7784
Independent reflections	7395 [R_{int} = 0.0265, R_{sigma} = 0.0220]	1525 [R_{int} = 0.0198, R_{sigma} = 0.0137]
Data/restraints/parameters	7395/148/488	1525/0/96
Goodness-of-fit on F^2	1.052	1.065
Final R indexes [I>=2σ (I)]	R_1 = 0.0219, wR_2 = 0.0532	R_1 = 0.0146, wR_2 = 0.0376
Final R indexes [all data]	R_1 = 0.0236, wR_2 = 0.0545	R_1 = 0.0146, wR_2 = 0.0376
Largest diff. peak/hole / e Å^{-3}	1.14/-1.19	0.29/-0.27
CCDC Nr.	2308426	2308428

Table S3. Crystal data and data collection of complexes [**9**]OTf.

	**[^{99}Tc(η^7-C_7H_7)(η^5-C_7H_9)]OTf ([9]OTf)**
Empirical formula	$C_{15}H_{16}F_3O_3STc$
Formula weight	431.34
Temperature/K	160.0(1)
Crystal system	monoclinic
Space group	$P2_1/n$
a/Å	10.8262(3)
b/Å	9.5679(3)
c/Å	15.7816(5)
α/°	90
β/°	107.719(3)
γ/°	90
Volume/Å^3	1557.17(9)
Z	4
ρ_{calc}g/cm^3	1.840
μ/mm^{-1}	9.179
F(000)	864.0
Crystal size/mm^3	0.08 × 0.05 × 0.02
Radiation	Cu Kα (λ = 1.54184)
2Θ range for data collection/°	8.8 to 148.986
Index ranges	-13 ≤ h ≤ 12, -11 ≤ k ≤ 11, -15 ≤ l ≤ 19
Reflections collected	13586
Independent reflections	3170 [R_{int} = 0.0483, R_{sigma} = 0.0362]
Data/restraints/parameters	3170/481/272
Goodness-of-fit on F^2	1.083
Final R indexes [I>=2σ (I)]	R_1 = 0.0372, wR_2 = 0.0964
Final R indexes [all data]	R_1 = 0.0422, wR_2 = 0.0994
Largest diff. peak/hole / e Å^{-3}	1.31/-0.79
CCDC Nr.	2308427

The nature of [Re(η^3-C_7H_7)(η^5-C_7H_9)(CN-^{t}Bu)$_2$]$^+$ (**6**$^+$) was also confirmed by an X-ray structural determination but the data was of insufficient quality. The structure has not been fully refined and has not been deposited.

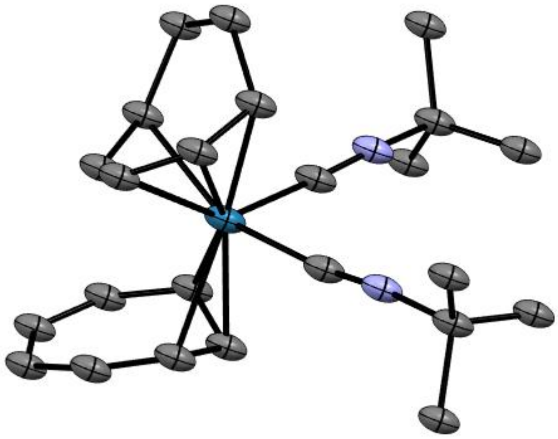

Figure S35. Molecular structure (50% probability ellipsoids) of [Re(η^3-C_7H_7)(η^5-C_7H_9)(CN- ^{t}Bu)$_2$]$^+$ (**6**$^+$).

HR-ESI-MS Spectra

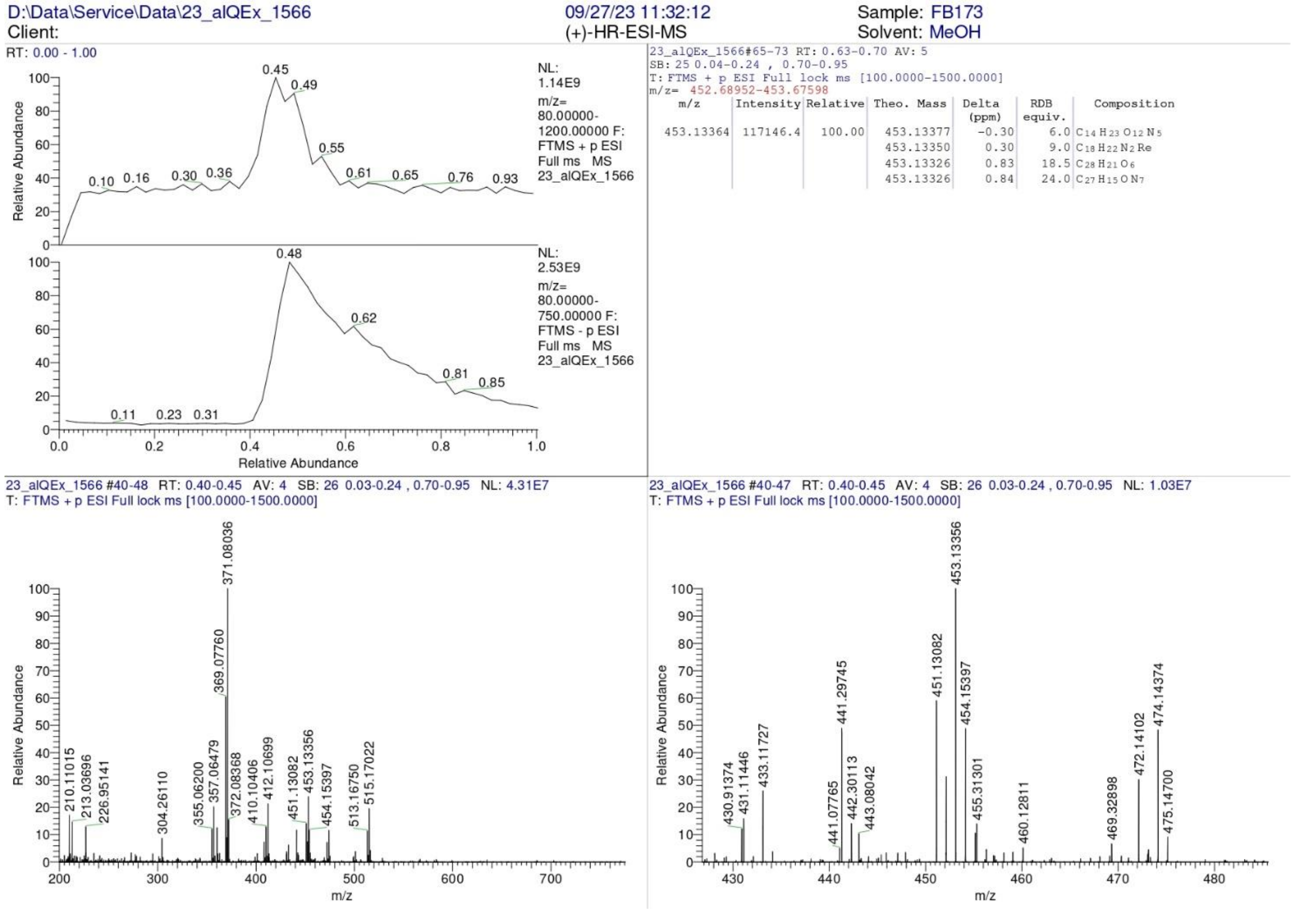

Figure S36. HR-ESI-MS spectrum of [Re(η^3-C_7H_7)(η^5-C_7H_9)($NCCH_3$)$_2$]$^+$ (**2**$^+$).

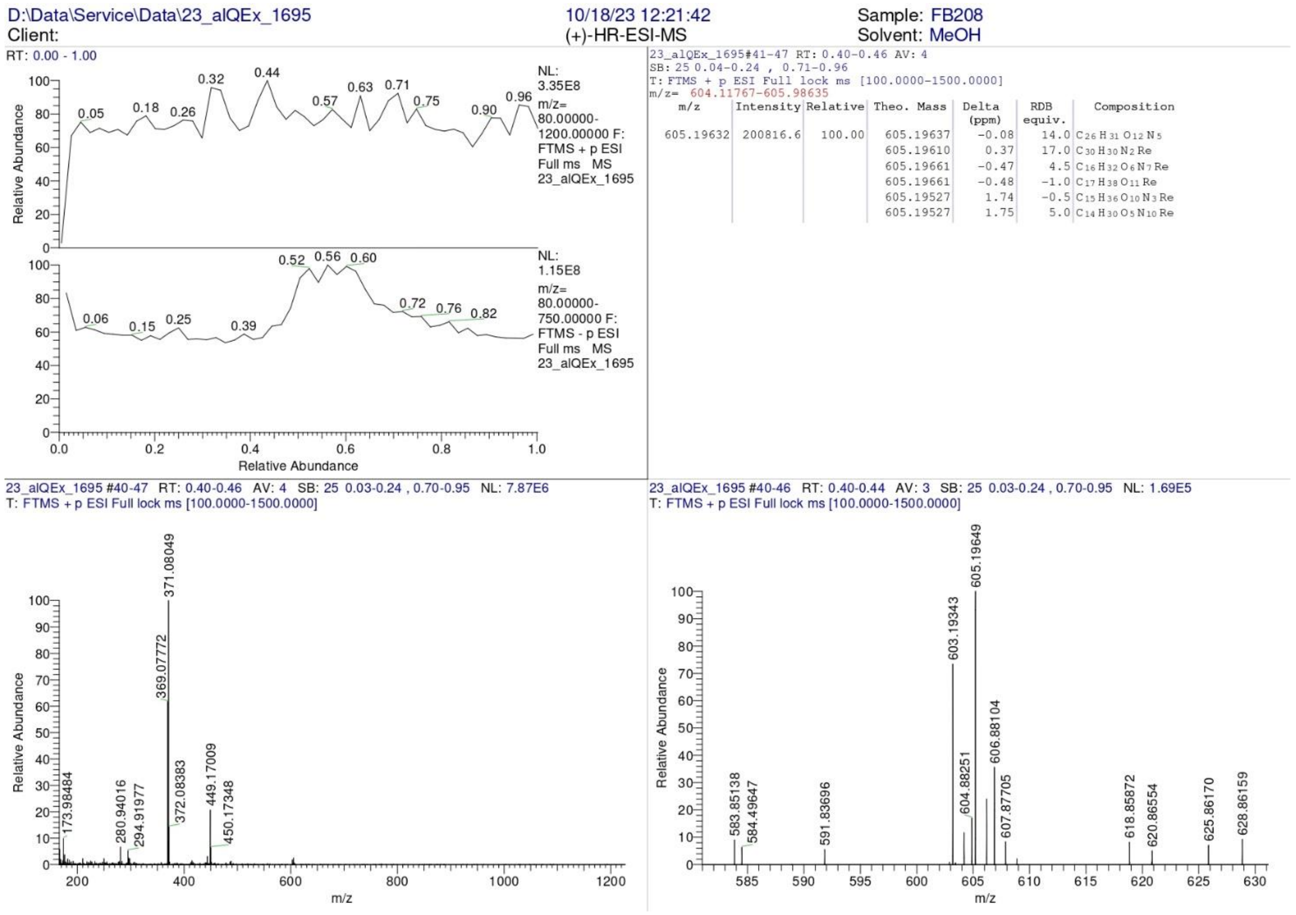

Figure S37. HR-ESI-MS spectrum of $[Re(\eta^3\text{-}C_7H_7)(\eta^5\text{-}C_7H_9)(NCCH_2Ph)_2]^+$ (**3**$^+$).

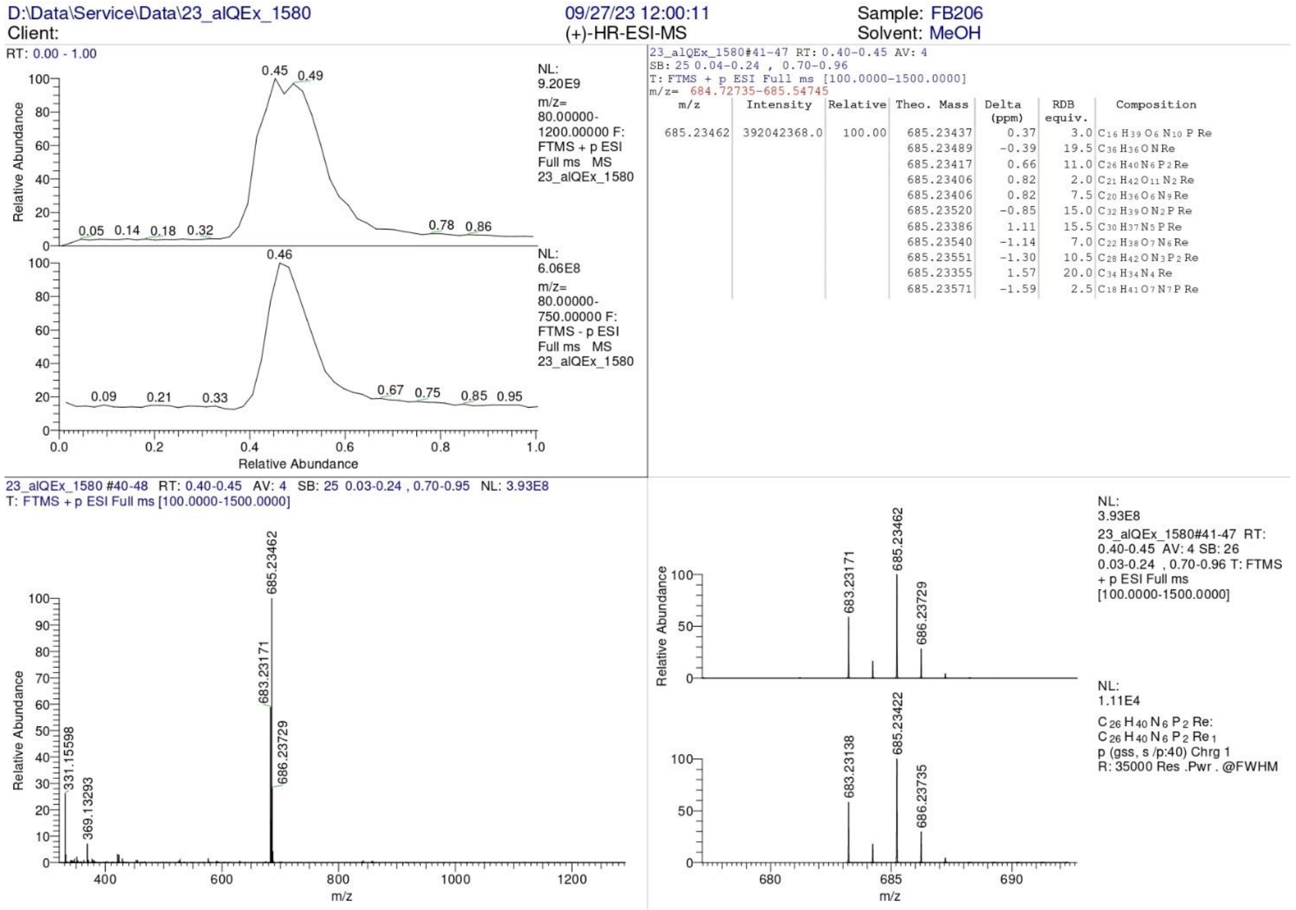

Figure S38. HR-ESI-MS spectrum of $[Re(\eta^3\text{-}C_7H_7)(\eta^5\text{-}C_7H_9)(PTA)_2]^+$ (**4**$^+$).

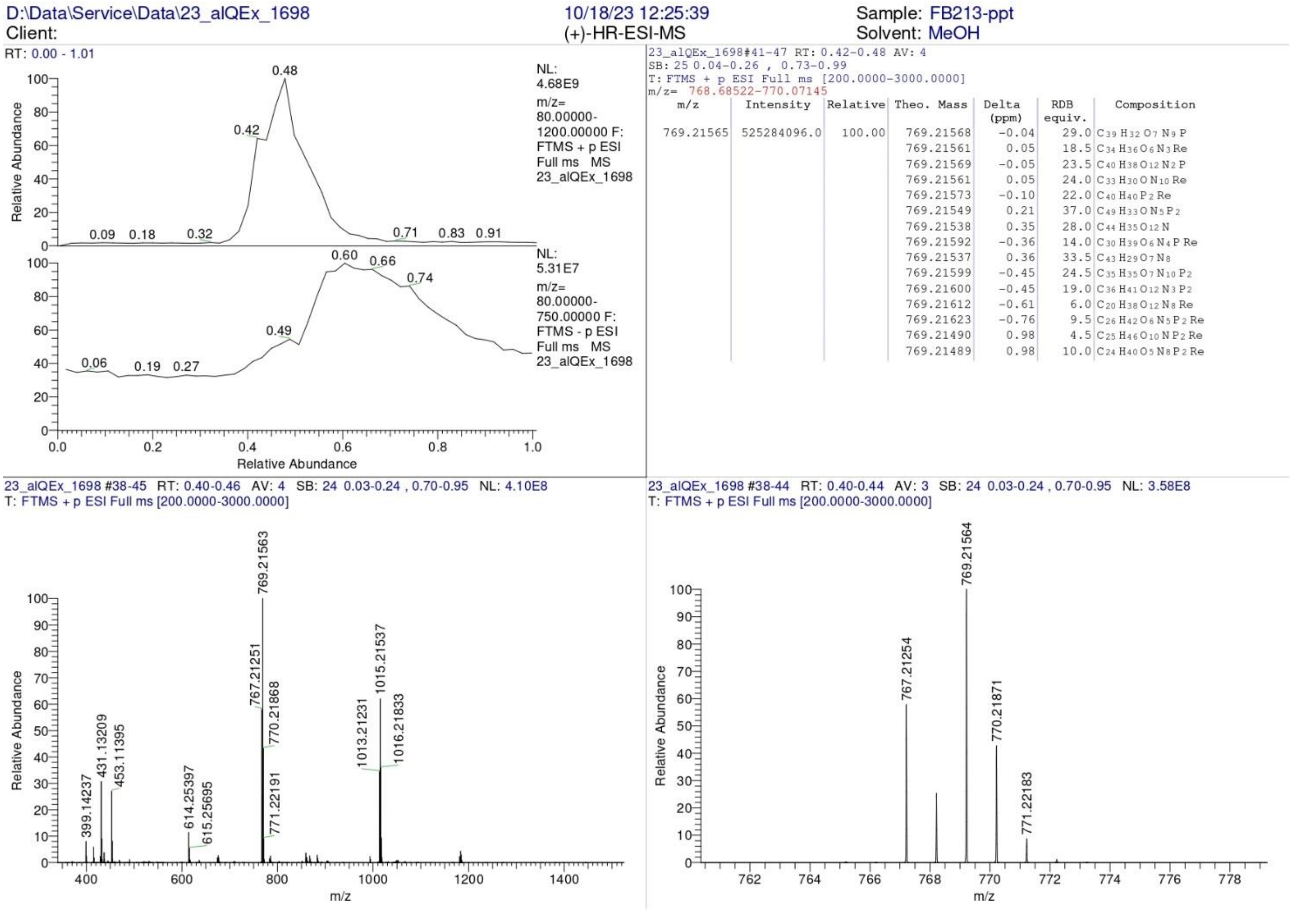

Figure S39. HR-ESI-MS spectrum of $[Re(\eta^3\text{-}C_7H_7)(\eta^5\text{-}C_7H_9)(dppe)]^+$ (**5**$^+$).

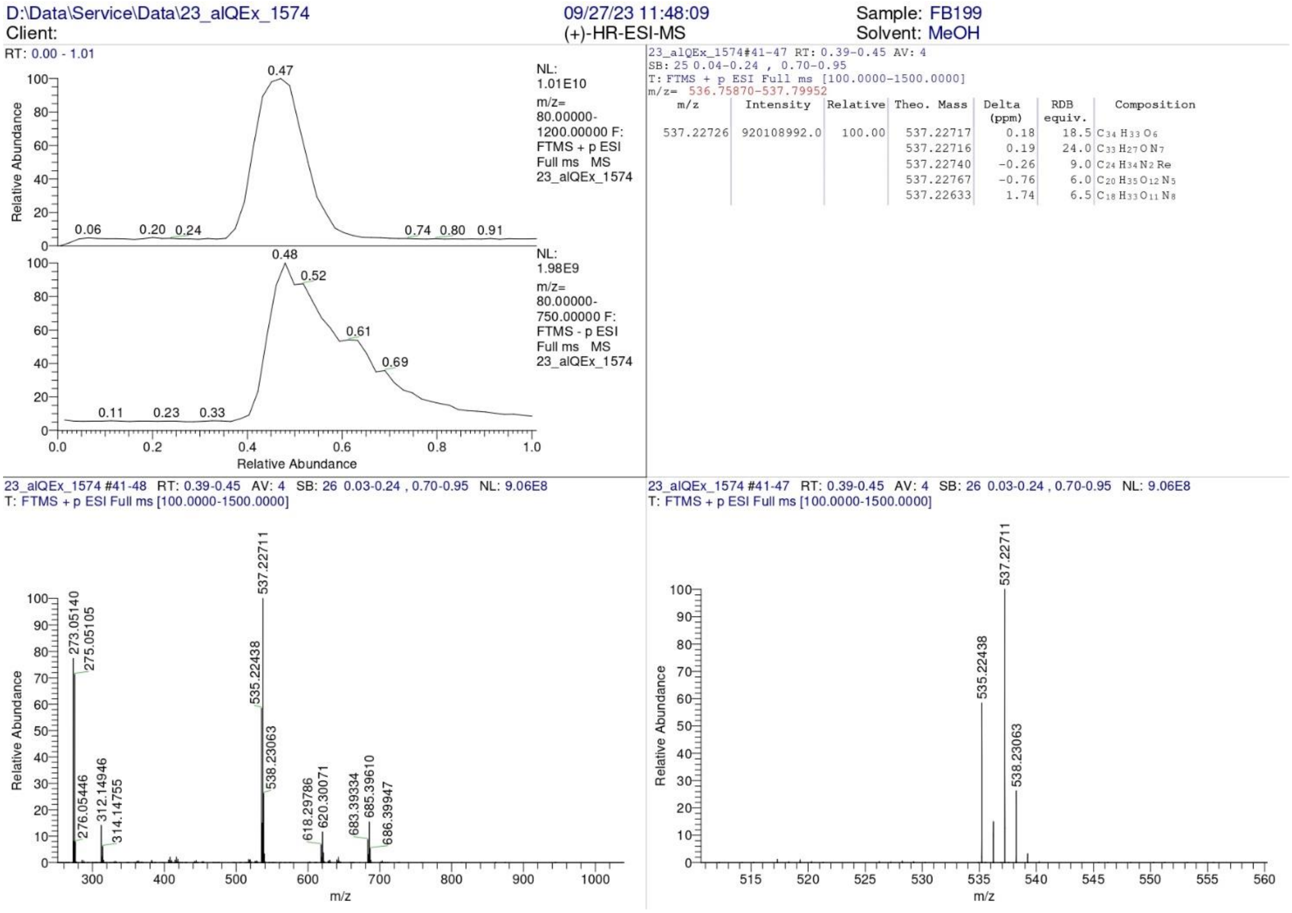

Figure S40. HR-ESI-MS spectrum of $[Re(\eta^3\text{-}C_7H_7)(\eta^5\text{-}C_7H_9)(CN\text{-}^tBu)_2]^+$ (**6**$^+$).

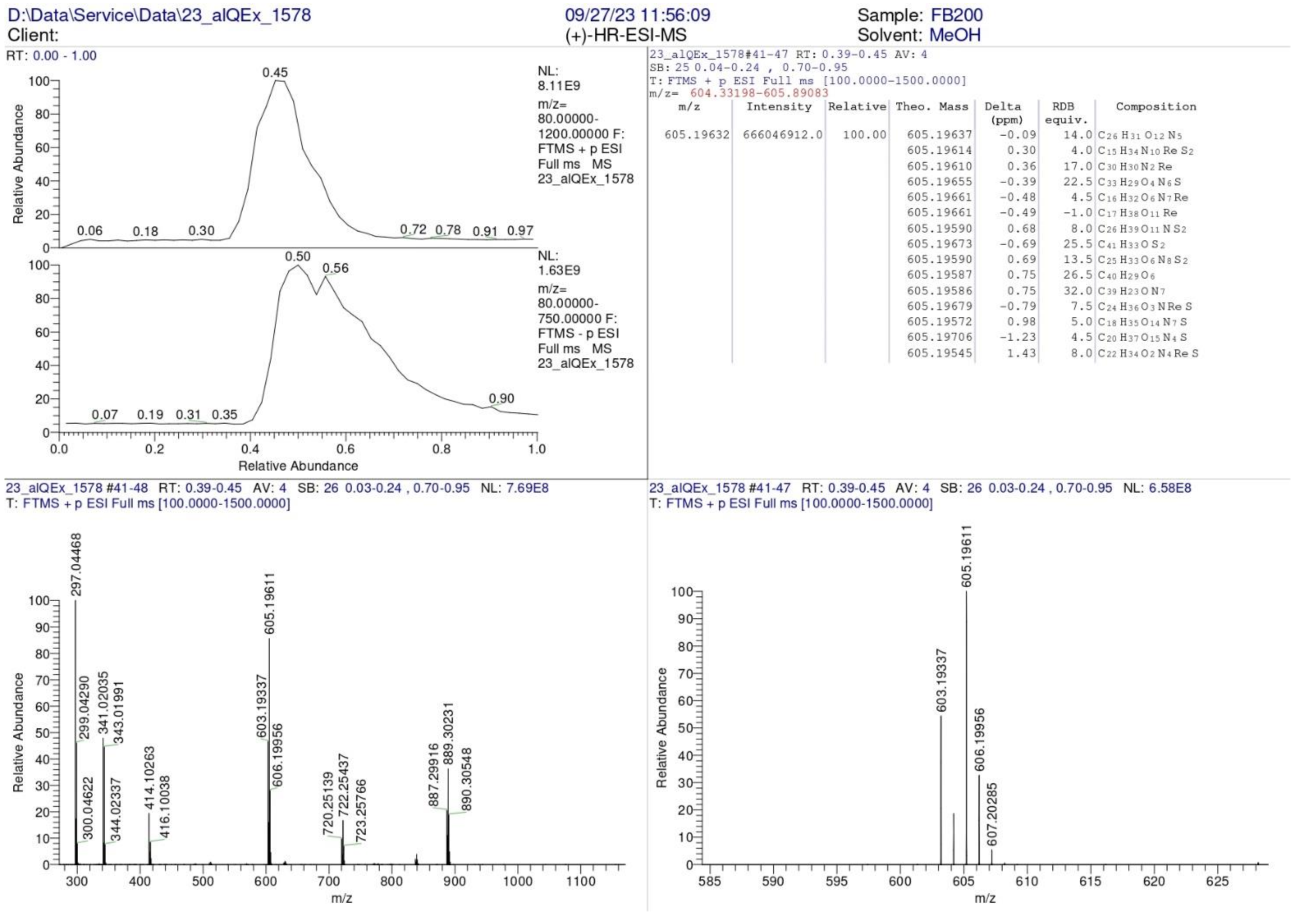

Figure S41. HR-ESI-MS spectrum of $[Re(\eta^3\text{-}C_7H_7)(\eta^5\text{-}C_7H_9)(CN\text{-benzyl})_2]^+$ (**7**$^+$).

References:

(1) Meola, G.; Braband, H.; Jordi, S.; Fox, T.; Blacque, O.; Spingler, B.; Alberto, R. Structure and Reactivities of Rhenium and Technetium Bis-Arene Sandwich Complexes [M(η^6-Arene)$_2$]$^+$. *Dalton Trans.* **2017**, *46* (42), 14631–14637.

(2) Benz, M.; Braband, H.; Schmutz, P.; Halter, J.; Alberto, R. From TcVII to TcI; Facile Syntheses of Bis-Arene Complexes [$^{99(m)}$Tc(Arene)$_2$]$^+$ from Pertechnetate. *Chem. Sci.* **2015**, *6* (1), 165–169.

(3) Hernández-Valdés, D.; Avignon, F.; Müller, P.; Meola, G.; Probst, B.; Fox, T.; Spingler, B.; Alberto, R. [Re(η^6-Arene)$_2$]$^+$ as a Highly Stable Ferrocene-like Scaffold for Ligands and Complexes. *Dalton Trans.* **2020**, *49* (16), 5250–5256.

(4) Gotzmann, C.; Blacque, O.; Fox, T.; Alberto, R. [Re(η^6-C_6H_5-benzimidazole)$_2$]$^+$ and Derivatives as Dye Mimics; Synthesis, UV Absorption Studies and DFT Calculations. *Eur. J. Inorg. Chem.* **2021**, *25*, 2493–2498.

(5) Do Thi, N. P.; Spichiger, S.; Paglia, P.; Bernardinelli, G.; Kündig, E. P.; Timms, P. L. Synthesis and Reactivity of Naphthalene Sandwich Complexes of Molybdenum. X-Ray Structure of [Mo(η^6-Naphthalene){P(OMe)$_3$}$_3$] and [Mo(H)(η^6-Naphthalene){P(OMe)$_3$}$_3$][BF$_4$]. *Helv. Chim. Acta* **1992**, *75* (8), 2593–2607.

(6) Kündig, E. P.; Timms, P. L., Chemistry of polydentate ligands. Part 5. Complexes of 2,9-di-hydrazino-derivatives of 1,10-phenanthroline. Dependence of co-ordination number of a ligand on the anion present, *J. Chem. Soc., Dalton Trans.* **1980**, 736-742.

(7) Csucker, J.; Jo, D. K.; Nadeem, Q.; Blacque, O.; Fox, T.; Braband, H.; Alberto, R. An Isoindoline Bridged [M(η^6-Arene)$_2$]$^+$ (M = Re, ^{99m}Tc) Ansa-Arenophane and Its Dinuclear Macrocycles with Axial Chirality. *Dalton Trans.* **2022**, *51* (25), 9591–9595.

(8) Battistin, F.; Fox, T.; Blacque, O.; Alberto, R. Reactivities of [Re(η^6-C_6H_6)(η^6-$C_{10}H_8$)]$^+$ and [Re(η^6-$C_{10}H_8$)$_2$]$^+$ with Cyclic, Nonaromatic Polyenes. *Organometallics* **2023**, *42* (9), 838–845.

(9) Boyd, G. E. Technetium and Promethium. ACS Publications **1959**, *36*, 3.

(10) Clark, R. C.; Reid, J. S. The Analytical Calculation of Absorption in Multifaceted Crystals. *Acta Crystallogr. Sect. A Found. Crystallogr.* **1995**, *51* (6), 887–897.

(11) CrysAlisPro (version 1.171.42.57a). Rigaku Oxford Diffraction Ltd. Yarnton, Oxfordshire, England, 2022.

(12) Dolomanov, O. V.; Bourhis, L. J.; Gildea, R. J.; Howard, J. A. K.; Puschmann, H. A Complete Structure Solution, Refinement and Analysis Program. *J. Appl. Crystallogr* **2009**, *42* (2), 339–341.

(13) Sheldrick, G. M. SHELXT–Integrated Space-Group and Crystal-Structure Determination. *Acta Crystallogr. Sect. A Found. Adv.* **2015**, *71* (1), 3–8.

(14) Sheldrick, G. M. Crystal Structure Refinement with SHELXLSheldrick, G. M. (2015). Crystal Structure Refinement with SHELXL. Acta Crystallographica Section C: Structural Chemistry, 71(1), 3–8. *Acta Crystallogr. Sect. C Struct. Chem.* **2015**, *71* (1), 3–8.

(15) Spek, A. L. Structure Validation in Chemical Crystallography. *Acta Crystallogr. Sect. D Biol. Crystallogr.* **2009**, *65* (2), 148–155.
